# Products of Photo- and Thermochemical Rearrangement of 19-Membered di-*tert*-Butyl-Azoxybenzocrown

**DOI:** 10.3390/molecules27061835

**Published:** 2022-03-11

**Authors:** Ewa Wagner-Wysiecka, Paulina Szulc, Elżbieta Luboch, Jarosław Chojnacki, Paweł Sowiński, Katarzyna Szwarc-Karabyka

**Affiliations:** 1 Department of Chemistry and Technology of Functional Materials, Faculty of Chemistry, Gdańsk University of Technology, Narutowicza Street 11/12, 80-233 Gdańsk, Poland; pauszul1@student.pg.edu.pl; 2 Department of Inorganic Chemistry, Faculty of Chemistry, Gdańsk University of Technology, Narutowicza Street 11/12, 80-233 Gdańsk, Poland; jaroslaw.chojnacki@pg.edu.pl; 3 Nuclear Magnetic Resonance Laboratory, Faculty of Chemistry, Gdańsk University of Technology, Narutowicza Street 11/12, 80-233 Gdańsk, Poland; pawel.sowinski@pg.edu.pl (P.S.); katszwar@pg.edu.pl (K.S.-K.)

**Keywords:** macrocycle, azo compound, photochemistry, rearrangement, tautomerism, spectroscopy, complexation

## Abstract

The preparation and characterization of products of the photochemical and thermochemical rearrangements of 19-membered azoxybenzocrowns with two, bulky, *tert*-butyl substituents in benzene rings in the *para* positions to oligooxyethylene fragments (*meta* positions to azoxy group, i.e., ***t*-Bu-19-Azo-O** have been presented. In photochemical rearrangement, two colored typical products were expected, i.e., 19-membered *o*-hydroxy-*m,m*′-di-*tert*-butyl-azobenzocrown (***t*-Bu**-**19-*o*-OH**) and 19-membered *p*-hydroxy-*m,m*′-di-*tert*-butyl-azobenzocrown (***t*-Bu**-**19-*p*-OH**). In experiments, two colored atypical macrocyclic derivatives, one 6-membered and one 5-membered ring, bearing an aldehyde group (***t*-Bu-19-al**) or intramolecular ester group (***t*-Bu-20-ester**), were obtained. Photochemical rearrangement led to one more macrocyclic product being isolated and identified: a 17-membered colorless compound, without an azo moiety, ***t*-Bu-17-*p*-OH**. The yield of the individual compounds was significantly influenced by the reaction conditions. Thermochemical rearrangement led to ***t*-Bu-20-ester** as the main product. The structures of the four crystalline products of the rearrangement—***t*-Bu**-**19-*o*-OH**, ***t*-Bu**-**19-*p*-OH**, ***t*-Bu-20-ester** and ***t*-Bu-17-*p*-OH**—were determined by the X-ray method. Structures in solution of atypical derivatives (***t*-Bu-19-al** and ***t*-Bu-20-ester**) and ***t*-Bu**-**19-*p*-OH** were defined using NMR spectroscopy. For the newly obtained hydroxyazobenzocrowns, the azo–phenol⇄quinone–hydrazone tautomeric equilibrium was investigated using spectroscopic methods. Complexation studies of alkali and alkaline earth metal cations were studied using UV-Vis absorption spectroscopy. ^1^H NMR spectroscopy was additionally used to study the cation recognition of metal cations. Cation binding studies in acetonitrile have shown high selectivity towards calcium over magnesium for ***t*-Bu**-**19-*o*-OH**.

## 1. Introduction

Macrocycles, bearing different functionalities, are often used in many areas, e.g., as drug carriers [1] or catalysts [2,3]. Macrocyclic compounds are also of interest as a synthetic targets, providing functional systems for sensors, e.g., [4,5], molecular devices and switches c.f. [6,7]. Such macrocycles bear additional functionalities to make them sensitive to external stimuli, e.g., pH, potential, light, etc. Often, a azobenzene moiety is attached as a peripheral or inherent part due to its photo or redox activity [8,9,10,11,12].

Macrocyclization is challenging; thus, different approaches are used for an effective synthesis of particular types of macrocyclic cores using the well-known high dilution technique and/or using various templates to more sophisticated and elegant strategies [13].

**Figure 1:**
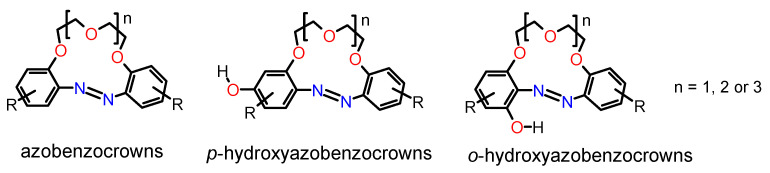
General chemical formulas of azobenzocrowns and hydroxyazobenzocrowns [14,15,16,17,18,19,20,21,22,23,24,25,26].

The synthesis of macrocycles, which combine the properties of azobenzene and crown ethers, i.e., photo and redox activity with metal cation complexation according to their size, is of interest for our group. Among those obtained so far include compounds called azobenzocrowns, comprising azobenzene fragments in the *o*,*o*′-positions, relative to polyether linkage (Figure 1) [9,14,15]. Until now, many derivatives of these class of compounds have been obtained and explored by our team, e.g., [16,17,18,19]. Currently, our efforts are focused on the preparation and study of the properties of hydroxyazobenzocrowns (Figure 1) [20,21,22,23,24,25,26].

Various procedures can lead to hydroxy–azo compounds, including macrocyclic procedures [20,21,22,23,24,25,26]. Among others, azoxy compounds can be transformed into hydroxy–azo compounds by a process known as Wallach rearrangement [27]. In this reaction, *p*-hydroxyderivatives are formed mainly, whereas under photo-illumination of azoxy derivatives, *o*-hydroxy-substituted azo compounds dominate [28]. Recently, we presented the products of the photochemical rearrangement of 19-membered azoxybenzocrown [26]. Our studies have shown that, besides the expected products of transformations of macrocyclic azoxy compounds, atypical ones can also be formed. We proved that the photochemical rearrangement of azoxybenzocrowns is a multidirectional process. We have demonstrated that the conditions of the photochemical process strongly affect the direction of the transformation and allows control over the yield of the desired macrocycles. Compared with classical chemical rearrangement, namely the Wallach rearrangement of azoxy compounds, such an approach seems to be a greener alternative for the preparation of hydroxyazobenzocrowns. Now, we present the preparation and characterization of products of the photo- and thermochemical rearrangements of 19-membered azoxybenzocrown with two, bulky, *tert*-butyl substituents in benzene rings, in the *para* positions to the oligooxyethylene fragments (*meta* positions to azo group), ***t*-Bu-19-Azo-O**. The aim of the study is mainly the investigation of the direction of the rearrangements of sterically hindered azoxy macrocycle and the characterization of the products.

## 2. Results and Discussion

### 2.1. Rearrangement of a 19-Membered Azoxybenzocrown with tert-Butyl Substituents in Para Positions to the Oligoether Linkage (Meta to Azoxy Group)

**Scheme 1:**
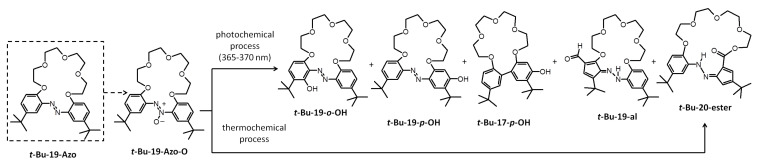
Identified products of the photo- and thermochemical rearrangement of 19-membered di-*t*-Bu-azoxybenzocrown ***t*-Bu-19-Azo-O**.

A number of photochemical rearrangement reactions for 19-membered azoxybenzocrown with *tert*-butyl substituents (***t*-Bu-19-Azo-O**) were carried out. The composition of the resulting post-reaction mixtures was analyzed. The main products of photo-rearrangement (and abbreviations used in this work) are shown in Scheme 1, and representative results are given in Table 1. Spectral characteristics of all the newly obtained compounds in this work, i.e., ^1^H, ^13^C and HR mass spectra, are included in the Supplementary Materials, Figures S1–S5.

The course of the photochemical reaction, i.e., the overall yield of macrocyclic compounds and the ratio of products, was studied. Under the established experimental conditions—power ~140 W, UV light 365–370 nm, 70 mg of substrate in 125 mL of solvent (the concentration of substrate not exceeding 1.12 × 10^−3^ M)—the influence of various solvents of different properties (experiments A–F, Table 1), with a small addition of acetic acid (experiments G and H, Table 1), was investigated. The highest total conversion of azoxy compounds to isolated and identified the four macrocyclic products—***t*-Bu-20-ester**, ***t*-Bu-19-al**, ***t*-Bu-19-*o*-OH** and ***t*****-Bu-19-*p*-OH**—was found to be a solvent-dependent conversion, and can be arranged in the following order: xylene > toluene > s-butanol > 2-propanol > DMF > n-butanol.

In the presence of acetic acid, the formation of ***t*-Bu-20-ester** and ***t*-Bu-19-al** was not observed; on the other hand, the amount of ***t*-Bu-19-*o*-OH** increased. Among the products of the photo-rearrangement, a colorless compound without an azo group, labeled as ***t*-Bu-17-*p*-OH**, was isolated and identified. This compound was mainly isolated from the products of photo-rearrangement carried out in toluene (yield approx. 4–5%). Its formation was also observed among products of rearrangement where xylene was used as a solvent.

Photochemical rearrangement of ***t*-Bu-19-Azo-O,** resulting in the formation of ***t*-Bu-19-*o*-OH,** seems to be favored in DMF (among all investigated neutral solvents). The effect of acetic acid addition is seen in reactions carried out in 2-propanol, where the yield of ***t*-Bu-19-*o*-OH** increases from 10.0 to 73.0%. In the reaction carried out in toluene, doped with acetic acid, the yield of ***t*-Bu-19-*o*-OH** increases from 45.4 to 66.7%. This is, to a certain degree, in agreement with results obtained by other authors for acyclic derivatives [29,30,31].

The highest, i.e., 70.0% yield, of ***t*-Bu-19-*p*-OH** was found in *s*-butanol. The relatively high yield of *p*-hydroxy–azo compound was distinctive in all cases where secondary alcohols were used as solvent.

**Figure 2:**
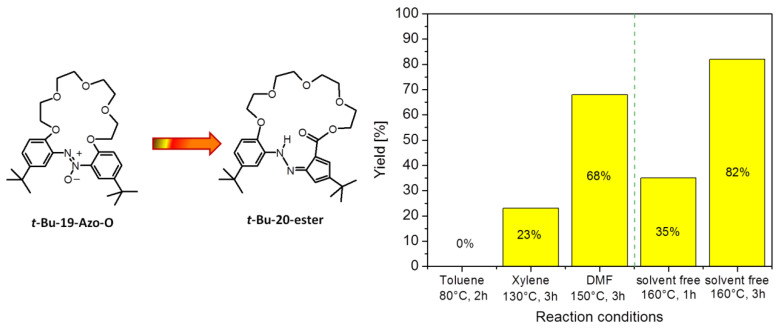
Yields of ***t*-Bu-20-ester** in thermal rearrangement of ***t*-Bu-19-Azo-O**.

Interestingly, among the above reaction products, other macrocyclic compounds, namely ***t*-Bu-20-ester** and ***t*-Bu-19-al,** formed as a result of the contraction of one of the six-membered aromatics to the five-membered cyclopentadiene derivative. Concomitantly, macrocycles with an aldehyde or ester group, respectively, were isolated and identified. The formation of these products was found mostly in experiments carried out in neutral solvents, but the yield of these compounds did not exceed 8%. However, one of these unusual rearrangement products, namely ***t*-Bu-20-ester,** can be obtained by thermal rearrangement of ***t*-Bu-19-Azo-O**. The yield of this transformation (Figure 2) is dependent on solvent type, temperature and the time of the conducting process; a 68% yield of ***t*-Bu-20-ester** was found when the reaction was carried out in DMF at 150 °C. Rearrangement conducted under solvent-free conditions at 160 °C allowed an 82% yield of this compound to be obtained.

The atypical products of rearrangement of azoxy compounds under both chemical- and photo-stimulated reactions have been reported by other authors [32,33] and by us [26].

### 2.2. X-ray Structures of Newly Obtained Macrocycles

The solid-state structures were denoted for four rearrangement products: ***t*-Bu-19-*o*-OH**, ***t*-Bu-19-*p*-OH**, ***t*-Bu-20-ester** and ***t*-Bu-17-*p*-OH**.

**Figure 3:**
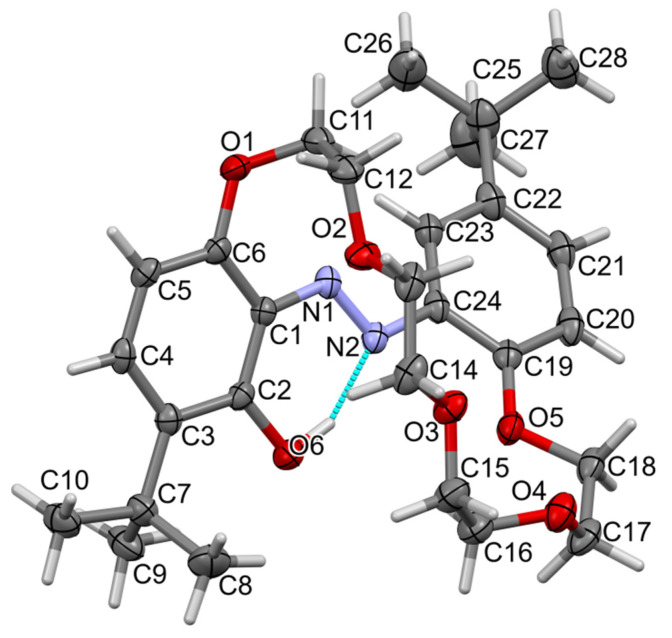
Molecular view of ***t*-Bu-19-*o*-OH** showing atom labelling scheme in one molecule, arbitrarily selected from the two symmetry-independent molecules in the structure. Intramolecular hydrogen bonding is drawn as the dashed cyan line. Displacement ellipsoids are drawn at a 50% probability level. Selected bond lengths (Å) and angles (°): N1-N2 1.276(2), N1-C1 1.401(2), N2-C24 1.422(2), C2-O6 1.352(2), C6-O1 1.370(2), C11-O1 1.449(2), C12-O2 1.422(2),C13-O2 1.416(2); valence angles: N2-N1-C1 115.36(16), N1-N2-C24 114.12(16), C6-O1-C11 119.59(15), C12-O2-C13 112.40(16), C14-O3-C15 111.65(16), C16-O4-C17 112.28(17), C18-O5-C19 116.71(16); torsions, O1-C6-C1-N1-4.4(3), N2-N1-C1-C6 173.41(16), O5-C19-C24-N2-0.5(3). Torsions in the linker ether: (starting from C6-O1-C11-C12) −sc, +sc, ap, ap, +sc, +ap, +ap, +sc, −ap, +ac, −sc and +ap (C17-C18-O5-C19). Code: sp synperiplanar 0–30°, sc synclinal 30–90°, ac anticlinal 90–150, ap antiperiplanar 150–180°.

**Figure 4:**
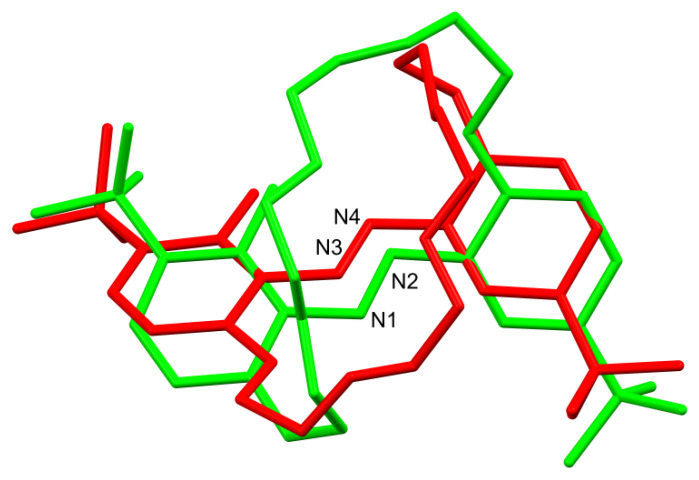
Comparison of the two symmetry-independent molecules of ***t*-Bu-19-*o*-OH**. Note the molecules are not just mutual mirror reflections since the ether linker has significantly different conformation. Colours by symmetry equivalence. The green molecule contains the N1-N2 group, and the red one, containing N3-N4, is inverted, and the best overlap of the molecules is presented (RMSD 1.7196 Å and maximum distance of equivalent atoms 4.023 Å, generated by Mercury 2021.2.0 program). Conformations of ether chains are visibly different.

The ***t*-Bu-19-*o*-OH** compound forms red crystals of monoclinic symmetry; its structure was solved and refined in the space group *P*2_1_/*n* (no. 14). A molecular view of one symmetry-independent molecule is presented in Figure 3. Asymmetric units contain two molecules (Figure 4), so the unit cell is built from eight molecules, *Z* = 8. The most characteristic feature of the molecule is the antiperiplanar position of both phenyl rings, which in turn makes the ether linker pass above the azo group. Such a conformation is stabilized by hydrogen bonding of the *ortho* OH group with the azo group (namely O6-H6…N2, cyclic motif *S*(6), Table 2). Relatively short N1-N2 and long C2-O6 distances confirm the presence of hydroxy–azo tautomer.

**Figure 5:**
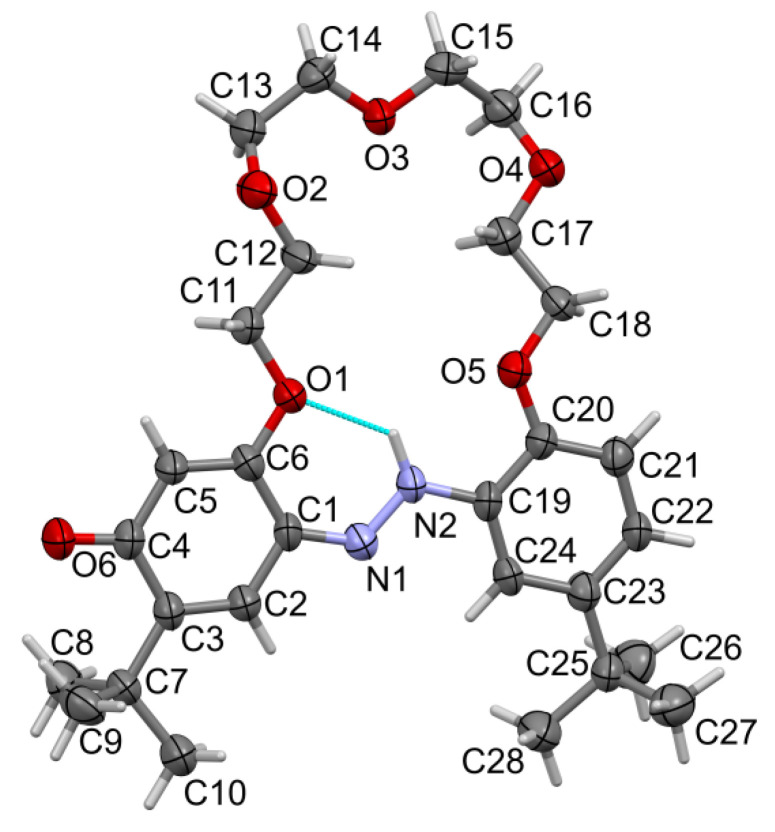
Molecular view of ***t*-Bu-19-*p*-OH** showing atom labelling scheme in one molecule, arbitrarily selected from the two symmetry-independent molecules in the structure. Intramolecular hydrogen bonding is drawn as the dashed cyan line. Displacement ellipsoids are drawn at a 50% probability level. Selected bond lengths (Å) and angles (°): N1-N2 1.315(6), N1-C1 1.325(7), N2-C19 1.413(7), C4-O6 1.242(6), C6-O1 1.360(6), C11-O1 1.439(6), C12-O2 1.437(6),C13-O2 1.407(7); valence angles: N2-N1-C1 122.4(4), N1-N2-C19 117.8(4), C6-O1-C11 115.6(4), C12-O2-C13 115.9(5), C14-O3-C15 112.1(4), C16-O4-C17 113.9(4), C18-O5-C20 117.3(4); torsions O1-C6-C1-N1 1.5(9), N2-N1-C1-C6 1.2 (9), O5-C20-C19-N2-1.8(8). Torsions in the linker ether: (starting from C6-O1-C11-C12) −ap, −ap, −ac, −sc, +sc, +ap, −ap, −sc, +sc, +ap, +ap and +ap (C17-C18-O5-C20).

Compound ***t*-Bu-19-*p*-OH** forms a needle with red crystals, with the symmetry of the triclinic system, and the space group $P\bar{1}$ (no. 2). Asymmetric units contain two molecules, and the whole unit cell is built from four molecules, Z = 4. The two independent molecules are related by a non-crystallographic inversion center at point (0.586, 0.632, 0.340) and its equivalents. One of the independent molecules is shown in Figure 5. The differences in the geometry of the two molecules (e.g., bond length differences of ca. 0.003 Å) can be ignored so we describe just one of them. The relatively short C4-O6 (of ca. 1.2 Å) and long N1-N2 bond lengths suggest that the quinone–hydrazone tautomer is dominating in the solid state. Additional stabilization of the molecule is due to intramolecular 6-membered cyclic hydrogen bonding, S(6)-type motif.

**Figure 6:**
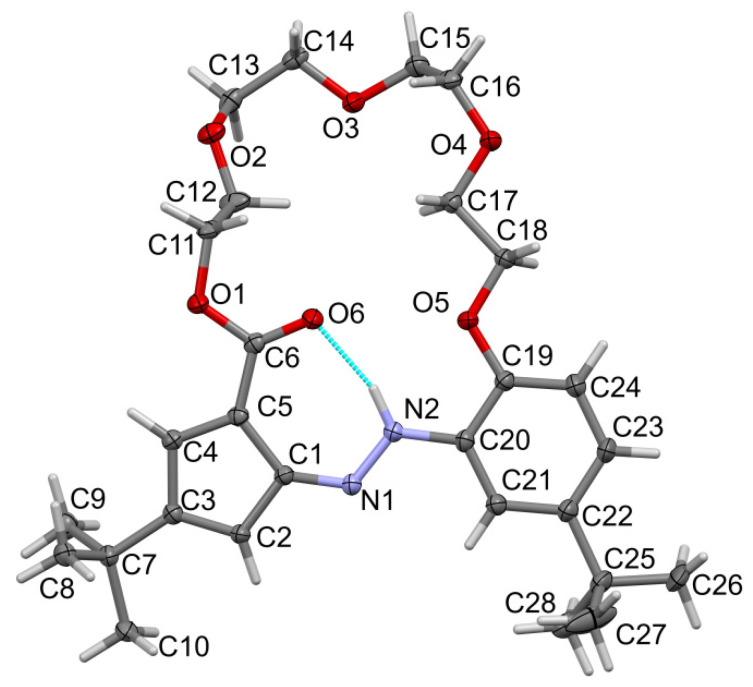
Molecular view of ***t*-Bu-20-ester** showing atom labelling scheme. Intramolecular hydrogen bonding is drawn as the dashed cyan line. Displacement ellipsoids are drawn at a 50% probability level. Selected bond lengths (Å) and angles (°): N1-N2 1.317(3), N1-C1 1.322(4), N2-C20 1.400(4), C6-O6 1.225(4), C6-O1 1.346(3), C11-O1 1.451(3), C12-O2 1.413(4), C13-O2 1.427(4), C18-O5 1.425(3), C19-O5 1.375(3); valence angles: N2-N1-C1 120.2(2), N1-N2-C20 120.5(2), C5-C6-O6 126.1(3), O1-C6-O6 121.6(3), C6-O1-C11 116.7(2); torsions: C1-N1-N2-C20-177.5(3), N1-N2-C20-C21-8.8(4), N2-N1-C1-C5 0.7(5). Torsions in the linker ether: (starting from C6-O1-C11-C12) +sc, +ap, −ap, −ac, +sc, −ap, +ac, +sc, −sc, −ap, +ap, and +ap (C17-C18-O5-C19).

The ***t*-Bu-20-ester** compound also forms needles with red crystals, but with the symmetry of the monoclinic system, and the space group *P*2_1_*/c* (no. 14). The asymmetric unit contains one molecule and the whole unit cell is built from four molecules, *Z* = 4. Most of the bond lengths and angles are in the expected ranges (Figure 6). Carbonyl bond C6-O6 is shorter, indicating its mainly double character. The bond length of N1-N2 is similar to the one found in ***t*-Bu-19-*p*-OH**. This time we find intramolecular 7-membered cyclic hydrogen bonding. Bonds C2-C3 and C4-C5 are a bit shorter than other bonds in 5-membered ring, as expected. The ether linker is less flat than that in ***t*-Bu-19-*p*-OH**.

**Figure 7:**
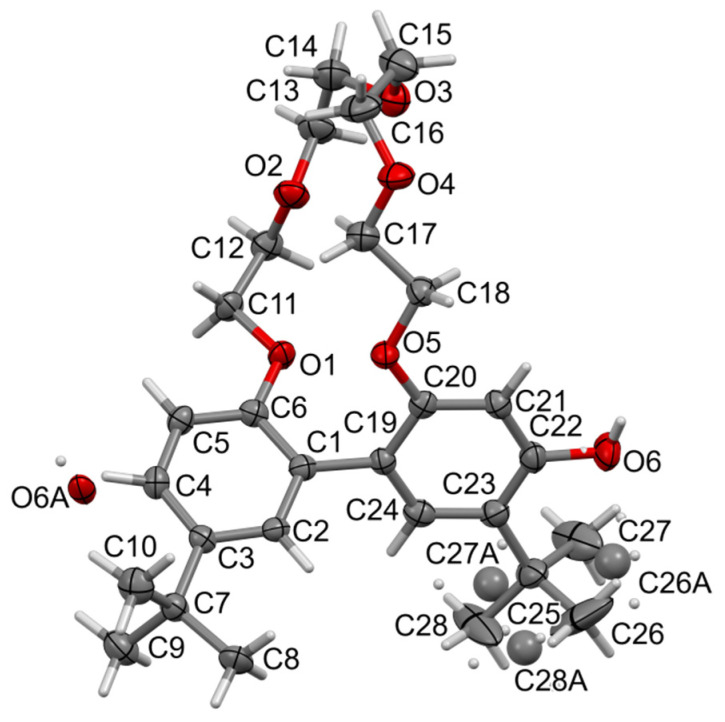
Molecular view of ***t*-Bu-17-*p*-OH** showing atom labelling scheme. Less populated disorder part atoms drawn as disconnected balls. Displacement ellipsoids drawn at 50% probability level. Selected bond lengths (Å) and angles (°): O6-C22 1.377(5), O1-C6 1.373(3), O1-C11 1.428(3), O2-C12 1.420(4), O2-C13 1.417(4), O3-C14 1.429(4), O3-C15 1.421(4), O5-C18 1.420(4), O5-C20 1.368(4), C1-C19 1.491(4); valence angles: C6-O1-C11 115.5(2), C13-O2-C12 112.4(2), C15-O3-C14 114.1(3), C17-O4-C16 115.1(3), C20-O5-C18 119.0(2), C6-C1-C19 122.7(3), C1-C19-C20 120.8(3); torsions: C2-C1-C19-C24 57.2(4), C11-O1-C6-C5-9.2(4), C18-O5-C20-C21 11.6(4). Torsions in the linker ether: (starting from C6-O1-C11-C12) +ap, −sc, −ap, +ap, +sc, −ap, +ac, +sc, −ac, +ap, +ap and +ap (C17-C18-O5-C20).

The ***t*-Bu-17-*p*-OH** compound forms colourless plate crystals with the structure symmetry of the monoclinic system, and the space group *P*2_1_*/c* (no. 14). The asymmetric unit contains one molecule and the whole unit cell is built from four molecules, Z = 4. Most of the bond lengths and angles are in the expected ranges (Figure 7). Only one ring is substituted by an OH group; however, molecules are packed in crystal being overlapped as disordered over two positions in proportion 0.626(5)/0.374(5). The hydroxyl group is involved in hydrogen bonding to an O-atom from the neighbour molecule ether linker (Table 2 with hydrogen bond details).

**Figure 8:**
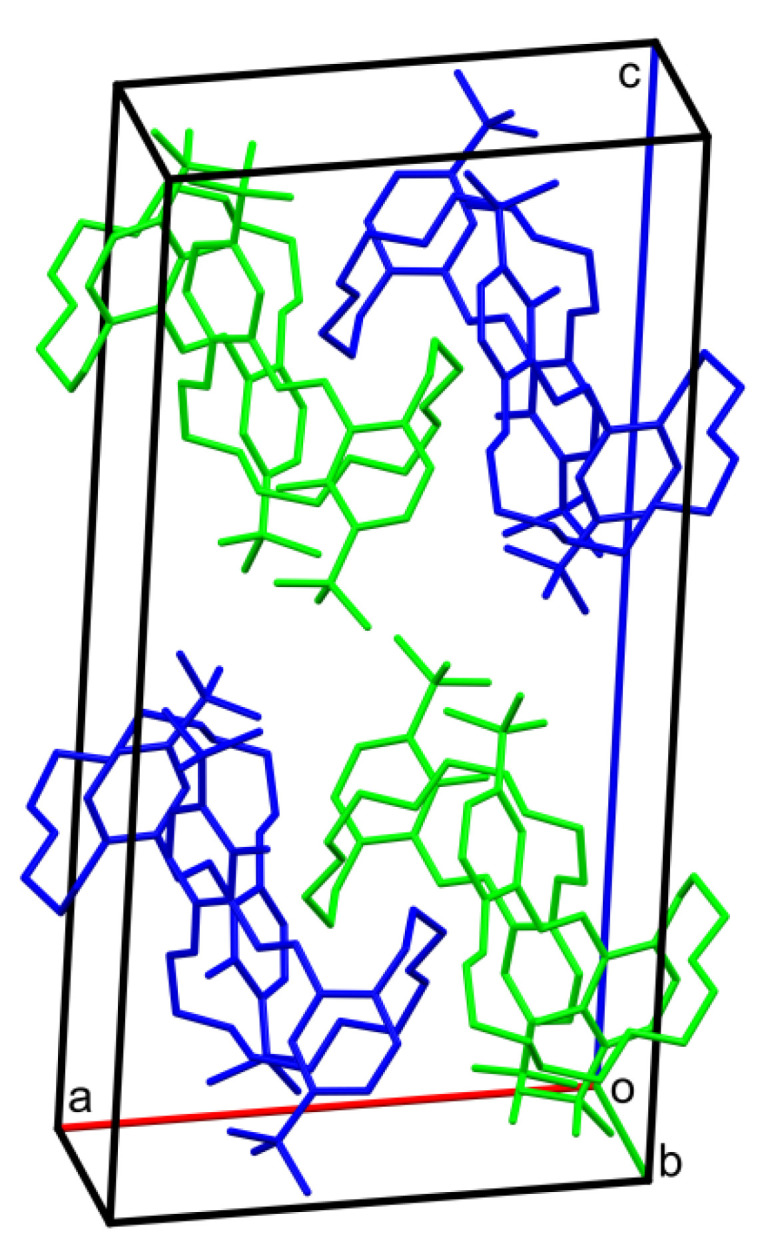
Crystal packing of ***t*-Bu-19-*o*-OH**, molecules are colored by symmetry equivalence. Hydrogen atoms are omitted.

**Figure 9:**
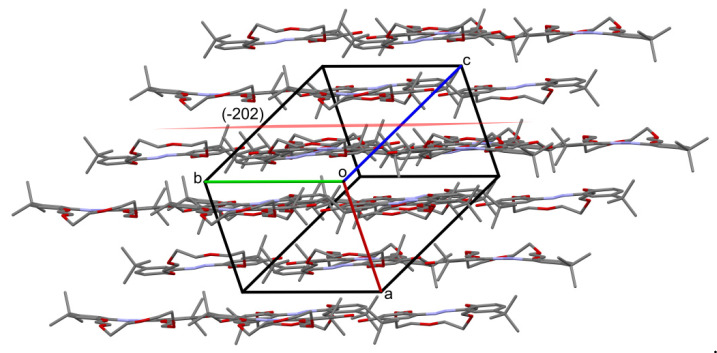
Crystal packing of ***t*-Bu-19-*p*-OH,** flat molecules form layers parallel to (-202) family of planes. Hydrogen atoms are omitted.

**Figure 10:**
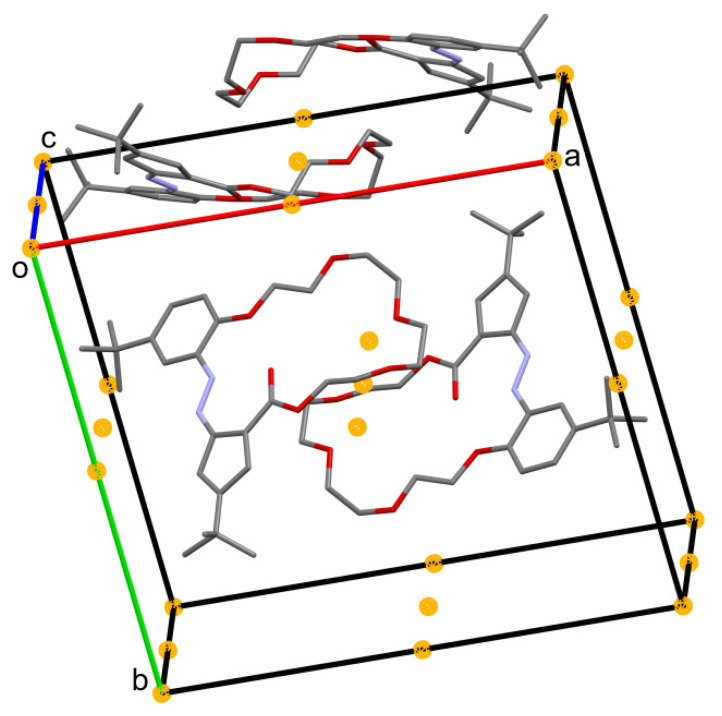
Crystal packing of ***t*-Bu-20-ester**. All crystal centers of symmetry present in unit cell are drawn as orange balls, and the glide planes and screw axes are omitted.

**Figure 11:**
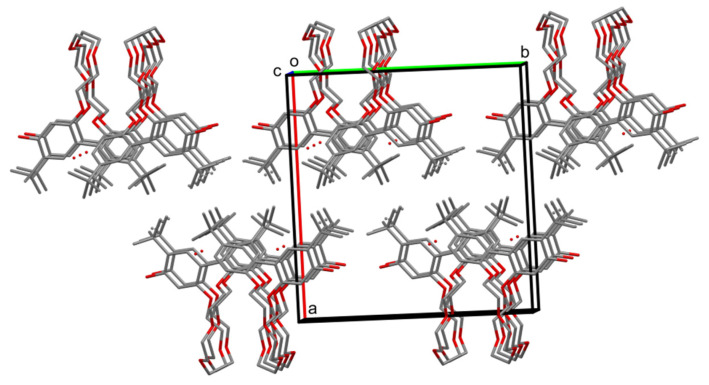
Crystal packing of ***t*-Bu-17-*p*-OH**. The molecules with folded ether linker are gathered into chains in (001) direction and then into layers parallel to (100) planes. The next layer fills the gaps between the ether linkers in the **c** axis direction with its etheric parts (omitted for clarity); this is equivalent to a parallel shifting, upwards of the bottom layer in the picture. This process forms hydrophobic and hydrophilic regions, like those in ***t*-Bu-19-*o*-OH**. Hydrogen atoms are omitted.

Crystal packing in the investigated structures is shown in Figure 8, Figure 9, Figure 10 and Figure 11.

In crystal the packing of ***t*-Bu-19-*o*-OH** (Figure 8), no significant stacking interactions can be found (shortest distance between ring centroids Cg...Cg is equal to 4.904(13) Å). Since there are no possible classical intermolecular hydrogen bonding routes, the molecules in the solid state interact mainly by non-specific van der Waals forces. Hydrophobic and hydrophilic regions can be found in planes parallel to (001).

Crystal packing in ***t*-Bu-19-*p*-OH** is dominated by stacking interactions between the aromatic rings of the same nature (for rings C19–C24 and C29–C34, between two symmetrically independent molecules, distance between ring centroids Cg, ..., Cg is equal to 3.725(4) Å). Molecules are almost flat and form layers parallel to the crystal planes with Miller indices (-202), see Figure 9.

No stacking interactions were found in the ***t*-Bu-20-ester**, minimal Cg, ..., Cg is equal to 4.238(2) Å. Instead, each two molecules, with folded ether linker, are gathered around a symmetry center. The “dimers” are stack in the solid, not in parallel, but being mutually inclined, see Figure 10. Two molecules with folded ether linker are gathered around a symmetry center. The “dimers” are then stacked, not in parallel, but mutually inclined in the solid. Hydrogen atoms are omitted.

Packing of ***t*-Bu-17-*p*-OH** is not influenced by any stacking interactions, because the shortest distance between centroids of C1-C6 and its symmetry equivalent (by the glide plane) is equal to 5.013(2) Å. In fact, it is similar to the previously described ***t*-Bu-19-*o*-OH**. Molecules are arranged into layers parallel to (100) plane with aromatic rings at one side and the ether linkers at the other. The next layer is in the opposite orientation, forming a structure like a lipid bilayer, hydrophilic parts of molecules inside and hydrophobic parts of molecules outside (Figure 11). The orientation of the layers in ***t*-Bu-19-*o*-OH** was different (001); although, it was refined in a different space group setting. The entanglement of the ether parts is also different in both structures.

In Table 2, the parameters characterizing the hydrogen bonds in the structures of ***t*-Bu-19-*o*-OH**, ***t*-Bu-19-*p*-OH**, ***t*-Bu-20-ester** and ***t*-Bu-17-*p*-OH** are collected.

### 2.3. Studies of the Structures in Solution

**Figure 12:**
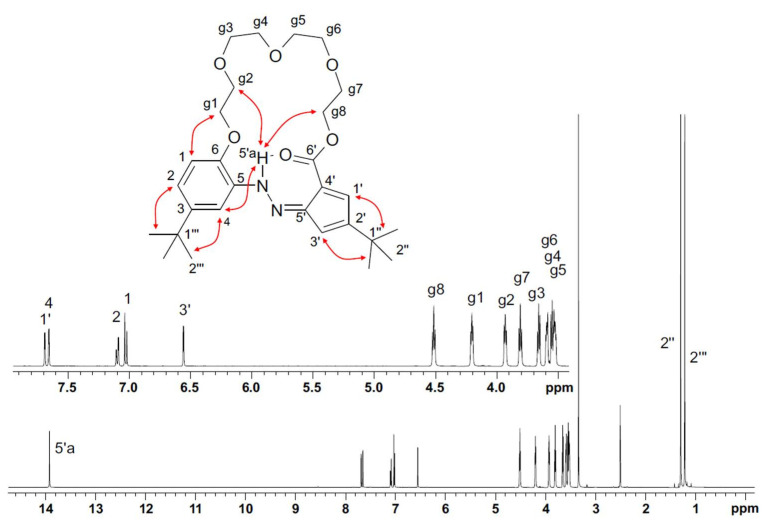
^1^H NMR (DMSO-*d_6_*) spectrum of ***t*-Bu-20-ester** with labelling signals of individual protons and with the main ROE correlations shown.

To investigate the structure of ***t*-Bu-20-ester** similarity with the X-ray structure, compound was studied in solution using NMR spectroscopy (Figure 12). The ^1^H and ^13^C NMR spectra (DMSO-*d_6_*) of ***t*-Bu-20-ester** were interpreted based on the H/C correlation spectra (gHSQC and gHMBC). The cyclic nature of ***t*-Bu-20-ester** favors close to antiperiplanar conformations of the hydrazone moiety. The very weak ROE H-5’a/H-4 effect, comparable to the H-5’a/H-g2 and H-5’a/H-g8 effects, and the strong influence of hydrogen bonding on the chemical shift of H-5’a, are in full agreement with the conformation determined by the X-ray method (Figure 6 and Figure 10), in which H-5’a hydrazone proton strongly interacts with the macrocyclic ring. The main ROE correlations are presented in the corresponding structure in Figure 12.

**Figure 13:**
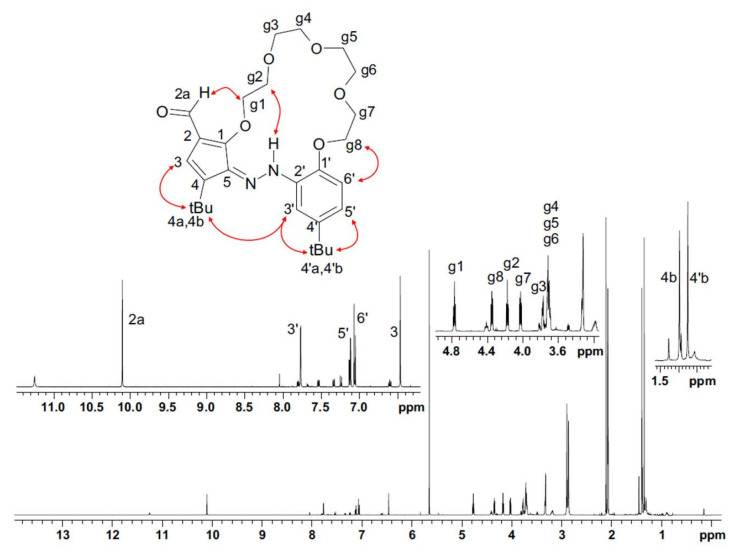
^1^H NMR (acetone-*d_6_*) spectrum of ***t*-Bu-19-al** with labelling signals of individual protons and with the main ROE correlations shown.

Structure of the compound ***t*-Bu-19-al** was also determined from the NMR spectra. Long-range H/C scalar correlations showed the presence of a five-membered ring, C1-C5, substituted with two heteroatoms, *t*-Bu and aldehyde group, at C-1, C-5, C-4 and C2 atoms, respectively. The ether linkage of the g1 group with C-1 atom of the ring was determined through the H-g1/C-1 correlation, observed in the gHMBC spectrum. The aldehyde group was located through the H-2a/C2 correlation, as well as through the H-2a/H-g1 ROE effect observed in the ROESY spectrum. In a similar manner (based on the H/C and ROE correlation), the substituents on the C1′-C6′ ring were identified and located. General conformation of the macrocycle was established using the ROE effects shown in Figure 13.

***t*-Bu-19-al** in solution (DMSO-*d_6_*, Figure S4a) exists in one *Z* form, in contrast to the compound **19-al** [26], where there are two forms—*Z* and *E*—in the solution, with a predominance of the form *E*. Only one of them, *Z*, was found in the crystalline solid state [26]. This may be due to the different positioning of the aldehyde group relative to the hydrazone group.

### 2.4. Tautomeric Equilibrium of Hydroxyazobenzocrowns

**Figure 14:**
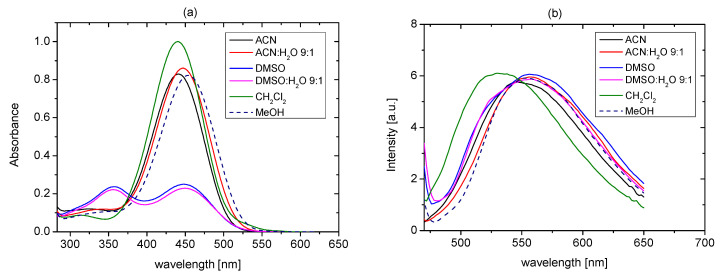
(**a**) UV-Vis absorption and (**b**) emission spectra of ***t*-Bu-19-*p*-OH** (2.6 × 10^−5^ M) in solvents of various properties.

The effect of solvent on the tautomeric equilibrium of ***t*-Bu-19-*p*-OH** was studied with spectroscopic methods. ^1^H NMR spectrum of ***t*-Bu-19-*p*-OH** (1.43 × 10^−5^ M) in acetonitrile-*d*_3_ shows that the quinone–hydrazone form dominates (90%), whereas the azo–phenol tautomer is present in 10% (Figure S1a–d). The most characteristic signals, observed as singlets, are peaks which can be attributed to NH proton signal (at 11.71 ppm in acetonitrile-*d*_3_) and the peak assigned to the CH proton of the quinone–hydrazone ring in the *orto* position to ether linkage (at 5.88 ppm in acetonitrile-*d*_3_). In DMSO-*d*_6_ azo–phenol form, with the characteristic signal of the OH proton at 10.16 ppm, dominates (80%) (Figure S1e–i). Tautomers also characterize the different spectral patterns of ether proton signals (region of 4.4-3.8 ppm). The distribution of tautomeric forms in solvents of various properties is different for ***t*-Bu-19-*p*-OH** than it was for **19-*p*-OH,** investigated earlier [26]. **19-*p*-OH** in DMSO-*d*_6_ exists in azo–phenol form, whereas in acetonitrile-*d*_3_ and acetone-*d*_6_, both tautomers exist under equilibrium with the tautomers ratios dependent on the type of solvent. The difference comparing in the tautomeric equilibrium with **19-*p*-OH** is also observed in UV-Vis spectra, where experiments are carried out at lower than in NMR spectroscopy concentrations. UV-Vis absorption spectra of ***t*-Bu-19-*p*-OH,** registered in most investigated solvents (Figure 14a), presented the band characteristics for the quinone–hydrazone form, located at ~450 nm. The azo–phenol form (peak at ~390 nm), under measurement conditions, was observed in equilibrium with the quinone–hydrazone form in DMSO (48%) and in 49% in a DMSO:water mixture (9:1). The UV-Vis characteristic bands of ***t*-Bu-19-*p*-OH** are presented in Tables S1 and S2.

The position of emission maxima is not strongly affected by the solvent type; the exception is dichloromethane, in which the emission band is observed at lower wavelength (Figure 14b). The comparison of two *p*-hydroxyazobenzocrowns of the same 19-membered macroring—namely **19-*p*-OH** and ***t*-Bu-19-*p*-OH**—points out that tautomeric equilibrium of *p*-hydroxyazobenzocrowns can also be significantly affected by the additional factor, i.e., the presence of bulky substituent in the *ortho* position to the hydroxyl moiety. This issue is the subject of the current research, concerning this group of compounds.

**Figure 15:**
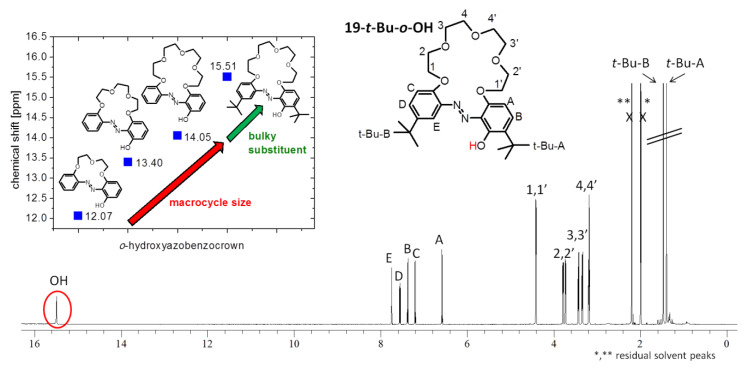
^1^H NMR spectrum of ***t*-Bu-19-*o*-OH** (6.3 × 10^−3^ M acetonitrile-*d_3_*). Inset: the relationships between the values of chemical shifts of the OH proton signal in ^1^H NMR spectra (acetone-*d_6_*) of *o*-hydroxyazobenzocrowns of different macrocycle sizes.

***t*-Bu**-**19-*o*-OH,** similarly to all investigated so far, in *orto* hydroxyazobenzocrowns [24,26], exists in the azo–phenol form in solution with characteristic absorption band in UV-Vis spectrum at 368 nm (acetonitrile, Table S1). The position of the OH proton signal in ^1^H NMR spectrum (acetonitrile-*d_3_*) at 15.49 ppm points out a strong intramolecular hydrogen bond (Figure 15). In solution, ***t*-Bu-19-*o*-OH** exists only in one form. Only one set of sharp signals for the discussed compound is observed in NMR spectra. The high value of the chemical shift of the hydroxyl group, above 15 ppm (Figure 15 and Supplementary Materials, Figure S2a,b), suggests conformational stabilization by strong intramolecular hydrogen bonding of the *ortho* OH group, with the azo group also in solution. Taking into consideration the spatial models of ***t*-Bu-19-*o*-OH,** only the isomer *E* is in full agreement with the obtained results and is sterically compatible.

The strength of the intramolecular hydrogen bonding for *o*-hydroxyazobenzocrowns seems to be dependent on macrocycle size cf. spectral data, [24,26], and is also influenced by the presence of *t*-butyl group in the *ortho* position to the OH moiety. The position of the OH proton signal in ^1^H NMR spectra (acetone-*d_6_*), as a dependent of macrocycle size, is shown in Figure 15 (inset).

### 2.5. Metal Cations Complexation in Solution

**Figure 16:**
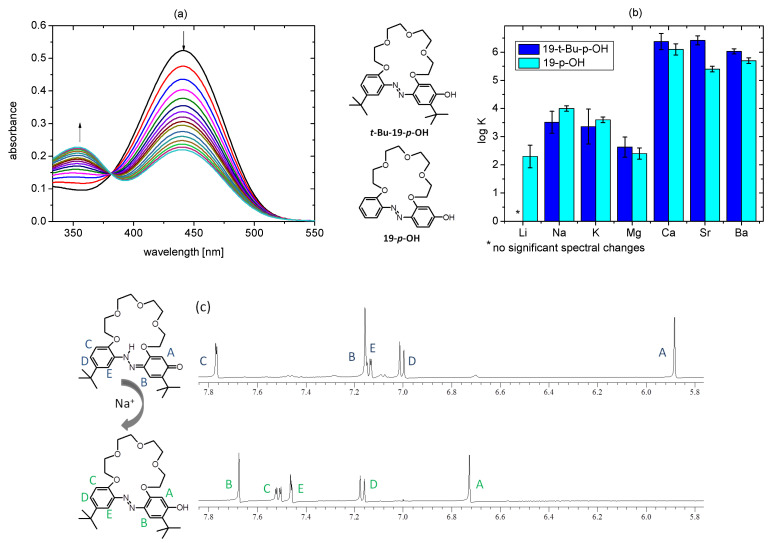
(**a**) Changes in UV-Vis spectrum upon titration of ***t*-Bu-19-*p*-OH** (1.56 × 10^−5^ M) with sodium perchlorate (0–1.6 × 10^−3^ M); (**b**) comparison of the stability constants values (log K) of 1:1 complexes of 19-membered *p*-hydroxyazobenzocrowns (shown here as azo–phenol tautomers): ***t*-Bu-19-*p*-OH** and **19-*p*-OH [26]** in acetonitrile; (**c**) partial (aromatic proton signals range) ^1^H NMR spectrum of ***t*-Bu-19-*p*-OH** (top; 5.7 × 10^−3^ M), registered in the presence of 10-fold excess of sodium perchlorate bottom (acetonitrile-*d_3_*).

**Figure 17:**
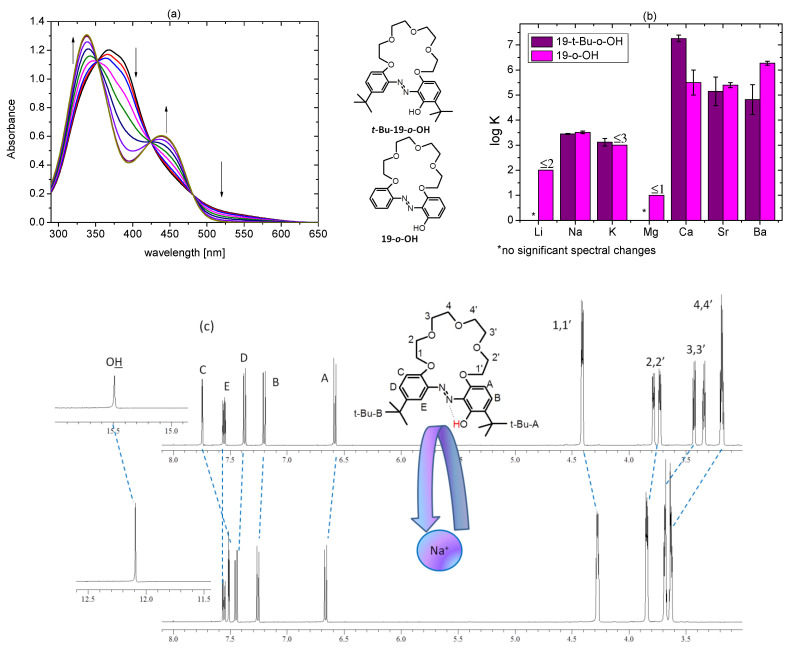
(**a**) Changes in UV-Vis spectra upon titration of **19-*t*-Bu-*o*-OH** (7.33 × 10^−5^ M) with calcium perchlorate (0–1.19 × 10^−4^ M) in acetonitrile; (**b**) comparison of the stability constants values (log K) of 1:1 complexes of 19-membered *o*-hydroxyazobenzocrowns—***t*-Bu-19-*o*-OH** and **19-*o*-OH [26]** in acetonitrile (**c**) partial (oligoether and aromatic proton signals range) ^1^H NMR spectrum of ***t*-Bu-19-*o*-OH** (top; 6.3 × 10^−3^ M) registered in the presence of a 10-fold excess of sodium perchlorate (acetonitrile-*d_3_*).

Alkali (lithium, sodium and potassium) and alkaline earth (magnesium, calcium, strontium and barium) metal cation complexation studies for ***t*-Bu-19-*p*-OH** and ***t*-Bu-19-*o*-OH** were carried out in acetonitrile by UV-Vis absorption and emission spectroscopies. The metal cation binding affinity were compared with parent macrocycle azobenzocrown ***t*-Bu-19-Azo** and its azoxy derivative ***t*-Bu-19-Azo-O** [26,34]. Notably, the latter macrocycles have not been investigated as chromoionophores in solution until now. On the other hand, the effect of the bulky *t*-butyl substituents in benzene rings on cation binding in acetonitrile has been analyzed in regard to the previously described 19-membered *para* and *ortho* hydroxyazobenzocrowns, **19-*p*-OH** and **19-*o*-OH,** respectively [26]. Formulas of the last are recalled in Figure 16 and Figure 17.

***t*-Bu-19-*p*-OH,** in acetonitrile form, complexes with the above-mentioned metal perchlorates, indicated by more or less pronounced changes observed in UV-Vis absorption spectra (Figure S6). Only lithium perchlorate was found to have no significant effect on the absorption spectrum of ***t*-Bu-19-*p*-OH**. On the basis of the previously investigated macrocycles of this type, it can be concluded that in this case the metal cation complexation is connected with the shift of tautomeric equilibrium towards the azo–phenol form. This is manifested in UV-Vis absorption spectra by the decrease in the absorption band at ~450 nm, parallel to the increase in the intensity of a band at ~360 nm. This spectral change corresponds to the color change of the solution from yellow to almost colorless (Figure S7). As an example, UV-Vis titration traces for sodium perchlorate in acetonitrile are shown in Figure 16a. ^1^H NMR spectrum (aromatic proton signals range) of ***t*-Bu-19-*p*-OH** registered in the presence of 10-fold excess of sodium perchlorate in acetonitrile-*d_3_* is presented in Figure 16c. The most characteristic for quinone–hydrazone form in ^1^H NMR spectrum signal: singlet at 5.88 ppm, registered in the presence of sodium, perchlorate is no longer present and the set of the of aromatic proton signals corresponds to the azo–phenol tautomer. Upon sodium complexation, the spectral pattern of the proton signals of the oligoether moiety is also changed. This confirms the expected complex formation with the engagement of oxygen atoms in sodium cation coordination. Full-range ^1^H NMR spectrum of ***t*-Bu-19-*p*-OH,** registered in the presence of a 10-fold excess of sodium perchlorate, is shown in Figure S8.

The stability constant values (log K) calculated from titration experiments are higher for 19-membered crown **19-*p*-OH** than for macrocycle with bulky substituents ***t*-Bu-19-*p*-OH** when forming complexes with alkali metal cations. The latter forms stronger complexes with alkaline earth metal cations in acetonitrile (Figure 16b, Table S3).

UV-Vis titration trace of ***t*-Bu-19-*o*-OH** with metal perchlorates in acetonitrile in general characterizes more significant changes for alkaline earth metal cations than for alkali metal ones (Figure S9). No changes in the presence of lithium and magnesium perchlorates were observed under titration conditions. Spectral changes in the presence of metal perchlorates are exemplified with calcium perchlorate titration (Figure 17a), showing the increase in the band intensity in a region of 450 nm, along with the disappearance of the band over 500 nm. The main absorption band is shifted from 370 to 336 nm. These spectral changes explain why metal cation binding by ***t*-Bu-19-*o*-OH** in acetonitrile is not connected with spectacular color changes (Figure S10).

For 19-membered *o*-hydroxyazobenzocrowns, the effect of the presence of *t*-butyl substituents is noticeable as the reversion of the trend of values of stability constant values with alkaline earth metal perchlorates (Figure 17b, Table S3). In the case of ***t*-Bu-19-*o*-OH,** it is Ca >> Sr > Ba, whereas for **19-*o*-OH** it is Ca~Sr < Ba [26].

**Figure 18:**
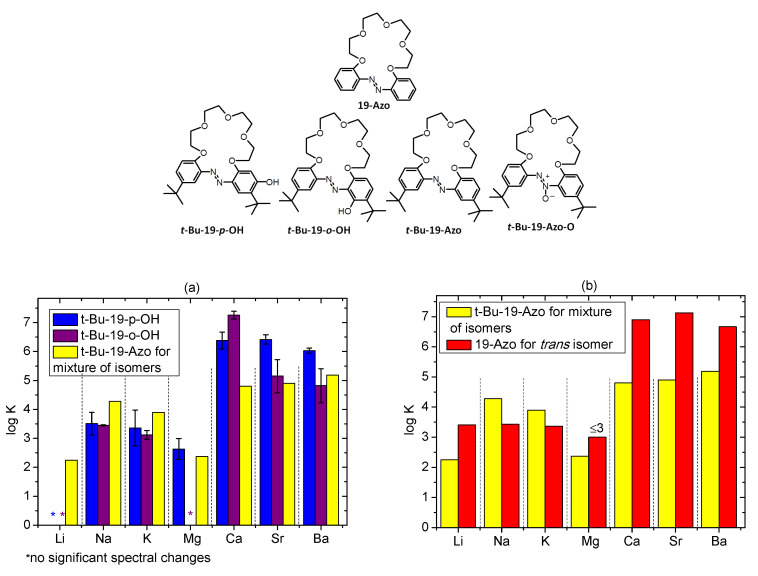

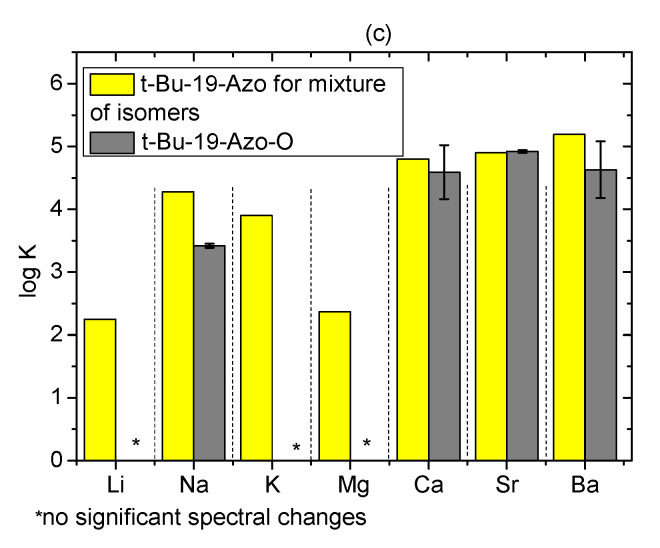
Comparison of the stability constant values (logK) of complexes of *t*-Bu-19-membered crowns: (**a**) ***t*-Bu-19-*p*-OH**, ***t*-Bu-19-*o*-OH** and ***t*-Bu-19-Azo**; (**b**) ***t*-Bu-19-Azo** and **19-Azo** [14,15]; (**c**) ***t*-Bu-19-Azo** and ***t*-Bu-19-Azo-O** with metal perchlorates in acetonitrile.

Metal cation complexation by ***t*-Bu-19-*o*-OH,** as might be expected, occurs with the engagement of oxygen atoms of oligoether linkage. This is confirmed by the change of the spectral pattern of proton signals of oligoether moiety exemplified by ^1^H NMR spectrum of ***t*-Bu-19-*o*-OH,** registered in the presence of a 10-fold excess of sodium perchlorate (Figure 18c). Worth noting is the shift of the OH proton signal from 15.5 to 12.2 ppm. It highlights the weakening of the strength of the hydrogen bonding upon metal cation complexation, which, in turn, might suggest the expected participation of the azo moiety nitrogen atom in the metal cation complex formation. Such modes of metal cation complex formation were confirmed in solid state for a series of azobenzocrowns [16,17,18,19]. Having this in mind, and similar to some extent, this model might be assumed in solution for *o*-hydroxyazobenzocrown, investigated here.

At this point, it seems worthwhile to compare the metal cation binding properties of the newly obtained 19-membered hydroxyazobenzocrowns bearing *t*-butyl moieties with macrocycles of the same ring size, namely ***t*-Bu-19-Azo**, **19-Azo** and **19-Azo-O**. Among the last mentioned three crowns, metal cation complexation studies in solution were carried out only for **19-Azo** so far [14,15]. As can be seen from the comparison of the stability constant values (log K) of 1:1 complexes (Figure 18a), hydroxyazobenzocrowns in acetonitrile form with alkaline earth metal perchlorates (besides magnesium) complexes of higher values of stability constants than azobenzocrown ***t*-Bu-19-Azo**. This is a similar trend as that observed for the previously investigated **19-*o*-OH** [26], with the exception that, among them, the highest value of binding constant was found for the calcium complex of ***t*-Bu-19-*o*-OH**. This may be considered as a promising calcium/magnesium selectivity for the possible application of this crown as a calcium receptor. Alkali metal cations are more strongly bound by ***t*-Bu-19-Azo** (Figure 18a).

Discussing metal cations complexation by ***t*-Bu-19-Azo,** it must clearly be underlined here that the process was investigated in acetonitrile using a target compound as a mixture of *Z* and *E* isomers. The ratio of isomers was estimated on the basis of ^1^H NMR spectroscopy as 35% of *E* and 65% of *Z* isomer (in DMSO, the ratio of *E* and *Z* isomers is 45% and 55%, respectively). ^1^H NMR spectra of ***t*-Bu-19-Azo** in acetonitrile-*d_3_* and DMSO-*d_6_* are included in the Supplementary Materials in Figure S10. Spectral changes upon UV-Vis titration of ***t*-Bu-19-Azo** with metal perchlorates are better pronounced for alkaline earth than for alkali metal cations (Figure S11). The obtained stability constant values (log K) of 1:1 complexes are, in general, lower than for **19-Azo** (Figure 18b), for which the given binding constants [14,15] refer to complexation studies carried out for the *E* isomer. *Z* isomers of azobenzocrowns were found to form weaker complexes with metal cations in solution, especially at lower salt concentrations. Thus, the obtained binding constants given in Table S3 must be treated as estimations only. Metal cation complexation for ***t*-Bu-19-Azo** was also investigated by ^1^H NMR spectroscopy at higher concentrations of crown and metal perchlorate. The presence of metal perchlorate, as might be expected, affects the ratio of isomers as a result of complex formation. Experiments in which sodium perchlorate was used confirm the metal cation complex formation occurring mainly by the *E* isomer, this is shown in the ^1^H NMR spectra in Figure S12 and Table 3.

The comparison of the complexation properties of ***t*-Bu-19-Azo-O** in regard of spectral changes upon UV-Vis titration with metal perchlorates (Figure S13) points out higher affinity of this crown towards alkaline earth metal cations: calcium, strontium and barium. Among the investigated alkali metal perchlorates, only sodium salt presence caused noticeable spectral changes. The estimated values of stability constants from UV-Vis measurements (log K) of 1:1 complexes of ***t*-Bu-19-Azo-O** with metal perchlorates are shown in Figure 18c and collected in Table S3. Binding constant for sodium complex is lower than for 19-membered azobenzocrown, whereas for alkaline earth metal perchlorates stability constants can be treated as more or less comparable.

Preliminary UV-Vis studies on alkali and alkaline earth metal cations complexation by ***t*-Bu-20-ester** carried out in acetonitrile showed no or minor spectral changes in the presence of the above-mentioned metal perchlorates, which is exemplified in Figure S14. More detailed complexation studies including this compound, as well as ***t*-Bu-19-al** and ***t*-Bu-17-*p*-OH,** are currently under detailed investigation in our lab.

## 3. Materials and Methods

### 3.1. General Information

Unless otherwise stated, materials and solvents were of analytical reagent grade obtained from commercial suppliers and were used without further purification. UV-Vis and spectrofluorometric measurements were carried out in commercial solvents of the highest available purity: DMSO (spectroscopic-grade, POCh, Gliwice, Poland), dichloromethane (LiChrosolv and SupraSolv, MERCK, Darmstadt, Germany), methanol (HPLC grade, POCh, Gliwice, Poland) and acetonitrile (LiChrosolv, MERCK, Germany). For measurements performed in mixed, water-containing systems, deionized water (conductivity <1 μS∙cm^−1^, Hydrolab, Straszyn, Poland) was used.

Photochemical reactions were carried out in quartz flasks using photoreactor prototype designed by Dariusz Wysiecki MSc., Eng. and constructed in cooperation with the Enviklim Company (Gdańsk, Poland). The reactor is equipped with 3 UVA diode arrays (2 × UV-D6565-4LED, 40 W and 1 × UV-D6565-15LED, 150 W, λ = 365–370 nm). ^1^H and ^13^C NMR spectra were recorded on a Varian INOVA 500 spectrometer at 500 and at 125 MHz, respectively. Chemical shifts are reported in δ (ppm) units. FTIR spectra (film) were taken on Nicolet iS10 apparatus. High resolution mass spectra (HRMS) were taken on a SYNAPT G2-S HDMS (waters) spectrometer with electrospray ionization source (ESI) and a TOF mass analyzer. UV-Vis measurements were carried out with the use of an UNICAM UV 300 series spectrometer. Fluorescence spectra were recorded on a luminescence spectrometer (AMINCO Bowman Series 2 spectrofluorimeter) using the flash xenon lamp. The bandpass of excitation and emission monochromators was 16 nm. UV-Vis and fluorescence measurements were carried out in 1 cm quartz cuvettes.

### 3.2. Preparation

The reaction progress and purity of products were monitored by TLC using aluminum sheets covered with silica gel 60 F_254_ (Merck). UV light (254 nm) was used as the detection method. Reaction mixtures were separated using a classical column (silica gel 60, 0.063–0.200 mm, Merck) or preparative thin layer chromatography (PLC plates, silica gel 60F_254_, 1 mm, 20 × 20 cm size, Merck). Reagent grade solvents were used.

#### 3.2.1. Preparation of Substrate for Rearrangements–di-*tert*-Butyl-19-Azoxybenzocrown (***t*-Bu-19-Azo-O**)

This compound was prepared first time by Skwierawska and others [34].

***t*-Bu-19-Azo-O** was prepared by us analogously to the 19-membered azoxybenzocrown [26]. Starting from 11.0 g of 1,11-bis(2-nitro-4-*tert*-butylphenoxy)-3,6,9-trioxaundecane (podand) 3.8 g (38%) of *ter*t-butyl-19-azoxybenzocrown (***t*-Bu-19-Azo-O**) as light-yellow oil was obtained. The substance was stored in the dark.

^1^H NMR (500 MHz, chloroform-*d*): 1.35 (s, 9H), 1.36 (s, 9H), 3.62–3.66 (m, 4H), 3.66–3.70 (m, 4H), 3.90–3.93 (m, 4H), 4.26–4.29 (m, 4H), 6.99 (d, J = 8.8 Hz, 1H), 7.05 (d, J = 8.3 Hz, 1H), 7.34 (dd, J1 = 8.8 Hz, J2 = 2.4 Hz, 1H), 7.44 (dd, J1 = 8.8 Hz, J2 = 2.4 Hz, 1H), 7.66 (d, J = 2.6 Hz, 1H), 8.02 (d, J = 2.5 Hz, 1H). ^1^H NMR (500 MHz, acetone-*d_6_*): 1.35 (s, 9H), 1.37 (s, 9H), 3.54–3.58 (m, 2H), 3.60-3.64 (m, 2H), 3.85 (t, J = 5 Hz, 2H), 3.87 (t, J = 5 Hz, 2H), 4.22 (t, J = 5 Hz, 2H), 4.28 (t, J = 5.3 Hz, 2H), 7.10 (d, J = 8.8 Hz, 1H); 7.23 (d, J = 8.7 Hz, 1H), 7.39 (dd, J1 = 8.7 Hz, J2 = 2.5 Hz, 1H), 7.56 (dd, J1 = 8.8 Hz, J2 = 2.5 Hz, 1H), 7.69 (d, J = 2.4 Hz, 1H), 8.08 (d, J = 2.4 Hz, 1H). ^13^C NMR (125 MHz, acetone-*d_6_*): 30.7, 30.9, 33.97, 34.03, 68.48, 69.18, 69.22, 69.35, 70.44, 70.48, 70.82, 71.04, 113.3, 114.9, 119.9, 121.7, 126.0, 127.7, 133.9, 140.1, 142.7, 143.7, 148.9, 150.8. FT-IR (film), ν = 599, 628, 736, 834, 886, 946, 1055, 1082, 1126, 1169, 1202, 1266, 1365, 1395, 1460, 1509, 1616, 2871, 2961, 3048, cm^−1^. UV/Vis (acetonitrile): λ_1_ (ε_1_) 305 (4.94 × 10^3^), λ_2_ (ε_2_) 341 (4.75 × 10^3^). TLC: Rf (CHCl_3_:MeOH, 30:1, *v*/*v*): 0.39; (DCM:acetone, 10:1, *v*/*v*): 0.33.

#### 3.2.2. Photochemical Rearrangement of ***t*-Bu-19-Azo-O**

***t*-Bu-19-Azo-O** (typically 70 mg) in a quartz Erlenmeyer flask, dissolved in the given solvent (125 mL per 70 mg of substrate, solvents with a boiling point above 80 °C were used) was irradiated with UV light for 80 min in the photoreactor. Within the timescale of the experiment, the temperature of the solution was increasing up to ~70 °C (Table 1). As a result of the reaction progress the color of the solution changed from light yellow to red-orange or red. After that time, the mixture was quantitatively transferred to a round bottom flask to evaporate the solvent under reduced pressure. After dissolution in dichloromethane, the products of the photochemical rearrangement were isolated by preparative thin-layer chromatography with a chloroform:methanol mixture (30:1, *v*/*v*) or dichloromethane:acetone (10:1 *v*/*v*) as the mobile phase (alternatively, column chromatography was pre-applied). The separated products were eluted from silica gel with a mixture of chloroform–acetone–methanol. After filtration and solvents evaporation, the residue was dissolved in dichloromethane and filtrated once again. Then, the solvent was evaporated, and the residue was weighed. Yields of colored photochemical rearrangement products are collected in Table 1. ***t*-Bu-20-ester** was crystallized from methanol (red crystals, mp: 117–118 °C), ***t*-Bu-19-*p*-OH** was crystallized from 2-propanol/hexane (orange crystals, mp: 184–186 °C), ***t*-Bu-19-*o*-OH** was crystallized from DMSO and crystals were washed with hexane (orange-brown crystals, mp: 83–84 °C), ***t*-Bu-19-al** was crystallized from 2-propanol (dark beige precipitate, mp: 131–133 °C).

Among products the of photorearrangement process, carried out in toluene compound, ***t*-Bu-17-*p*-OH** was isolated and identified: colorless compound crystallizes from *n*-hexane (mp: 138–140 °C).

#### 3.2.3. Thermochemical Rearrangement of ***t*-Bu-19-Azo-O**

The azoxycrown was heated in a glass flask protected from light, both in solution and in the form of an oily film—without solvent. The substance was heated under conditions (temperature, solvent) similar to those used in photochemistry and at a significantly higher temperature.

The five basic variants were the following: (a) heating the azoxy compound (11 mg) in toluene solution (20 mL) at 80 °C for 2 h (conditions similar to those for photochemistry); (b) heating the azoxy compound (20 mg) in xylene solution (10 mL) at 130 °C for 3 h; (c) heating the azoxy compound (20 mg) in DMF solution (10 mL) at 150 °C for 3 h; (d) heating the azoxy compound (20 mg) without solvent at 160 °C (silicone oil bath temperature) for 1 h; (e) heating the azoxy compound (20 mg) without solvent at 160 °C (silicone oil bath temperature) for 3 h.

Solvents were evaporated, the residues were analyzed by TLC. It was found that in variant (a) no reaction took place, and in variants (b) and (c), the solutions turned red and significant amounts of ***t*-Bu-20-ester** were formed. Additionally, in the case of variant (d), the presence of ***t*-Bu-20-ester** was found and in the case of variant (e), and the vast majority of the substrate was rearranged into ***t*-Bu-20-ester**. Products obtained in variants (b)–(e) were purified by column chromatography (DCM-acetone 20:1) to give the ***t*-Bu-20-ester** with the following yields: (b) 23, (c) 68, (d) 35 and (e) 82%, respectively.

#### 3.2.4. Spectral Characterization of the Products of Photochemical and Thermochemical Rearrangements of ***t*-Bu-19-Azo-O**

***t*-Bu-20-ester**

^1^H NMR (500 MHz, DMSO-*d_6_*), δ = 1.22 (s, 3H), 1.30 (s, 3H), 3.52–3.60 (m, 6H), 3.66 (t, J = 5.0 Hz, 2H), 3.81 (t, J = 5.2 Hz, 2H), 3.93 (t, J = 4.6 Hz, 2H), 4.21 (t, J = 4.6 Hz, 2H), 4.52 (t, J = 5.2 Hz, 2H), 6.56 (d, J = 2.5 Hz, 1H), 7.03 (d, J = 8.5 Hz, 1H), 7.10 (dd, J1 = 8.5, J2 = 2.2 Hz, 1H), 7.65 (d, J = 2.2 Hz, 1H), 7.69 (d, J = 2.5 Hz, 1H), 13.93 (s, 1H). ^13^C NMR (125 MHz, DMSO-*d_6_*), δ = 30.4, 31.8, 32.1, 34.6, 64.3, 69.0, 69.2, 69.5, 70.5, 70.8, 70.9, 71.0, 111.1, 112.8, 114.4, 121.6, 126.2, 131.5, 142.9, 143.4, 144.4, 145.3, 150.9, 165.8. FT-IR (film), ν = 759, 809, 829, 950, 1021, 1073, 1092, 1135, 1249, 1279, 1328, 1341, 1362, 1432, 1455, 1511, 1557, 1591, 1668, 2867, 2958, 3479 cm^−1^. HRMS (ESI): calcd for C_28_H_40_N_2_O_6_ [M+Na]^+^: 523.2784, found 523.2787. UV/Vis (acetonitrile): λ_max_ (ε_max_) 473 (9.55 × 10^3^). TLC: Rf (CHCl_3_:methanol, 30:1, *v*/*v*): 0.70; (DCM:acetone, 10:1): 0.65.

***t*-Bu-19-al**

^1^H NMR (500 MHz, acetone-*d_6_*), δ = 1.35 (s, 9H), 1.40 (s, 9H), 3.68-3.74 (m, 4H), 3.76–3.78 (m, 2H), 4.03 (t, J = 5.0 Hz, 2H), 4.18 (t, J = 5.3 Hz, 2H), 4.35 (t, J = 5.0 Hz, 2H), 4.77 (t, J = 5.3 Hz, 2 H), 6.47 (s, 1H), 7.06 (d, J = 8.8 Hz, 1H), 7.12 (dd, J1 = 8.5, J2 = 2.4 Hz, 1H), 7.77 (d, J = 2.1 Hz, 1H), 10.10 (s, 1H), 11.25 (s, 0.5H). ^1^H NMR (500 MHz, DMSO-*d_6_*), δ = 1.28 (s, 9H), 1.32 (s, 9H), 3.60-3.70 (m, 8H), 3.89 (t, J = 5 Hz, 2H), 4.00 (t, J = 5 Hz, 2H), 4.27 (t, J = 4.8 Hz, 2H), 4.69 (t, J = 5 Hz), 6.36 (s, 1H), 7.07 (s, 2H), 7.62 (s, 1H), 9.97 (s, 1H), 11.10 (s, 1H). ^13^C NMR (125 MHz, acetone-*d_6_*), δ = 30.1, 30.9, 32.3, 34.0, 68.4, 69.3, 69.4, 70.4, 70.6, 71.2, 72.0, 75.9, 110.9, 112.0, 117.8, 119.2, 120.4, 131.5, 135.7, 144.3, 144.4, 152.9, 152.9, 183.5. ^13^C NMR (125 MHz, DMSO-*d*_6_), δ = 30.8, 31.6, 32.5, 34.5, 68.7, 69.3, 70.4, 70.6, 71.1, 71.7, 76.2, 110.9, 112.8, 117.4, 119.4, 121.1, 131.2, 135.7, 138.0, 144.3, 144.4, 153.4, 184.7. FT-IR (film), ν = 631, 756, 797, 880, 941, 1017, 1041, 1147, 1223, 1260, 1361, 1385, 1416, 1463, 1538, 1579, 1639, 2866, 2954, 3328 cm^−1^. HRMS (ESI^−^): calcd for C_28_H_40_N_2_O_6_ [M-H]^−^: 499.2808, found 499.2800. UV/Vis (acetonitrile): λ_max_ (ε_max_) 428 (1.10 × 10^4^). TLC: R_f_ (CHCl_3_:methanol, 30:1, *v*/*v*): 0.58; (DCM:acetone, 10:1): 0.39.

***t*-Bu-19-*o*-OH**

^1^H NMR (500 MHz, DMSO-*d_6_*) azo–phenol tautomer, δ = 1.32 (s, 9H), 1.39 (s, 9H), 3.07–3.12 (m, 4H), 3.25 (t, J = 4.9 Hz, 2H), 3.37 (t, J = 4.8 Hz, 2H), 3.62-3.64 (m, 2H), 3.69–3.71 (m, 2H), 4.32–4.36 (m, 4H), 6.58 (d, J = 8.6 Hz, 1H), 7.24 (d, J = 8.5 Hz, 1H), 7.28 (d, J = 8.5 Hz, 1H), 7.50 (J1 = 8.8 Hz, J2 = 2.5 Hz, 1H), 7.63 (d, J = 2.5 Hz, 1H), 15.26 (s, 1H). ^13^C NMR (125 MHz, DMSO-*d_6_*): 29.8, 31.6, 34.61, 34.64, 69.77, 69.90, 70.03, 70.40, 70.55, 71.73, 71.84, 105.9, 114.6, 118.3, 129.0, 130.5, 131.0, 131.8, 140.8, 144.9, 153.3, 154,7, 157.8. FT-IR (film), ν = 755, 821, 892, 940, 1039, 1107, 1138, 1201, 1246, 1296, 1362, 1391, 1424, 1446, 1500, 1594, 2868, 2957, 3446 cm^−1^. HRMS (ESI): calcd for C_28_H_40_N_2_O_6_ [M+Na]^+^: 523.2784, found 523.2785. UV/Vis (acetonitrile): λ_max_ (ε_max_) 374 (1.57 × 10^4^). TLC: R_f_ (CHCl_3_:methanol, 30:1, *v*/*v*): 0.42; (DCM:acetone, 10:1): 0.35.

***t*-Bu-19-*p*-OH**

^1^H NMR (500 MHz, DMSO-*d_6_*), 80% of the phenolic form and 20% of the quinone form: δ = 1.27 (s, 9H), 1.30 (s, 1.8H), 1.33 (s, 7.2H), 3.38–3.48 (m, 4.14H), 3.61–3.72 (m, 6H), 3.89 (t, 0.4H, J = 4.7 Hz), 3.97 (t, 0.4H, J = 5.2 Hz), 4.19–4.24 (m, 4.2 H), 4.27 (0.4 H, t, J = 4.7 Hz), 4.32 (0.4H, J = 5.3 Hz), 5.91 (0.2H, s), 6.59 (1H, s), 6.90 (0.8H, d, J = 2.4 Hz), 7.02–7.08 (2H, m), 7.37 (0.8H, dd, J1 = 8.5 Hz, J2 = 2.6 Hz), 7.63 (0.2H, d, J = 2 Hz), 10.16 (0.8H, s), 11.67 (0.2H, s). ^13^C NMR (125 MHz, DMSO-*d_6_*), δ = 29.6, 29.9, 31.7, 31.8, 34.3, 34.6, 34.6, 68.2, 68.5, 68.5, 68.6, 68.7, 69.1, 69.2, 70.4, 70.4, 71.0, 71.4, 102.1, 107.7, 111.0, 112.,6, 113.8, 116.3, 117.2, 121.4, 126.8, 128.2, 129.3, 130.9, 133.6, 136.9, 142.9, 143.3, 144.5, 145.0, 150.6, 154.3, 158.0, 160.1. ^1^H NMR (500 MHz, acetone-*d_6_*), 100% of the quinone form: δ = 1.33 (s, 9H), 1.35 (s, 9H), 3.67–3.76 (m, 8H), 4.02 (t, 2H, J = 5.0 Hz), 4.13 (t, 2H, J = 5.1 Hz), 4.35 (t, 2H, J = 5.0 Hz), 4.95 (t, 2H, J = 5.6 Hz), 5.88 (1H, s), 7.05 (1H, d, J = 8.6 Hz), 7.09 (1H, s), 7.13 (1H, dd, J1 = 8.5 Hz, J2 = 2.5 Hz), 7.77 (1H, d, J = 2.1 Hz), 11.74 (1H, s). ^13^C NMR (125 MHz, acetone-*d_6_*): 29.4, 30.9, 34.0, 34.1, 68.34, 68.43, 68.61, 69.32, 70.51, 70.64, 71.19, 71.73, 107.3, 110.95, 110.99, 111.82, 129.3, 131.15, 131.27, 133.42, 142.85, 144.47, 144.50, 158.0, 184.7. FT-IR (film), ν = 756, 804, 847, 878, 905. 941, 983, 1017, 1055, 1077, 1135, 1173, 1187, 1200, 1224, 1270, 1346, 1361, 1388, 1423, 1441, 1453, 1461, 1501, 1521, 1526, 1563, 1593, 1625, 2868, 2907, 2955, 3315 cm^−1^. HRMS (ESI): calcd for C_28_H_40_N_2_O_6_ [M+Na]^+^: 523.2784, found 523.2788. HRMS (ESI): calcd for C_28_H_40_N_2_O_6_ [M+H]^+^: 501.2965, found 501.2972. UV/Vis (acetonitrile): λ_max_ (ε_max_) 442 (3.19 × 10^4^). TLC: R_f_ (CHCl_3_:methanol, 30:1, *v*/*v*): 0.35; (DCM:acetone, 10:1): 0.12.

***t*-Bu-17-*p*-OH**

^1^H NMR (500 MHz, acetone-*d_6_*): 1.33 (9H, s), 1.42 (9H, s), 3.51–3.65 (8H, m), 3.67–3.74 (2H, m), 3.76–3.83 (2H, m), 3.90–3.95 (1H, m), 3.96–4.07 (1H, m), 4.06–4.11 (1H, m), 4.13–4.18 (1H, m), 6.60 (1H, s), 6.95 (1H, d, J = 8.5 Hz), 7.00 (1H, s), 7.19 (1H, d, J = 2.5 Hz), 7.29 (1H, dd, J1 = 8.5, J2 = 2.5 Hz), 8.4 (broad signal). ^13^C NMR (125 MHz, DMSO-*d_6_*): δ = 30.1, 31.9, 34.0, 34.2, 67.4, 67.6, 69.1, 69.3, 70.3, 70.3, 70.6, 70.7, 101.3, 111.9, 118.6, 124.8, 127.2, 128.4, 128.7, 129.3, 142.4, 154.5, 155.0, 156.2. FT-IR (film), ν = 828, 892, 947, 1086, 1127, 1188, 1219, 1247, 1291, 1311, 1361, 1403, 1463, 1498, 1518, 1558, 1605, 1733, 2856, 2926, 3281 cm^−1^. HRMS (ESI): calcd for C_28_H_40_O_6_ [M+Na]^+^: 495.2723, found 495.2722. UV/Vis (acetonitrile): λ_1_ (ε_1_) 206 (6.19 × 10^4^), λ_2_ (ε_1_) 289 (1.17 × 10^4^). TLC: R_f_ (CHCl_3_:methanol, 30:1, *v*/*v*): 0.14; (DCM:acetone, 10:1, *v*/*v*): 0.13. The structure of compound was confirmed by X-ray measurements.

### 3.3. X-ray Crystal Structure Determination

Diffraction intensity data for ***t*-Bu-19-*p*-OH**, ***t*-Bu-20-ester** and ***t*-Bu-17-*p*-OH** were collected on an IPDS 2T dual beam diffractometer (STOE and Cie GmbH, Darmstadt, Germany) at 120.0(2) K with MoKa radiation of a microfocus X-ray source (GeniX 3D Mo High Flux, Xenocs, Sassenage, 50 kV, 1.0 mA, and λ = 0.71069 Å). Data for ***t*-Bu-19-*o*-OH** were collected on the same diffractometer, but using a copper X-ray source (GeniX 3D Cu High Flux, Xenocs, Sassenage, 50 kV, 0.6 mA, and λ = 1.54186 Å). Investigated crystals were thermostated under a nitrogen stream at 120 K using the CryoStream-800 device (Oxford CryoSystems, Witney, UK) during the entire experiment.

Data collection and data reduction were controlled by using the X-Area 1.75 program (STOE, 2015). Due to low absorption coefficient no absorption correction was performed for Mo lamp data. Diffraction data from copper radiation were further processed by the program STOE *X-RED32*, for absorption correction by Gaussian integration, analogous to those described by Coppens [35]. The structures were solved using intrinsic phasing implemented in SHELXT and refined anisotropically using the program packages Olex2 [36] and SHELX-2015 [37,38]. Positions of the C–H hydrogen atoms were calculated geometrically taking into account isotropic temperature factors. All hydrocarbonic H atoms were refined as riding on their parent atoms with the usual restraints. NH and OH atoms were found in the Fourier electron density map and refined with N-H distance constrained to 0.85(2) Å and OH to 0.84(2) Å for all symmetry-independent molecules. Structures ***t*-Bu-19-*p*-OH**, ***t*-Bu-19-*o*-OH** and ***t*-Bu-17-*p*-OH** were refined with no special treatment. Structure of ***t*-Bu-20-ester** was refined as a two-component twin, with domain mass fractions of 0.765(4) and 0.235(4). Structure ***t*-Bu-17-*p*-OH** was refined with hydroxyl O6 group (and hydrogen H4), disordered as being attached alternatively to one ring only with site occupation factors 0.626(5)/0.374(5). Without this disorder, the thermal ellipsoid on O6 becomes unacceptably large. One *tert*-butyl group C25 was also modelled as disordered over two positions with occupancies 0.720(8)/0.280(8). Details are collected in Table 4.

### 3.4. Structure Determination of **t-Bu-20-ester**, **t-Bu-19-al** and **t-Bu-19-p-OH** in Solutions

NMR of ***t*-Bu-20-ester**

^1^H NMR spectrum was collected with standard parameters (at ambient temperature, 45° pulse length, 2 s acquisition time and delay time 1 s) in DMSO-*d_6_* solution.

^13^C NMR spectrum was collected with standard parameters (at ambient temperature, 45° pulse length, 1 s acquisition time and 1 s delay time) in DMSO-*d_6_* solution.

Two-dimensional NMR spectra were recorded at ambient temperature in DMSO-*d_6_* solution.

ROESY spectrum was collected in the phase-sensitive mode with a spectral width of 7997 Hz and a mix time of 300 ms in a 5278 × 640 matrix with 8 accumulations per increment, and was processed in a 2 K × 1 K complex points matrix.

gHSQCAD spectrum was acquired in the phase-sensitive mode with CRISIS-based multiplicity editing and ^1^J(CH) set to 146 Hz. The spectral windows for ^1^H and ^13^C of axes were 7997 Hz and 21,362 Hz, respectively. The data were collected in a 1458 × 192 matrix with 10 accumulations per increment and were processed in a 1 K × 1 K complex points matrix.

gHMBCAD spectrum was acquired in the phase-sensitive mode with ^n^J(CH) set to 8 Hz. The spectral windows for ^1^H and ^13^C axes were 7997 Hz and 22,618 Hz, respectively. The data were collected in a 3998 × 420 matrix with 72 accumulations per increment and were processed in mixed (absolute value and phase sensitive) mode in a 2 K × 1 K complex points matrix.

NMR of ***t*-Bu-19-al**

^1^H NMR spectrum was collected with standard parameters (at ambient temperature, 45° pulse length, 2 s acquisition time and delay time 1 s) in acetone-*d_6_* solution.

^13^C NMR spectrum was collected with standard parameters (at ambient temperature, 45° pulse length, 1 s acquisition time and 1 s delay time) in acetone-*d_6_* solution.

Two-dimensional NMR spectra were recorded at ambient temperature in acetone-*d_6_* solution.

ROESY spectrum was collected in the phase-sensitive mode with a spectral width of 5922 Hz and a mix time of 300 ms in a 3908 × 512 matrix with 8 accumulations per increment and was processed in a 2 K × 1 K complex points matrix.

gHSQCAD spectrum was acquired in the phase-sensitive mode with CRISIS-based multiplicity editing and ^1^J(CH) set to 146 Hz. The spectral windows for ^1^H and ^13^C of axes were 5922 Hz and 18,850 Hz, respectively. The data were collected in a 2132 × 230 matrix with 16 accumulations per increment and were processed in a 1 K × 1 K complex points matrix.

gHMBCAD spectrum was acquired in the phase-sensitive mode with ^n^J(CH) set to 8 Hz. The spectral windows for ^1^H and ^13^C axes were 5922 Hz and 22618 Hz, respectively. The data were collected in a 2962 × 400 matrix with 64 accumulations per increment and were processed in mixed (absolute value and phase sensitive) mode in a 2 K × 1 K complex points matrix.

NMR of ***t*-Bu-19-*p*-OH**

^1^H NMR spectrum was collected with standard parameters (at ambient temperature, 90° pulse length, 2 s acquisition time) in acetonitrile-*d*_3_ solution.

Two-dimensional NMR spectra were recorded at ambient temperature in acetonitrile-*d*_3_ solution.

ROESYAD spectrum was collected in phase-sensitive mode with a spectral width of 5992 Hz and a mix time of 300 ms in a 3956 × 400 matrix with 24 accumulations per increment and was processed in a 2 K × 1 K complex points matrix.

gHSQCAD spectrum was acquired in the phase-sensitive mode with CRISIS-based multiplicity ending and ^1^J(CH) set to 146 Hz. The spectral windows for ^1^H and ^13^C of axes were 5992 Hz and 22,618 Hz, respectively. The data were collected in a 2398 × 220 matrix with 8 accumulations per increment and were processed in a 1 K × 1 K complex points matrix.

gHMBCAD spectrum was acquired in the phase-sensitive mode with ^n^J(CH) set to 8 Hz. The spectral windows for ^1^H and ^13^C of axes were 5992 Hz and 24,502 Hz, respectively. The data were collected in a 2024 × 240 matrix with 24 accumulations per increment and were processed in mixed (absolute value and phase sensitive) mode 1 K × 1 K complex points matrix.

Two-dimensional NMR spectra were recorded at ambient temperature also in DMSO-*d_6_* solution.

ROESYAD spectrum was collected in phase-sensitive mode with a spectral width of 6198 Hz and a mix time of 300 ms in a 4092 × 450 matrix with 16 accumulations per increment and was processed in a 4 K × 1 K complex points matrix.

gHSQCAD spectrum was acquired in the phase-sensitive mode with CRISIS-based multiplicity ending and ^1^J(CH) set to 146 Hz. The spectral windows for ^1^H and ^13^C of axes were 6198 Hz and 22,618 Hz, respectively. The data were collected in a 2480 × 200 matrix with 16 accumulations per increment and were processed in a 2 K × 1 K complex points matrix.

gHMBCAD spectrum was acquired in the phase-sensitive mode with ^n^J(CH) set to 8 Hz. The spectral windows for ^1^H and ^13^C of axes were 6198 Hz and 24,502 Hz, respectively. The data were collected in a 2480 × 200 matrix with 16 accumulations per increment and were processed in mixed (absolute value and phase sensitive) mode 2 K × 1 K complex points matrix.

### 3.5. Studies of Crown–Metal Cations Interactions

For metal cation complexation, lithium perchlorate (99.9%, Sigma Aldrich, Milwaukee, WI, USA), sodium perchlorate monohydrate (>99%, Fluka, Buchs, Switzerland), potassium perchlorate (>99%, Sigma Aldrich, Milwaukee, WI, USA), magnesium perchlorate (≤100%, Sigma Aldrich, Steinhaim, Germany), calcium perchlorate tetrahydrate (99%, Sigma Aldrich, Milwaukee, WI, USA), strontium perchlorate trihydrate (≤100%, Sigma Aldrich, Steinhaim, Germany), barium perchlorate (97%, Sigma Aldrich, Steinhaim, Germany. For acid-base properties studies *p*-toluenesulfonic acid monohydrate (pure, POCH, Gliwice, Poland), tetra-*n*-butylammonium hydroxide 30-hydrate (98%, Sigma-Aldrich, Steinhaim, Germany) were used. Complexation studies were performed by spectrophotometric titration of the crown solution in acetonitrile with the respective metal perchlorate (for metal cations). The stock solutions of azobenzocrowns (~10^−4^ M) and metal perchlorates were prepared by weighing their respective quantities and dissolving in acetonitrile in volumetric flasks. For titrations, azobenzocrown solutions of 2.3 mL starting volume in the cuvette were used. The stability constant values from UV-Vis experiments were calculated with the use of the OPIUM [39] program.

## 4. Conclusions

The photochemical rearrangement of azoxybenzocrowns is an interesting and multidirectional process and seems to be a relatively efficient method for obtaining not only hydroxy–azo compounds, but also other macrocyclic compounds with a hydrazone group in macroring. The photochemical procedure allows the preparation of various compounds with yields dependent on the process conditions. A novelty of this paper is the study of the photochemical reaction with 19-membered azoxybenzocrowns containing substituents on benzene rings, in this case, *tert*-butyl substituents, which block the *para* position with respect to the oligoether chain. Another important novelty is in the isolation, besides that of the expected *tert*-butyl-19-*ortho*- and *tert*-butyl-19-*para* hydroxyazobenzocrowns, also that of the macrocyclic compounds of new types—the macrocycles with the characteristic element of a 5-membered ring, resulting from the contraction of the benzene ring. Two such compounds were isolated and identified. The first one is aldehyde (a compound of a somewhat similar structure was identified as the product of a photo-rearrangement of unsubstituted 19-membered azoxybenzocrown), the second one is intramolecular ester derivative. They are hydrazone derivatives with a proton directed towards the interior of the macrocycle. These products differ in structure from products which were isolated from rearrangements of the unsubstituted 19-azoxybenzocrown [26]. It was also found that thermal rearrangement of *t*-Bu 19-membered azoxybenzocrown, carried out without the access of UV light, practically, leads to one product, namely ***t*-Bu-20-ester**. The highest yield, 80%, was obtained by heating the substrate in the absence of a solvent at 160 °C.

The presented methods of the preparation of macrocycles of more or less sophisticated structures seem to be greener and more convenient approaches than traditional synthetic routes, which are usually multistep reactions and need significant amounts of solvents and reagents—used just on the synthesis stage, as well as during isolation.

***t*-Bu-19-*p*-OH,** opposite to **19-*p*-OH,** predominantly exists in quinone–hydrazone form in most solvents, which can be connected with the effect of the bulky substituent in the *ortho* position to the hydroxyl group. Results obtained for a series of *o*-hydroxy macrocycles of 13-, 16- and 19-membered crowns show that intramolecular hydrogen bond is stronger in macrocycles of larger size and additionally is affected by the presence of substituents in the *ortho* position to the OH moiety.

The 19-membered hydroxyazobenzocrowns formed stronger complexes (1:1) with calcium, strontium and barium than with alkali metal perchlorates in acetonitrile. In the case of ***t*-Bu-19-*p*-OH**, which exists in the quinone–hydrazone form, metal cation complexation shifts the tautomeric equilibrium towards the azo–phenol form. ***t*-Bu-19-*o*-OH** shows an interesting selectivity towards calcium, which warrants further study. The detailed studies of metal cation complexation by the processes described in this manuscript for macrocycles will be the topic of a future paper, in which efforts will be focused on the possible analytical applications of the mentioned systems.

## Figures and Tables

**Table 1:** Yields of the four, colored, macrocyclic products obtained in photochemical rearrangements of ***t*-Bu-19-Azo-O** *.

Input	Solvent	Maximum Solution Temperature [°C]	Yield [%]				The Total Yield of Four Macrocyclic, Colored Products
			*t*-Bu-20-ester	*t*-Bu-19-al	*t*-Bu-19-*o*-OH	*t*-Bu-19-*p*-OH	
A.	Toluene	74	3.5	4.0	45.4	40.5	93.4
B.	Xylene	68	4.3	8.0	45.0	40.0	97.3
C.	DMF	73	0.6	-	53.8	16.9	71.3
D.	2-propanol	64	7.1	5.7	10.0	64.3	87.1
E.	*s*-butanol	68	8.0	7.0	5.0	70.0	90.0
F.	*n*-butanol	75	2.5	2.3	11.3	48.8	64.9
G.	2-propanol/ acetic acid (0.2 mL)	73	-	-	73.0	13.0	86.0
H.	toluene/ acetic acid (0.2 mL)	70	-	-	66.7	6.2	72.9

* Conditions: 70 mg of azoxycrown in 125 mL of solvent, light 365–370 nm, power approx. 140 W, reaction time 80 min.

**Table 2:** Hydrogen bond geometry (Å, °).

*D*—H···*A*	*D*—H	H···*A*	*D*···*A*	*D*—H···*A*
***t*** **-Bu-19-*o*-OH**				
O6—H6···N2	0.93 (2)	1.63 (2)	2.500 (2)	155 (4)
O12—H12···N4	0.95 (2)	1.66 (3)	2.526 (2)	150 (4)
***t*** **-Bu-19-*p*-OH**				
N2—H2···O1	0.89 (2)	2.04 (5)	2.730 (6)	134 (6)
N4—H4···O7	0.89 (2)	2.04 (5)	2.695 (6)	130 (5)
***t*** **-Bu-20-ester**				
N2—H2···O6	0.89 (2)	1.85 (2)	2.716 (3)	164 (4)
***t*-Bu-17-*p*-OH**				
O6—H6···O2 ^i^	0.84 (2)	1.89 (4)	2.657 (7)	150 (6)

Symmetry code: (i) −*x*, −*y* + 1, −*z* + 1.

**Table 3:** The ratio (given as % of content) of *Z* and *E* isomers of ***t*-Bu-19-Azo** in acetonitrile solution and its change upon metal complex formation (estimated on the basis of ^1^H NMR experiment in acetonitrile-*d_3_*).

Isomer	“Free” *t*-Bu-19-Azo	Equimolar Amount of NaClO_4_	10-Fold Excess of NaClO_4_
*Z*	65	63	31
*E*	35	37	69

**Table 4:** Crystal data and structure refinement details for ***t*-Bu-19-*o*-OH**, ***t*-Bu-19-*p*-OH**, ***t*-Bu-20-ester** and ***t*-Bu-17-*p*-OH**.

	*t*-Bu-19-*o*-OH	*t*-Bu-19-*p*-OH	*t*-Bu-20-ester	*t*-Bu-17-*p*-OH
CCDC	2112902	2105497	2105498	2130657
Crystal data				
Chemical formula	C_28_H_40_N_2_O_6_	C_28_H_40_N_2_O_6_	C_28_H_40_N_2_O_6_	C_28_H_40_O_6_
*M*_r_	500.62	500.62	500.62	472.60
Crystal system, space group	Monoclinic, *P*2_1_/*n*	$\mathrm{Triclinic},\;P\bar{1}$	Monoclinic, *P*2_1_/*c*	Monoclinic, *P*2_1_/*c*
Temperature (K)	120	120	120	120
*a*, *b*, *c* (Å)	13.432 (2), 15.7712 (18), 26.177 (3)	9.7213 (10), 16.1636 (13), 19.3472 (16)	19.5034 (7), 17.5123 (11), 7.891 (2)	17.208 (2), 15.999 (3), 10.021 (5)
a, b, γ (°)	90, 97.077 (11), 90	67.341 (6), 77.329 (7), 74.107 (7)	90, 99.288 (9), 90	90, 100.597 (19), 90
*V* (Å^3^)	5503.0 (13)	2675.9 (4)	2659.8 (8)	2711.8 (13)
*Z*	8	4	4	4
Radiation type	Cu *K*a	Mo *K*a	Mo *K*a	Mo *K*a
m (mm^−1^)	0.69	0.09	0.09	0.08
Crystal size (mm)	0.34 × 0.18 × 0.05	0.09 × 0.05 × 0.03	0.34 × 0.23 × 0.05	0.12 × 0.06 × 0.02
Data collection				
Diffractometer	STOE *IPDS* 2T	STOE *IPDS* 2T	STOE *IPDS* 2T	STOE *IPDS* 2T
Absorption correction	Integration *	–	–	–
*T*_min_, *T*_max_	0.782, 0.979	–	–	–
No. of measured, independent and observed [*I* > 2s(*I*)] reflections	22476, 9605, 7767	19172, 9458, 3910	7178, 7178, 5882	16293, 4920, 2659
*R*_int_	0.022	0.129	?	0.089
(sin θ/λ)_max_ (Å^−1^)	0.600	0.597	0.687	0.601
Refinement				
*R*[*F*^2^ > 2s(*F*^2^)], *wR*(*F*^2^), *S*	0.049, 0.132, 1.04	0.098, 0.315, 0.99	0.077, 0.201, 1.15	0.062, 0.154, 1.01
No. of reflections	9605	9458	7178	4920
No. of parameters	669	669	336	351
No. of restraints	2	2	1	4
H atom treatment	H atoms treated by a mixture of independent and constrained refinement	H atoms treated by a mixture of independent and constrained refinement	H atoms treated by a mixture of independent and constrained refinement	H atoms treated by a mixture of independent and constrained refinement
Δρ_max_, Δρ_min_ (e Å^−3^)	0.80, −0.31	0.35, −0.31	0.39, −0.39	0.22, −0.22

* STOE *X-RED32*, absorption correction by Gaussian integration, analogous to [35].

## Data Availability

Crystallographic data for all structures reported in this paper have been deposited with the Cambridge Crystallographic Data Centre as supplementary publication No. CCDC 2112902 for ***t*-Bu-19-*o*-OH**, 2105497 for ***t*-Bu-19-*p*-OH**, 2105498 for ***t*-Bu-20-ester** and 2130657 for ***t*-Bu-17-*p*-OH**. The data can be obtained free of charge from The Cambridge Crystallographic Data Centre via www.ccdc.cam.ac.uk/structures (accessed on 9 March 2022).

## Supplementary Materials

The following supporting information can be downloaded at: https://www.mdpi.com/article/10.3390/molecules27061835/s1, Figure S1a: ^1^H NMR spectrum of ***t*-Bu-19-*p*-OH** in acetonitrile-*d*_3_, Figure S1b: gHSQCAD spectrum of ***t*-Bu-19-*p*-OH** in acetonitrile-*d*_3_, Figure S1c: gHMBCAD spectrum of ***t*-Bu-19-*p*-OH** in acetonitrile-*d*_3_, Figure S1d: ROESYAD spectrum of ***t*-Bu-19-*p*-OH** in acetonitrile-*d*_3_, Figure S1e: ^1^H NMR spectrum of ***t*-Bu-19-*p*-OH**) in DMSO-*d*_6_ (bottom–spectrum with amplification of signals), Figure S1f: gHSQCAD spectrum of ***t*-Bu-19-*p*-OH** in DMSO-*d*_6_, Figure S1g: Overlapped gHSQCAD and gHMBCAD spectra of ***t*-Bu-19-*p*-OH** in DMSO-*d*_6_, Figure S1h: ROESYAD spectrum of ***t*-Bu-19-*p*-OH** in DMSO-*d*_6_, Figure S1i: ^13^C NMR spectrum of ***t*-Bu-19-*p*-OH** in DMSO-*d_6_*, Figure S1j: ^1^H NMR spectrum of ***t*-Bu-19-*p*-OH** in acetone-*d*_6_, Figure S1k: ^13^C NMR spectrum of ***t*-Bu-19-*p*-OH** in acetone-*d*_6_, Figure S1l: MS (ESI) spectrum of ***t*-Bu-19-*p*-OH**, Figure S1m: FTIR spectrum (film) of ***t*-Bu-19-*p*-OH,** Figure S2a: ^1^H NMR spectrum of***t*****-Bu-19-*o*-OH** (6.3 × 10^−3^ M) in acetonitrile-*d_3_*, Figure S2b: ^1^H NMR spectrum of ***t*-Bu-19-*o*-OH** in DMSO-*d_6_*, Figure S2c: ^13^C NMR spectrum of **t-Bu-19-*o*-OH** in DMSO-*d_6_*, Figure S2d: MS (ESI) spectrum of ***t*-Bu-19-*o*-OH**, Figure S2e: FTIR spectrum (film) of ***t*-Bu-19-*o*-OH,** Figure S3a: ^1^H NMR spectrum of ***t*-Bu-20-ester** in DMSO-*d_6_*, Figure S3b: ^1^H NMR spectrum of ***t*-Bu-20-ester** in DMSO-*d_6_*, Figure S3c: ^13^C NMR spectrum of ***t*-Bu-20-ester** in DMSO-*d_6_*, Figure S3d: ^13^C NMR spectrum of ***t*-Bu-20-ester** in DMSO-*d_6_*, Figure S3e: ROESY spectrum of ***t*-Bu-20-ester** in DMSO-*d_6_*, Figure S3f: GHSQC spectrum of ***t*-Bu-20-ester** in DMSO-*d_6_*, Figure S3g: GHMBC spectrum of ***t*-Bu-20-ester** in DMSO-*d_6_*, Figure S3h: MS (ESI) spectrum of ***t*-Bu-20-ester**, Figure S3i: FTIR spectrum (film) of ***t*-Bu-20-ester**, Figure S4a: ^1^H NMR spectrum of ***t*-Bu-19-al** in DMSO-*d_6_*, Figure S4b: ^1^H NMR spectrum of ***t*-Bu-19-al** in acetone-*d_6_*, Figure S4c: ^13^C NMR spectrum of ***t*-Bu-19-al** in DMSO-*d_6_*, Figure S4d: ^13^C NMR spectrum of ***t*-Bu-19-al** in acetone-*d_6_*, Figure S4e: ROESY spectrum of ***t*-Bu-19-al** in acetone-*d_6_*, Figure S4f: gHSQC spectrum of ***t*-Bu-19-al** in acetone-*d_6_*, Figure S4g: gHMBC spectrum of ***t*-Bu-19-al** in acetone-*d_6_*, Figure S4h: MS (ESI) spectrum of ***t*-Bu-19-al**, Figure S4i: FTIR spectrum (film) of ***t*-Bu-19-al**, Figure S5a: ^1^H NMR spectrum of ***t*-Bu-17-*p*-OH** in acetone-*d_6_*, Figure S5b: ^13^C NMR spectrum of ***t*-Bu-17-p-OH** in DMSO-*d_6_*, Figure S5c: gHMBC spectrum of ***t*-Bu-17-*p*-OH** in DMSO-*d_6_*, Figure S5d: gHSQC spectrum of ***t*-Bu-17-*p*-OH** in DMSO-*d_6_*, Figure S5e: MS (ESI) spectrum of ***t*-Bu-17-*p*-OH**, Figure S5f: FTIR spectrum (film) of ***t*-Bu-17-*p*-OH**, Table S1: Spectral–UV-Vis absorption-characteristics of ***t*-Bu-19-*p*-OH**, ***t*-Bu-19-*o*-OH**, ***t*-Bu-20-al**, ***t*-Bu-20-ester**, ***t*-Bu-17-*p*-OH *t*-Bu-19-Azo**, ***t*-Bu-19-Azo-O** (acetonitrile), Table S2: The comparison of positions of bands **λ_max_** [nm] in UV-Vis absorption and emission spectra of ***t*-Bu-19-*p*-OH** in different solvents (in parentheses: values of molar absorption coefficients, ε_max_ [dm^3^⋅mol^−1^⋅cm^−1^]). Stokes shift [nm]-the difference in the position of emission and absorption bands, Figure S6: Changes in UV-Vis spectra upon titration of ***t*-Bu-19-*p*-OH** with metal perchlorates in acetonitrile: (a) ***t*-Bu-19-*p*-OH** (1.56 × 10^−5^ M), lithium (0–1.99 × 10^−3^ M); (b) ***t*-Bu-19-*p*-OH** (1.56 × 10^−5^ M), sodium (0–1.6 × 10^−3^ M); (c) ***t*-Bu-19-*p*-OH** (1.56 × 10^−5^ M), potassium (0–4.3 × 10^−4^ M); (d) ***t*-Bu-19-*p*-OH** (1.49 × 10^−5^ M), magnesium (0–1.97 × 10^−3^ M); (e) ***t*-Bu-19-*p*-OH** (1.49 × 10^−5^ M), calcium (0–2.62 × 10^−5^ M); (f) ***t*-Bu-19-*p*-OH** (1.44 × 10^−5^ M), strontium (0–2.62 × 10^−5^ M); (g) ***t*-Bu-19-*p*-OH** (1.66 × 10^−5^ M); barium (0–5.22 × 10^−5^ M), Figure S7: The influence of the presence of acid (*p*-toluenesulfonic acid), base (tetra-n-butylammonium hydroxide) and metal perchlorates on the color of solution of ***t*-Bu-19-*p*-OH** in acetonitrile **(**quantitative probe acid, base and metal perchlorates were added in excess as solids to solution of crown 1.56 × 10^−5^ M), Figure S8: ^1^H NMR spectrum of ***t*-Bu-19-*p*-OH** (8.60 × 10^−3^ M)-top and spectrum registered in the presence of 10-fold excess of sodium perchlorate-bottom (acetonitrile-*d_3_*), Table S3: Comparison of stability constants (log K) of complexes (1:1) of 19-membered crowns in acetonitrile, Figure S9: Changes in UV-Vis spectra upon titration of ***t*-Bu-19-*o*-OH** (7.33 × 10^−5^ M) with metal perchlorates in acetonitrile: (a) lithium (0–1.55 × 10^−3^ M); (b) sodium (0–5.88 × 10^−4^ M); (c) potassium (0–6.14 × 10^−4^ M); (d) magnesium (0–1.77 × 10^−3^ M); (e) calcium (0–1.19 × 10^−4^ M); (f) strontium (0–1.05 × 10^−4^ M); (g) barium (0–1.08 × 10^−4^ M), Figure S10: ^1^H NMR spectra of ***t*-Bu-19-Azo**. Top: crown concentration 5.9 × 10^−3^ M, ratio of isomers *E* 31% and *Z* 69% in acetonitrile-*d_3_*; bottom: crown concentration 5.6 × 10^−3^ M, ratio of isomers *E* 45% and *Z* 55% in DMSO-*d_6_*, Figure S11: Changes in UV-Vis spectra upon titration of ***t*-Bu-19-Azo** with metal perchlorates in acetonitrile: (a) ***t*-Bu-19-Azo** (1.17 × 10^−4^ M), lithium (0–2.47 × 10^−3^ M); (b) ***t*-Bu-19-Azo** (8.17 × 10^−5^ M), sodium (0–3.87 × 10^−4^ M); (c) ***t*-Bu-19-Azo** (9.05 × 10^−5^ M), potassium (0–3.46 × 10^−4^ M); (d) ***t*-Bu-19-Azo** (9.05 × 10^−5^ M), magnesium (0–1.73 × 10^−3^ M); (e) ***t*-Bu-19-Azo** (1.17 × 10^−4^ M), calcium (0–5.38 × 10^−5^ M); (f) ***t*-Bu-19-Azo** (1.17 × 10^−4^ M), strontium (0–6.08 × 10^−5^ M); (g) ***t*-Bu-19-Azo** (1.17 × 10^−4^ M), barium (0–5.93 × 10^−5^ M), Figure S12: ^1^H NMR spectra of ***t*-Bu-19-Azo** (5.9 × 10^−3^ M) registered in the presence of equimolar amount of sodium perchlorate (top) and 10-fold excess of this salt (bottom) in acetonitrile-*d_3_*, Figure S13: Changes in UV-Vis spectra upon titration of ***t*-Bu-19-Azo-O** with metal perchlorates in acetonitrile: (a) ***t*-Bu-19-Azo-O** (2.26 × 10^−4^ M), lithium (0–1.98 × 10^−4^ M); (b) ***t*-Bu-19-Azo-O** (2.83 × 10^−4^ M), sodium (0–4.47 × 10^−4^ M); (c) ***t*-Bu-19-Azo-O** (2.26 × 10^−4^ M), potassium (0–3.21 × 10^−4^ M); (d) ***t*-Bu-19-Azo-O** (2.83 × 10^−4^ M), magnesium (0–2.87 × 10^−3^ M); (e) ***t*-Bu-19-Azo-O** (2.83 × 10^−4^ M), calcium (0–1.93 × 10^−4^ M); (f) ***t*-Bu-19-Azo-O** (2.26 × 10^−4^ M), strontium (0–2.03 × 10^−4^ M);(g) ***t*-Bu-19-Azo-O** (2.83 × 10^−4^ M), barium (0–2.24 × 10^−4^ M), Figure S14: Exemplary spectra showing changes in UV-Vis upon titration of ***t*-Bu-20-ester** (1.02 × 10^−4^ M) with metal perchlorates in acetonitrile: (a) lithium (0–3.48 × 10^−3^ M); (b) sodium (0–2.14 × 10^−3^ M); (c) potassium (0–1.12 × 10^−4^ M); (d) magnesium (0–7.27 × 10^−4^ M); (e) calcium (0–1.03 × 10^−3^ M); (f) strontium (0–5.77 × 10^−4^ M).

## Sample Availability

Samples of the compounds are not available from authors.

## References

[1] Yue L., Yang K., Lou X.Y., Yang Y.W., Wang R. (2020). Versatile roles of macrocycles in organic-inorganic hybrid materials for biomedical applications. Matter.

[2] Sabah K.J., Zahid N.I., Hashim R. (2021). Synthesis of new chiral macrocycles-based glycolipids and its application in asymmetric Michael addition. Res. Chem. Intermed..

[3] Shang J., Liu Y., Pan T. (2021). Macrocycles in bioinspired catalysis: From molecules to materials. Front. Chem..

[4] Jiang C., Song Z., Yu L., Ye S., He H. (2020). Fluorescent probes based on macrocyclic hosts: Construction, mechanism and analytical applications. Trends Anal. Chem..

[5] Tay H.M., Beer P. (2021). Optical sensing of anions by macrocyclic and interlocked hosts. Org. Biomol. Chem..

[6] Yang Z., Liu Z., Yuan L. (2021). Recent advances of photoresponsive supramolecular switches. Asian J. Chem..

[7] Sokołowska P., Dąbrowa K., Jarosz S. (2021). Visible-Light Responsive Sucrose-Containing Macrocyclic Host for Cations. Org. Lett..

[8] Luboch E., Bilewicz R., Kowalczyk M., Wagner-Wysiecka E., Biernat J.F., Gokel G.W. (2003). Azo Macrocyclic Compounds. Advances in Supramolecular Chemistry.

[9] Wagner-Wysiecka E., Łukasik N., Biernat J.F., Luboch E. (2018). Azo group(s) in selected macrocyclic compounds. J. Incl. Phenom. Macrocycl. Chem..

[10] Benkhaya S., M’rabet S., El Harfi A. (2020). Classifications, properties, recent synthesis and applications of azo dyes. Heliyon.

[11] Yu J., Qi D., Li J. (2020). Design, synthesis and applications of responsive macrocycles. Commun. Chem..

[12] Liu Y., Wang H., Liu P., Zhu H., Shi B., Hong X., Huang F. (2021). Azobenzene-based macrocyclic arenes: Synthesis, crystal structures, and light-controlled molecular encapsulation and release. Angew. Chem..

[13] Alehashem M.S., Ariffin A.B., Savage P.B., Dabdawb W.A.Y., Thomas N.F. (2020). Treasures old and new: What we can learn regarding the macrocyclic problem from past and present efforts in natural product total synthesis. RSC Adv..

[14] Shiga M., Nakamura H., Takagi M., Ueno K. (1984). Synthesis of azobenzo-crown ethers and their complexation behavior with metal ions. Bull. Chem. Soc. Jpn..

[15] Tahara R., Morozumi T., Nakamura H., Shimomura M. (1997). Photoisomerization of azobenzocrown ethers. Effect of complexation of alkaline earth metal ions. J. Phys. Chem. B..

[16] Simonov Y.A., Luboch E., Biernat J.F., Bolotina N.V., Zavodnik V.E. (1997). Inclusion compounds of NaI with 13-membered azo- and azoxycrown ethers. J. Incl. Phenom. Macrocycl. Chem..

[17] Skwierawska A., Luboch E., Biernat J.F., Kravtsov V.C., Simonov Y.A., Dvorkin A.A., Bel’skii V.K. (1998). Stereochemistry of 16-membered azo- and azoxycrown ethers. Structures of their sandwich potassium iodide complexes. J. Incl. Phenom. Macrocycl. Chem..

[18] Luboch E., Biernat J.F., Kravtsov V.C., Simonov Y.A. (1998). 13-Membered azocrown ether. Structure of the lithium bromide complex and membrane properties. J. Incl. Phenom. Macrocycl. Chem..

[19] Luboch E., Biernat J.F., Kravtsov V.C., Simonov Y.A., Belskii V.K. (1999). Structures of NaI complexes of 16-membered azo- and azoxycrown ethers. Correlation of crystal structure and carrier-doped membrane electrode selectivity. Supramol. Chem..

[20] Luboch E., Wagner-Wysiecka E., Poleska-Muchlado Z., Kravtsov V.C. (2005). Synthesis and properties of azobenzocrown ethers with pi-electron donor, or pi-electron donor and pi-electron acceptor group(s) on benzene rings. Tetrahedron.

[21] Luboch E. (2008). The Wallach rearrangement as a method for the synthesis of functionalized azobenzocrown ethers. Pol. J. Chem..

[22] Luboch E., Wagner-Wysiecka E., Rzymowski T. (2009). 4-Hexylresorcinol-derived hydroxyazobenzocrown ethers as chromoionophores. Tetrahedron.

[23] Szarmach M., Wagner-Wysiecka E., Fonari M.S., Luboch E. (2012). Bis(azobenzocrown ether)s—Synthesis and ionophoric properties. Tetrahedron.

[24] Szarmach M., Wagner-Wysiecka E., Luboch E. (2013). Rearrangement of azoxybenzocrowns into chromophoric hydroxyazobenzocrowns and the use of hydroxyazobenzocrowns for the synthesis of ionophoric biscrown compounds. Tetrahedron.

[25] Wagner-Wysiecka E., Szarmach M., Chojnacki J., Łukasik N., Luboch E. (2017). Cation sensing by diphenyl-azobenzocrowns. J. Photochem. Photobiol. A—Chem..

[26] Wagner-Wysiecka E., Szulc P., Luboch E., Chojnacki J., Szwarc-Karabyka K., Łukasik N., Murawski M., Kosno M. (2020). Photochemical rearrangement of a 19-membered azoxybenzocrown: Products and their properties. ChemPlusChem.

[27] Wallach O., Belli L. (1880). Ueber die Umwandlung von Azoxybenzol in Oxyazobenzol. Ber. Dtsch. Chem. Ges..

[28] Badger G.M., Buttery R.G. (1954). Aromatic azo-compounds. Part VI. The action of light on azoxy-compounds. J. Chem. Soc..

[29] Shemyakin M.M., Agadzhanyan T.E., Maimind V.I., Kudryavtsev R.V. (1963). Study of the mechanism of the para and ortho rearrangements of substituted azoxy compounds. Russ. Chem. Bull..

[30] Bhatnagar A., Schroder A., Mohanty D.K. (1997). Azoxyaromatic polyethers. Polymer.

[31] Yamamoto J., Hamada R., Tsuboi T. (2002). Photochemical reaction of azoxybenzenes in the presence of acetic acid. Nippon Kagaku Kaishi.

[32] Yamamoto J., Sakamoto T., Kusunoki K., Umezu M., Matsuura T. (1977). The Wallach Rearrangement of a few azoxy compounds. Nippon Kagaku Kaishi.

[33] Bunce N.J. (1977). On the involvement of diazonium ions in the photorearrangement of azoxybenzene. Can. J. Chem..

[34] Skwierawska A.M., Biernat J.F., Kravtsov V.C. (2006). Synthesis and electrode properties of 19-membered azo- and azoxycrown ethers. Structure of dibenzo-19-azocrown-7. Tetrahedron.

[35] Coppens P., Ahmed F.R. (1970). The evaluation of absorption and extinction in single-crystal structure analysis. Crystallographic Computing.

[36] Dolomanov O.V., Bourhis L.J., Gildea R.J., Howard J.A.K., Puschmann H. (2009). OLEX2: A complete structure solution, refinement and analysis program. J. Appl. Crystallogr..

[37] Sheldrick G.M. (2008). A short history of SHELX. Acta Cryst. A.

[38] Sheldrick G.M. (2015). SHELXT—Integrated space-group and crystal-structure determination. Acta Cryst. C.

[39] Kyvala M., Lukes I. Program package “OPIUM”. https://web.natur.cuni.cz/~kyvala/opium.html.

