# Supplementary material for: Products of Photo- and Thermochemical Rearrangement of 19-Membered di-tert-Butyl-Azoxybenzocrown
Source: Molecules. 2022 Mar 11;27(6):1835. doi: 10.3390/molecules27061835 (PMC8955022; doi:10.3390/molecules27061835)
Supplement: Supplementary file 1 [file molecules-27-01835-s001.zip › molecules-1623117-supplementary.pdf]

Supplementary data

Products of Photo and Thermal Rearrangement of 19-Membered di-*tert*-Butyl-Azoxybenzocrown

Ewa Wagner-Wysiecka[1*], Paulina Szulc[1], Elżbieta Luboch[1*], Jarosław Chojnacki[2], Paweł Sowiński[3] and Katarzyna Szwarc-Karabyka[3]

[1]Department of Chemistry and Technology of Functional Materials, Faculty of Chemistry, Gdańsk University of Technology, Narutowicza Street 11/12, 80-233 Gdańsk, Poland
[2]Department of Inorganic Chemistry, Faculty of Chemistry, Gdańsk University of Technology, Narutowicza Street 11/12, 80-233 Gdańsk, Poland
[3]Nuclear Magnetic Resonance Laboratory, Faculty of Chemistry, Gdańsk University of Technology, Narutowicza Street 11/12, 80-233 Gdańsk, Poland
*Correspondence: ewa.wagner-wysiecka@pg.edu.pl (E.W.-W.); elzbieta.luboch@pg.edu.pl (E.L.)

Figure S1a. ^{1}H NMR spectrum of ***t*-Bu-19-*p*-OH** in acetonitrile-d_3 4
Figure S1b. gHSQCAD spectrum of ***t*-Bu-19-*p*-OH** in acetonitrile-d_3 5
Figure S1c. gHMBCAD spectrum of ***t*-Bu-19-*p*-OH** in acetonitrile-d_3 6
Figure S1d. ROESYAD spectrum of ***t*-Bu-19-*p*-OH** in acetonitrile-d_3 7
Figure S1e. ^{1}H NMR spectrum of ***t*-Bu-19-*p*-OH** in DMSO-d_6 (bottom – spectrum with amplification of signals) 8
Figure S1f. gHSQCAD spectrum of ***t*-Bu-19-*p*-OH** in DMSO-d_6 9
Figure S1g. Overlapped gHSQCAD and gHMBCAD spectra of ***t*-Bu-19-*p*-OH** in DMSO-d_6 10
Figure S1h. ROESYAD spectrum of ***t*-Bu-19-*p*-OH** in DMSO-d_6 11
Figure S1i. ^{13}C NMR spectrum of ***t*-Bu-19-*p*-OH** in DMSO-d_6 12
Figure S1j. ^{1}H NMR spectrum of ***t*-Bu-19-*p*-OH** in acetone-d_6 13
Figure S1k. ^{13}C NMR spectrum of ***t*-Bu-19-*p*-OH** in acetone-d_6 14
Figure S1l. MS (ESI) spectrum of ***t*-Bu-19-*p*-OH** 15
Figure S1m. FTIR spectrum (film) of ***t*-Bu-19-*p*-OH** 16
Figure S2a. ^{1}H NMR spectrum of ***t*-Bu-19-*o*-OH** (6.3 $\times 10^{-3}$ M) in acetonitrile-d_3 17
Figure S2b. ^{1}H NMR spectrum of ***t*-Bu-19-*o*-OH** in DMSO-d_6 18
Figure S2c. ^{13}C NMR spectrum of **t-Bu-19-*o*-OH** in DMSO-d_6 19
Figure S2d. MS (ESI) spectrum of ***t*-Bu-19-*o*-OH** 20
Figure S2e. FTIR spectrum (film) of ***t*-Bu-19-*o*-OH** 21
Figure S3a. ^{1}H NMR spectrum of ***t*-Bu-20-ester** in DMSO-d_6 22
Figure S3b. ^{1}H NMR spectrum of ***t*-Bu-20-ester** in DMSO-d_6 23
Figure S3c. ^{13}C NMR spectrum of ***t*-Bu-20-ester** in DMSO-d_6 24
Figure S3d. ^{13}C NMR spectrum of ***t*-Bu-20-ester** in DMSO-d_6 25
Figure S3e. ROESY spectrum of ***t*-Bu-20-ester** in DMSO-d_6 26
Figure S3f. GHSQC spectrum of ***t*-Bu-20-ester** in DMSO-d_6 27
Figure S3g. GHMBC spectrum of ***t*-Bu-20-ester** in DMSO-d_6 28
Figure S3h. MS (ESI) spectrum of ***t*-Bu-20-ester** 29
Figure S3i. FTIR spectrum (film) of ***t*-Bu-20-ester** 30
Figure S4a. 1H NMR spectrum of ***t*-Bu-19-al** in DMSO-d$_6$ 31
Figure S4b. ^{1}H NMR spectrum of ***t*-Bu-19-al** in acetone-d_6 32
Figure S4c. ^{13}C NMR spectrum of ***t*-Bu-19-al** in DMSO-d_6 33
Figure S4d. ^{13}C NMR spectrum of ***t*-Bu-19-al** in acetone-d_6 34
Figure S4e. ROESY spectrum of ***t*-Bu-19-al** in acetone-d_6 35
Figure S4f. gHSQC spectrum of ***t*-Bu-19-al** in acetone-d_6 36
Figure S4g. gHMBC spectrum of ***t*-Bu-19-al** in acetone-d_6 37
Figure S4h. MS (ESI) spectrum of ***t*-Bu-19-al** 38
Figure S4i. FTIR spectrum (film) of ***t*-Bu-19-al** 39
Figure S5a. ^{1}H NMR spectrum of ***t*-Bu-17-*p*-OH** in acetone-d_6 40
Figure S5b. ^{13}C NMR spectrum of ***t*-Bu-17-*p*-OH** in DMSO-d_6 41
Figure S5c. gHMBC spectrum of ***t*-Bu-17-*p*-OH** in DMSO-d_6 42
Figure S5d.gHSQC spectrum of ***t*-Bu-17-*p*-OH** in DMSO-d_6 43

Figure S5e. MS (ESI) spectrum of ***t*-Bu-17-*p*-OH** 44

Figure S5f. FTIR spectrum (film) of ***t*-Bu-17-*p*-OH** 45

Table S1. Spectral - UV-Vis absorption - characteristics of *t*-Bu-19-*p*-OH, *t*-Bu-19-*o*-OH, *t*-Bu-20-al, *t*-Bu-20-ester, *t*-Bu-17-*p*-OH, *t*-Bu-19-Azo, *t*-Bu-19-Azo-O (acetonitrile) 46

Table S2. The comparison of positions of bands λ_{max} [nm] in UV-Vis absorption and emission spectra of ***t*-Bu-19-*p*-OH** in different solvents (in parentheses: values of molar absorption coefficients, ε_{max} [$dm^3 \cdot mol^{-1} \cdot cm^{-1}$]). Stokes shift [nm] - the difference in the position of emission and absorption bands 47

Figure S6. Changes in UV-Vis spectra upon titration of ***t*-Bu-19-*p*-OH** with metal perchlorates in acetonitrile: (a) ***t*-Bu-19-*p*-OH** (1.56×10^{-5} M), lithium ($0 - 1.99\times10^{-3}$ M); (b) ***t*-Bu-19-*p*-OH** (1.56×10^{-5} M), sodium ($0 - 1.6\times10^{-3}$ M); (c) ***t*-Bu-19-*p*-OH** (1.56×10^{-5} M), potassium ($0 - 4.3\times10^{-4}$ M); (d) ***t*-Bu-19-*p*-OH** (1.49×10^{-5} M), magnesium ($0 - 1.97\times10^{-3}$M); (e) ***t*-Bu-19-*p*-OH** (1.49×10^{-5} M), calcium ($0 - 2.62\times10^{-5}$ M); (f) ***t*-Bu-19-*p*-OH** (1.44×10^{-5} M), strontium ($0 - 2.62\times10^{-5}$ M); (g) ***t*-Bu-19-*p*-OH** (1.66×10^{-5} M), barium ($0 - 5.22\times10^{-5}$ M) 48

Figure S7. The influence of the presence of acid (*p*-toluenesulfonic acid), base (tetra-n-butylammonium hydroxide) and metal perchlorates on the color of solution of ***t*-Bu-19-*p*-OH** in acetonitrile (quantitative probe acid, base and metal perchlorates were added in excess as solids to solution of crown 1.56×10^{-5} M) 49

Figure S8. ^{1}H NMR spectrum of ***t*-Bu-19-*p*-OH** (8.60×10^{-3} M) - top and spectrum registered in the presence of 10-fold excess of sodium perchlorate - bottom (acetonitrile-d_3) 50

Table S3. Comparison of stability constants (log K) of complexes (1:1) of 19-membered crowns in acetonitrile 51

Figure S9. Changes in UV-Vis spectra upon titration of ***t*-Bu-19-*o*-OH** (7.33×10^{-5} M) with metal perchlorates in acetonitrile: (a) lithium ($0 - 1.55\times10^{-3}$ M); (b) sodium ($0 - 5.88\times10^{-4}$ M); (c) potassium ($0 - 6.14\times10^{-4}$ M); (d) magnesium ($0 - 1.77\times10^{-3}$ M); (e) calcium ($0 - 1.19\times10^{-4}$ M); (f) strontium ($0 - 1.05\times10^{-4}$ M); (g) barium ($0 - 1.08\times10^{-4}$ M) 52

Figure S10. 1H NMR spectra of t-Bu-19-Azo. Top: crown concentration 5.9×10-3 M, ratio of isomers E 31% and Z 69% in acetonitrile-d3; bottom: crown concentration 5.6×10-3 M, ratio of isomers E 45% and Z 55% in DMSO-d_6. 53

Figure S11. Changes in UV-Vis spectra upon titration of ***t*-Bu-19-Azo** with metal perchlorates in acetonitrile: (a) ***t*-Bu-19-Azo** (1.17×10^{-4} M), lithium ($0 - 2.47\times10^{-3}$ M); (b) ***t*-Bu-19-Azo** (8.17×10^{-5} M), sodium ($0 - 3.87\times10^{-4}$ M); (c) ***t*-Bu-19-Azo** (9.05×10^{-5} M), potassium ($0 - 3.46\times10^{-4}$ M); (d) ***t*-Bu-19-Azo** (9.05×10^{-5} M), magnesium ($0 - 1.73\times10^{-3}$ M); (e) ***t*-Bu-19-Azo** (1.17×10^{-4} M), calcium ($0 - 5.38\times10^{-5}$ M); (f) ***t*-Bu-19-Azo** (1.17×10^{-4} M), strontium ($0 - 6.08\times10^{-5}$ M); (g) ***t*-Bu-19-Azo** (1.17×10^{-4} M), barium ($0 - 5.93\times10^{-5}$ M) 54

Figure S12. ^{1}H NMR spectra of ***t*-Bu-19-Azo** (5.9×10^{-3} M) registered in the presence of equimolar amount of sodium perchlorate (top) and 10-fold excess of this salt (bottom) in acetonitrile-d_3. 55

Figure S13. Changes in UV-Vis spectra upon titration of ***t*-Bu-19-Azo-O** with metal perchlorates in acetonitrile: (a) ***t*-Bu-19-Azo-O** (2.26×10^{-4} M), lithium ($0 - 1.98\times10^{-4}$ M); (b) ***t*-Bu-19-Azo-O** (2.83×10^{-4} M), sodium ($0 - 4.47\times10^{-4}$ M); (c) ***t*-Bu-19-Azo-O** (2.26×10^{-4} M), potassium ($0 - 3.21\times10^{-4}$ M); (d) ***t*-Bu-19-Azo-O** (2.83×10^{-4} M), magnesium ($0 - 2.87\times10^{-3}$ M); (e) ***t*-Bu-19-Azo-O** (2.83×10^{-4} M), calcium ($0 - 1.93\times10^{-4}$ M); (f) ***t*-Bu-19-Azo-O** (2.26×10^{-4} M), strontium ($0 - 2.03\times10^{-4}$ M);(g) ***t*-Bu-19-Azo-O** (2.83×10^{-4} M), barium ($0 - 2.24\times10^{-4}$ M) 56

Figure S14. Exemplary spectra showing changes in UV-Vis upon titration of ***t*-Bu-20-ester** (1.02×10^{-4} M) with metal perchlorates in acetonitrile: (a) lithium ($0–3.48\times10^{-3}$ M); (b) sodium ($0–2.14\times10^{-3}$ M); (c) potassium ($0–1.12\times10^{-4}$ M); (d) magnesium ($0 –7.27\times10^{-4}$ M); (e) calcium ($0–1.03\times10^{-3}$ M); (f) strontium ($0–5.77\times10^{-4}$ M) 57

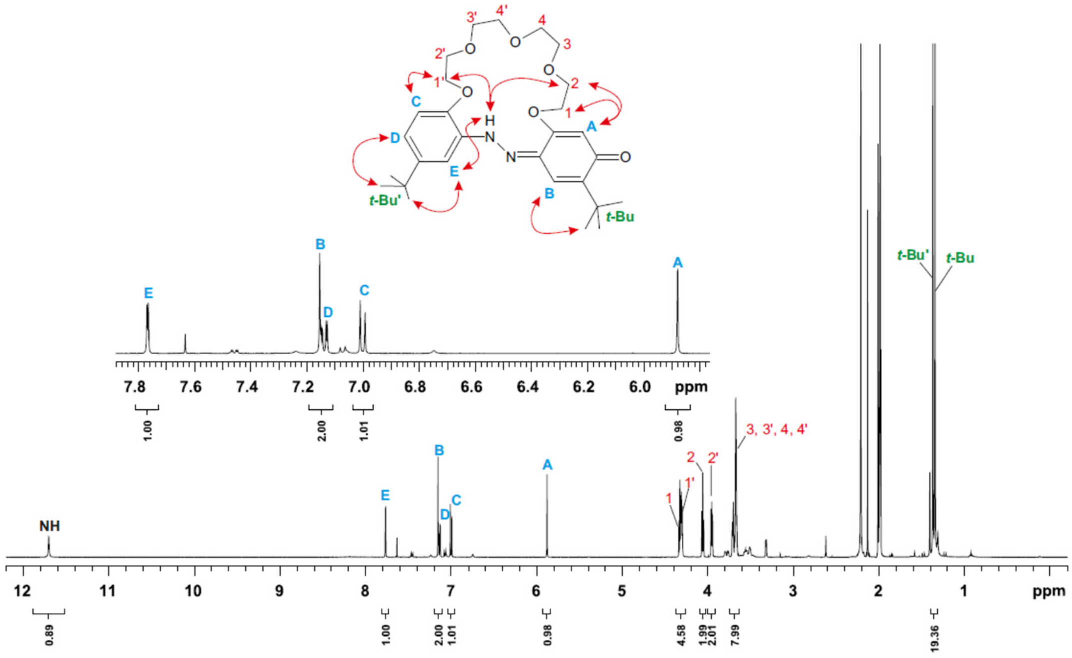

Figure S1a. ^{1}H NMR spectrum of ***t*-Bu-19-*p*-OH** in acetonitrile-d_3

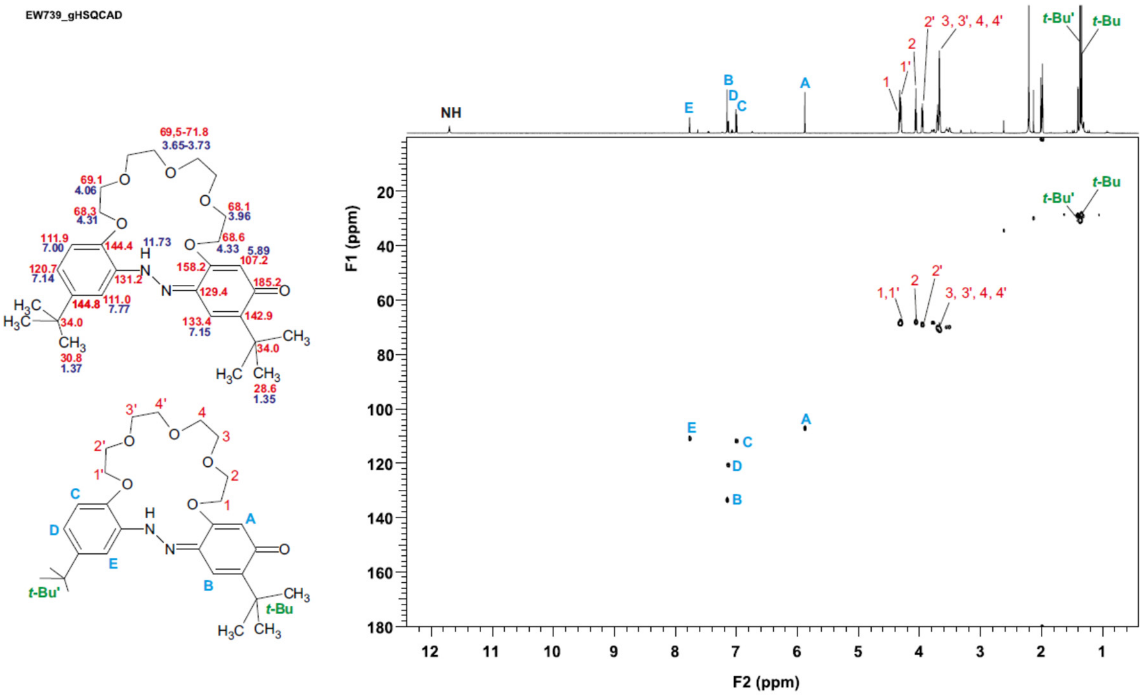

Figure S1b. gHSQCAD spectrum of ***t*-Bu-19-*p*-OH** in acetonitrile-d_3

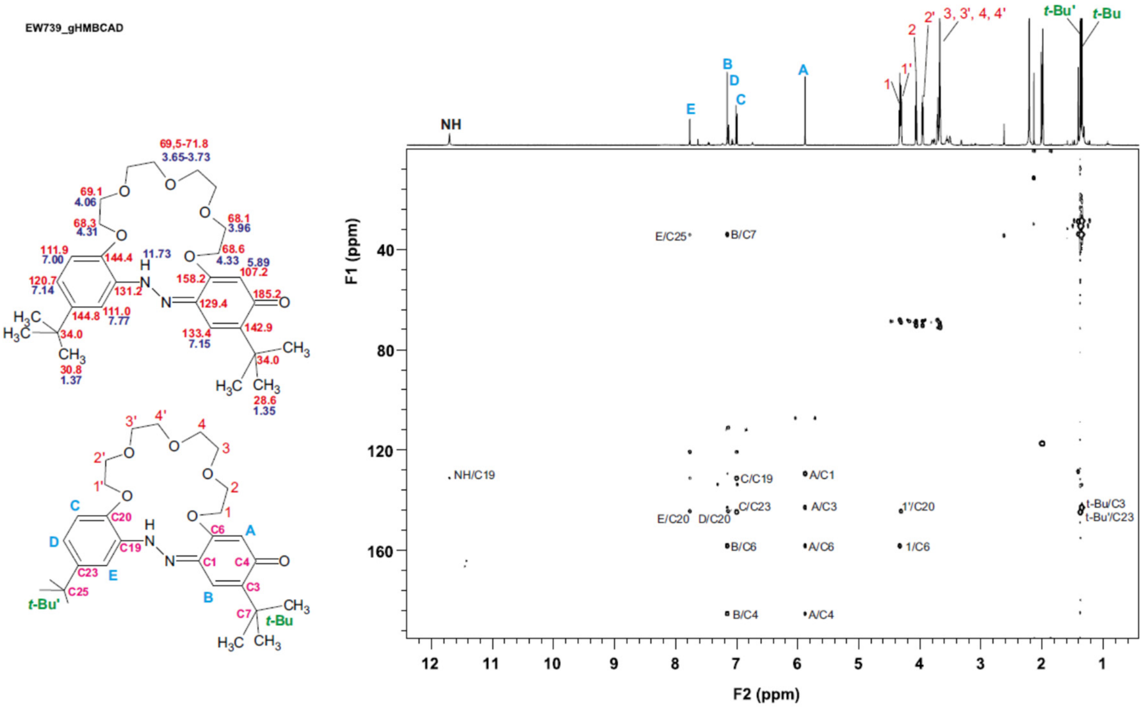

Figure S1c. gHMBCAD spectrum of ***t*-Bu-19-*p*-OH** in acetonitrile-d_3

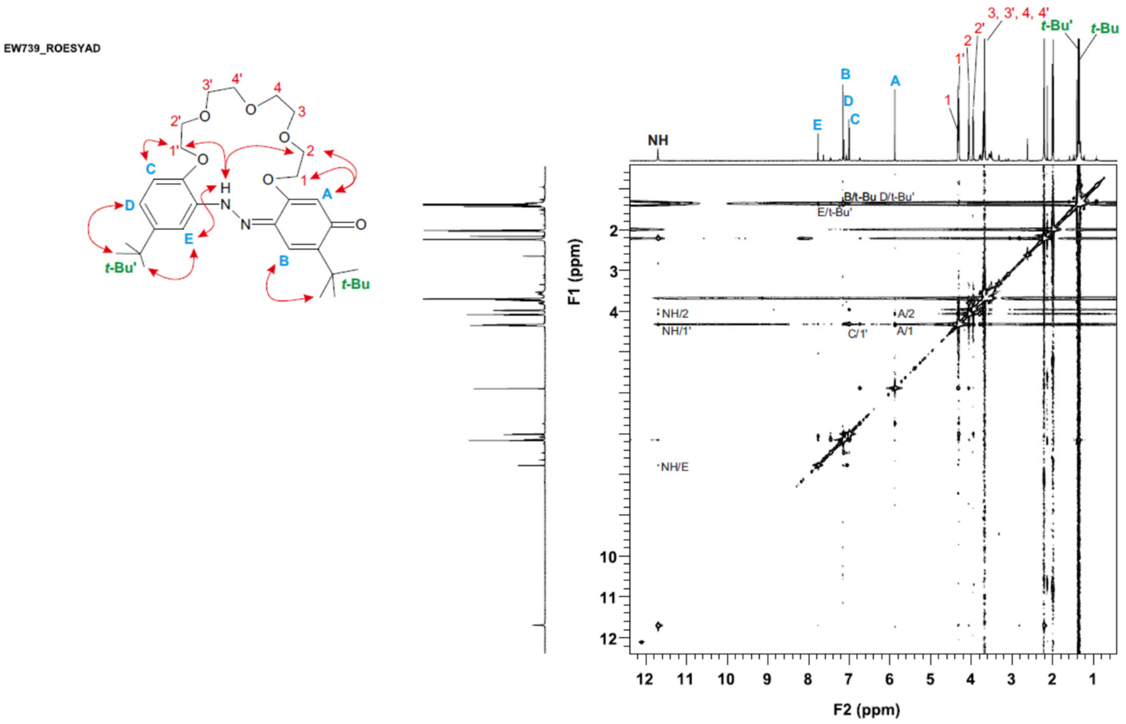

Figure S1d. ROESYAD spectrum of ***t*-Bu-19-*p*-OH** in acetonitrile-d_3

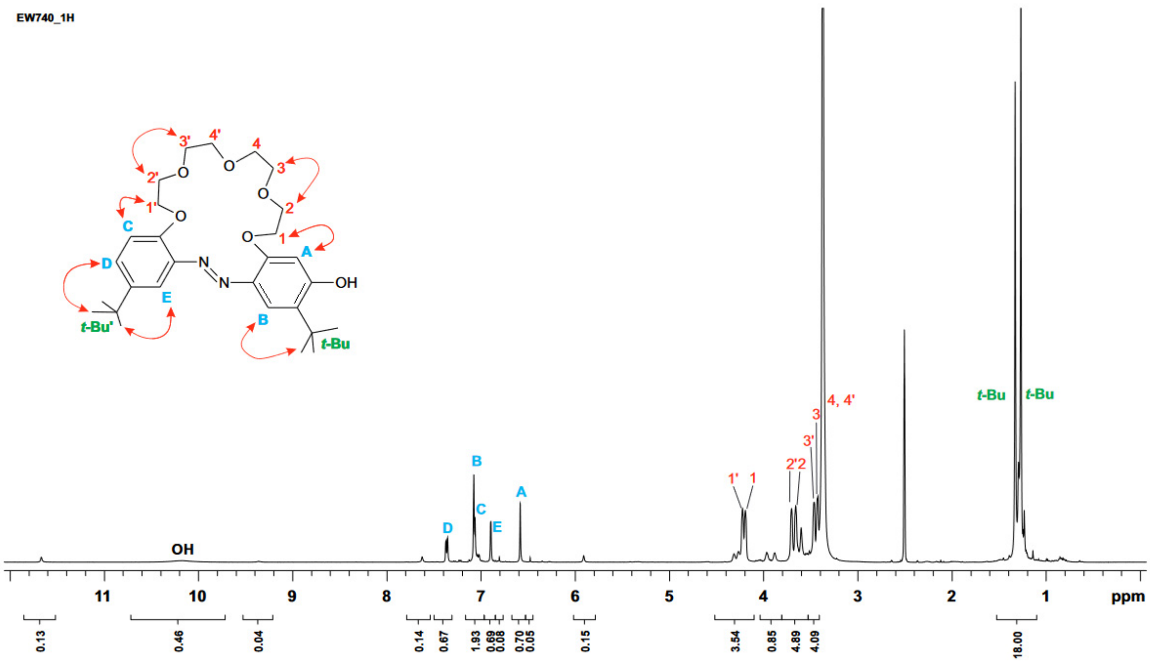

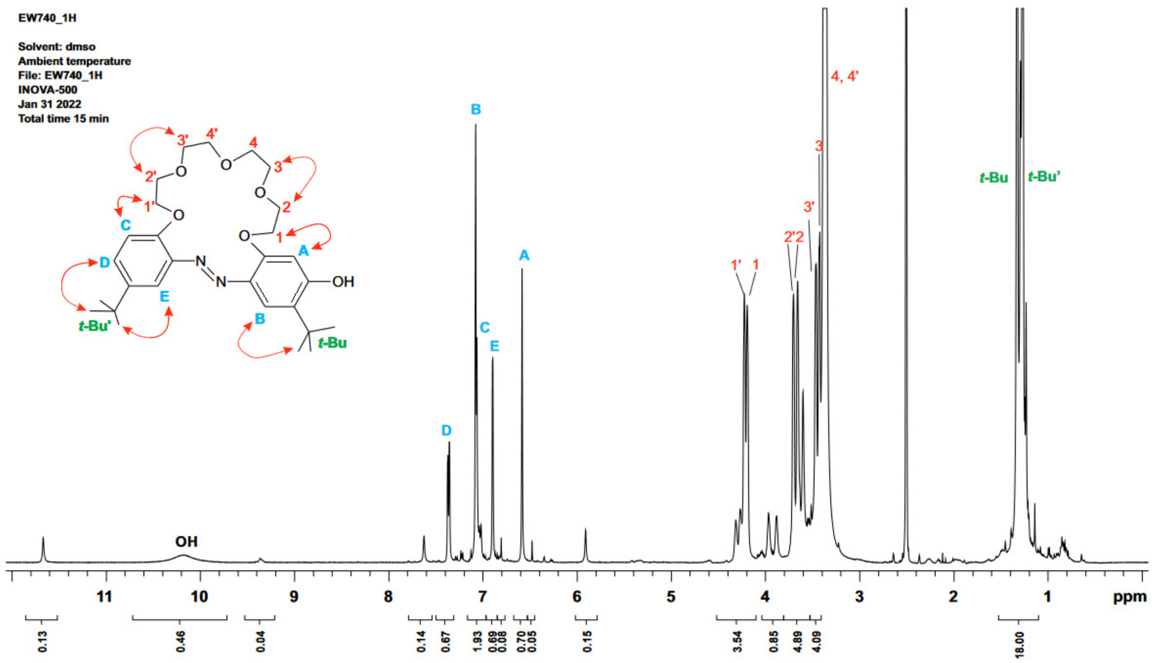

Figure S1e. ^{1}H NMR spectrum of ***t*-Bu-19-*p*-OH** in DMSO-d_6 (bottom – spectrum with amplification of signals)

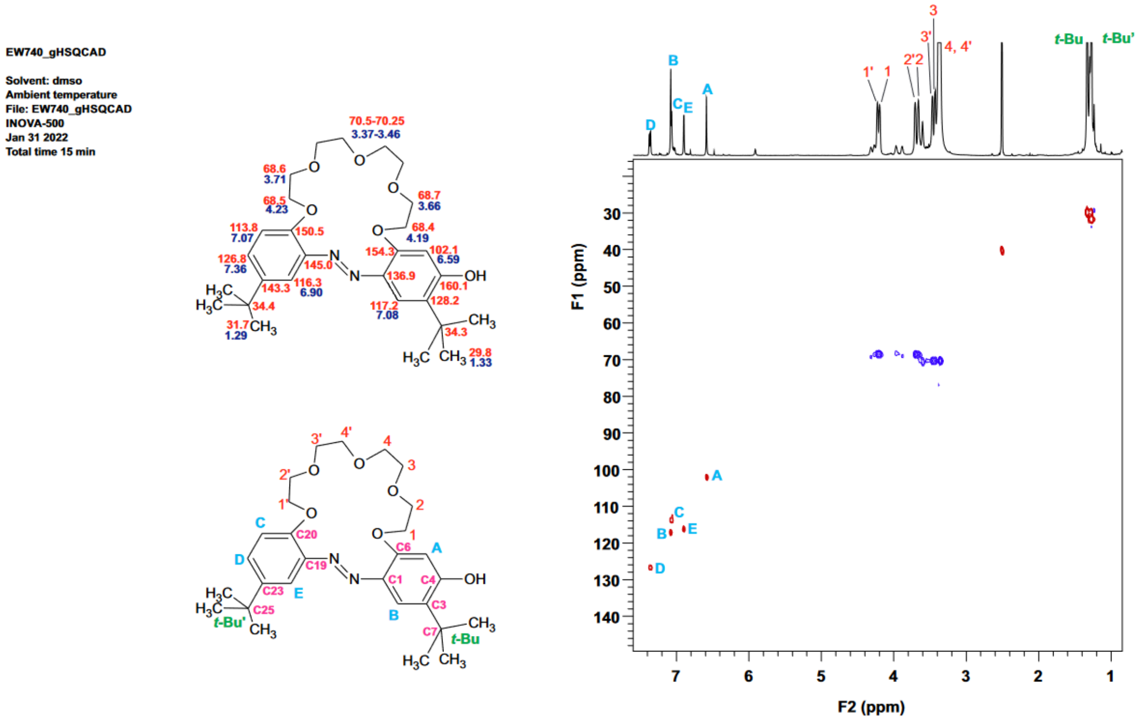

Figure S1f. gHSQCAD spectrum of ***t*-Bu-19-*p*-OH** in DMSO-d_6

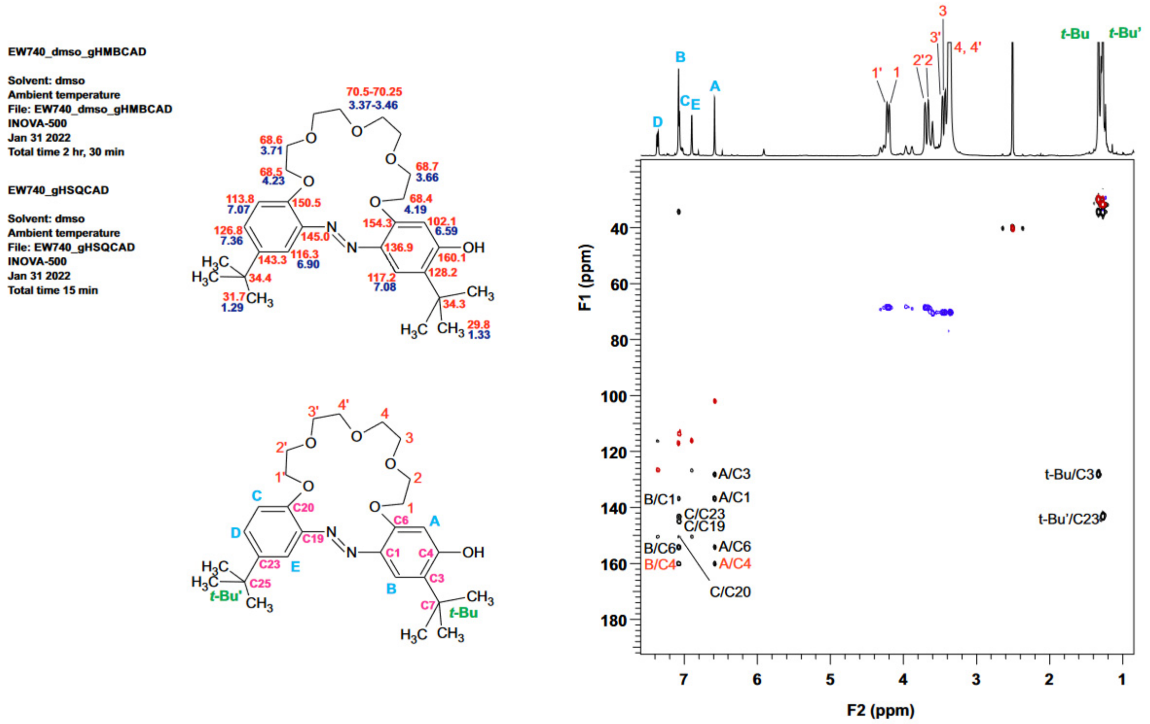

Figure S1g. Overlapped gHSQCAD and gHMBCAD spectra of ***t*-Bu-19-*p*-OH** in DMSO-d_6

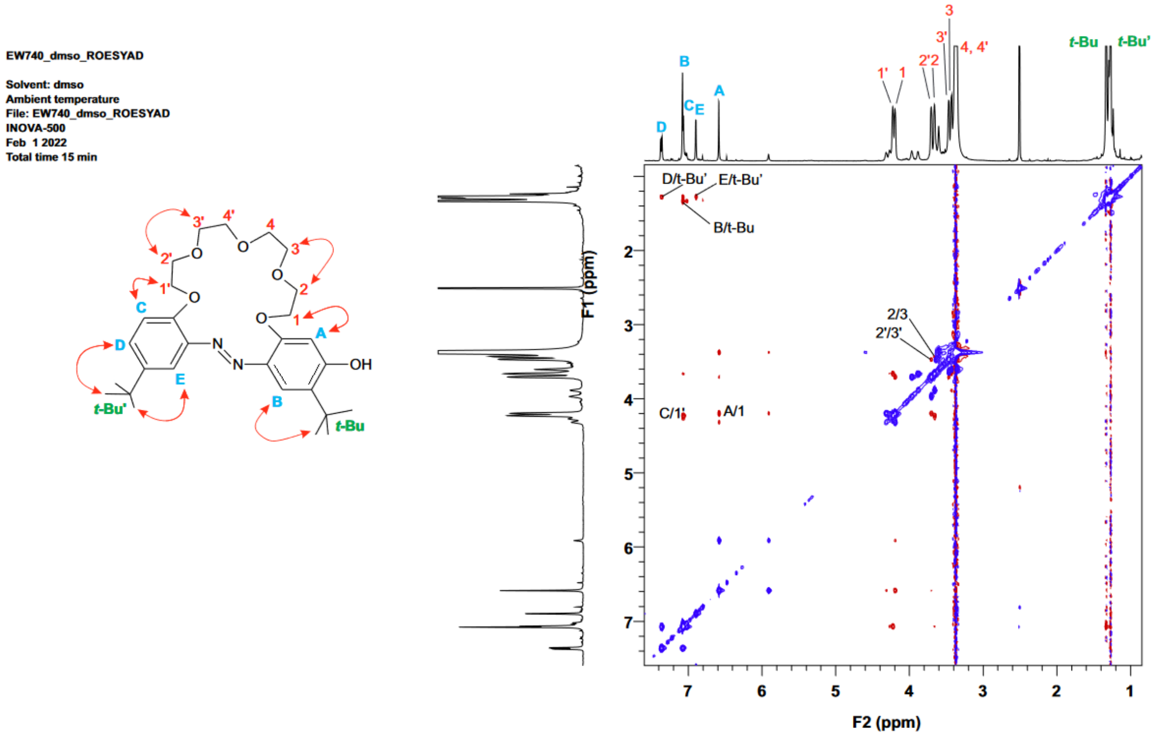

Figure S1h. ROESYAD spectrum of ***t*-Bu-19-*p*-OH** in DMSO-d_6

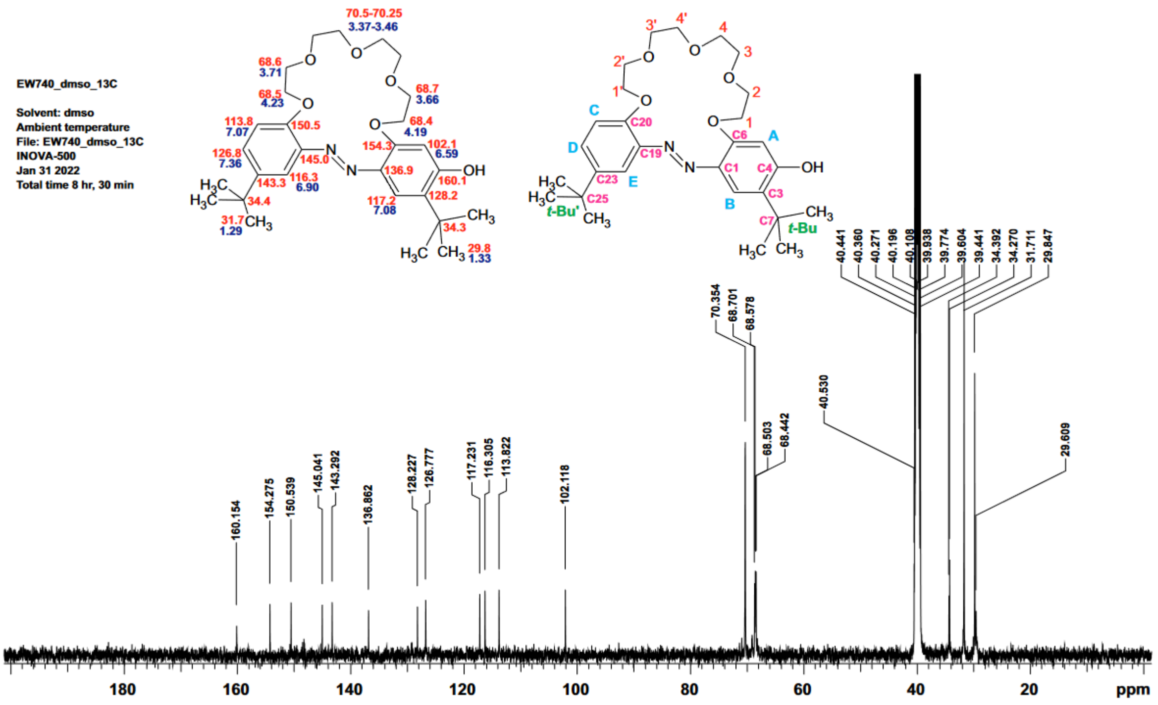

Figure S1i. ^{13}C NMR spectrum of ***t*-Bu-19-*p*-OH** in DMSO-*d*$_6$

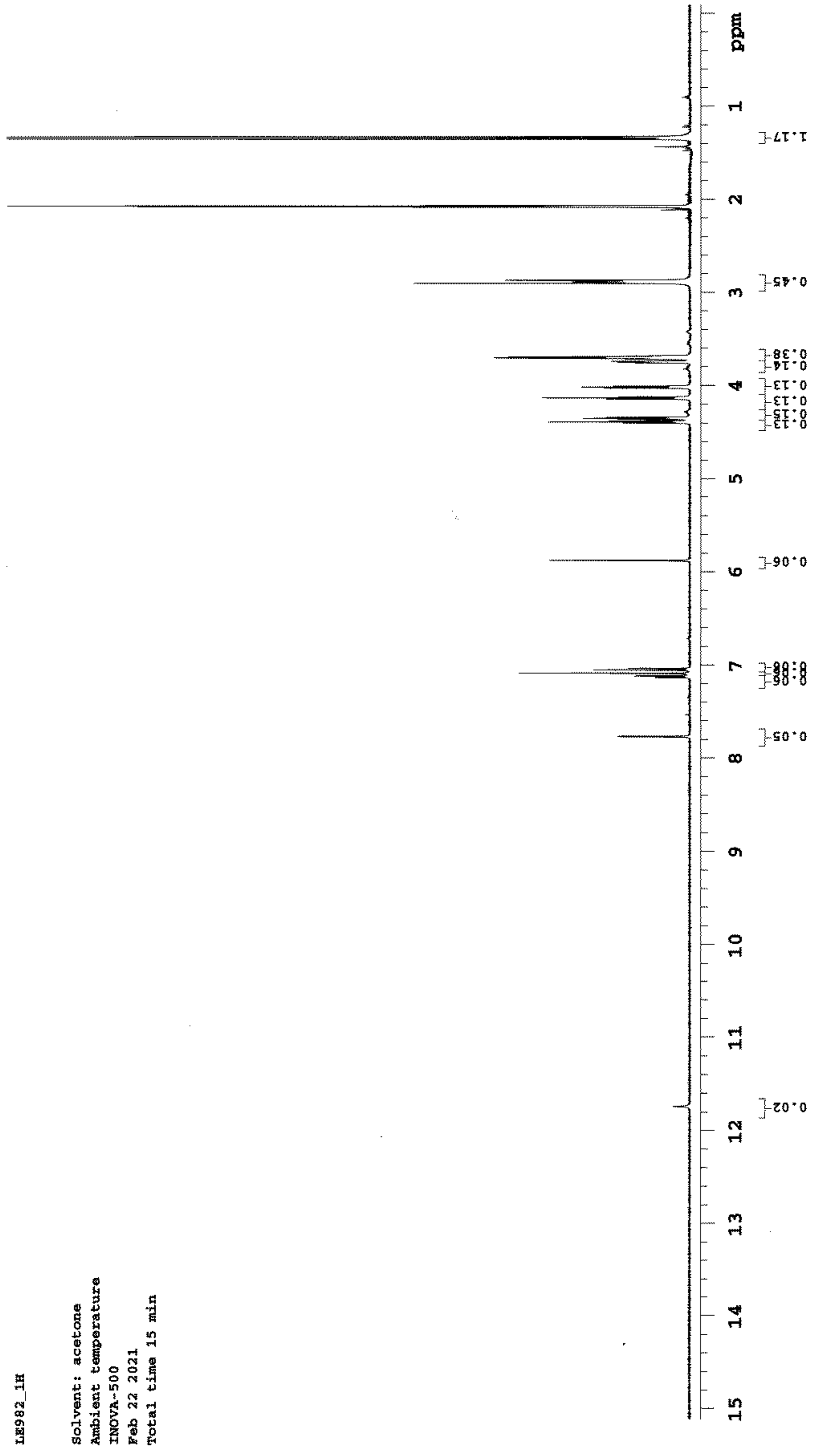

Figure S1j. ^{1}H NMR spectrum of ***t*-Bu-19-*p*-OH** in acetone-d_6

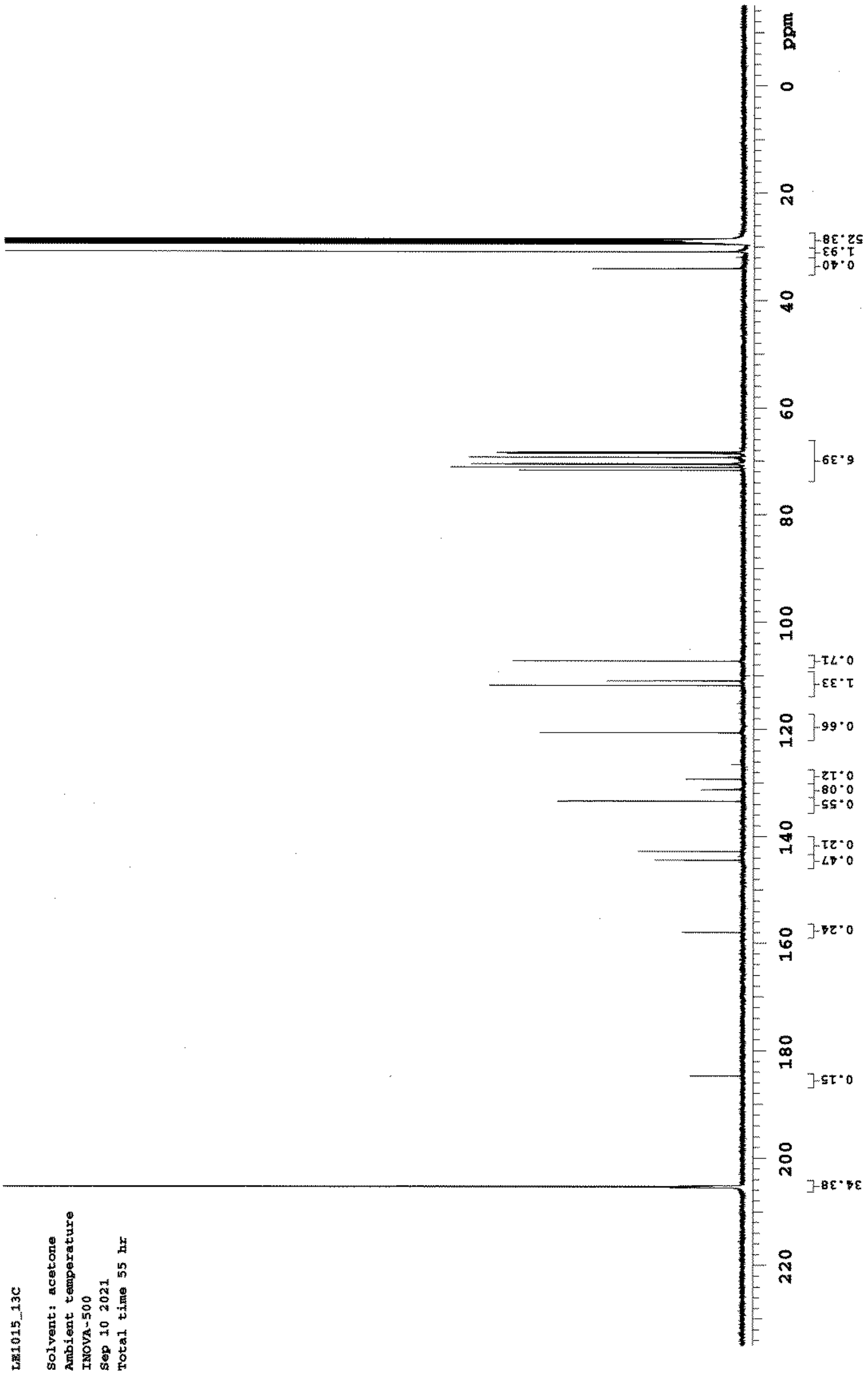

Figure S1k. ^{13}C NMR spectrum of ***t*-Bu-19-*p*-OH** in acetone-d_6

Single Mass Analysis
Tolerance = 3.0 mDa / DBE: min = -1.5, max = 100.0
Element prediction: Off
Number of isotope peaks used for i-FIT = 3
Monoisotopic Mass, Even Electron Ions
130 formula(e) evaluated with 1 results within limits (all results (up to 1000) for each mass)
Elements Used:
C: 0-100 H: 0-200 N: 0-2 O: 0-6 Na: 1-1

Mass	Calc. Mass	mDa	PPM	DBE	Formula	i-FIT	i-FIT Norm	Fit Conf %	C	H	N	O	Na
523.2788	523.2784	0.4	0.8	9.5	C28 H40 N2 O6 Na	53.5	n/a	n/a	28	40	2	6	1

Single Mass Analysis
Tolerance = 3.0 mDa / DBE: min = -1.5, max = 100.0
Element prediction: Off
Number of isotope peaks used for i-FIT = 3
Monoisotopic Mass, Even Electron Ions
132 formula(e) evaluated with 1 results within limits (all results (up to 1000) for each mass)
Elements Used:
C: 0-100 H: 0-200 N: 0-2 O: 0-6

Mass	Calc. Mass	mDa	PPM	DBE	Formula	i-FIT	i-FIT Norm	Fit Conf %	C	H	N	O
501.2972	501.2965	0.7	1.4	9.5	C28 H41 N2 O6	727.9	n/a	n/a	28	41	2	6

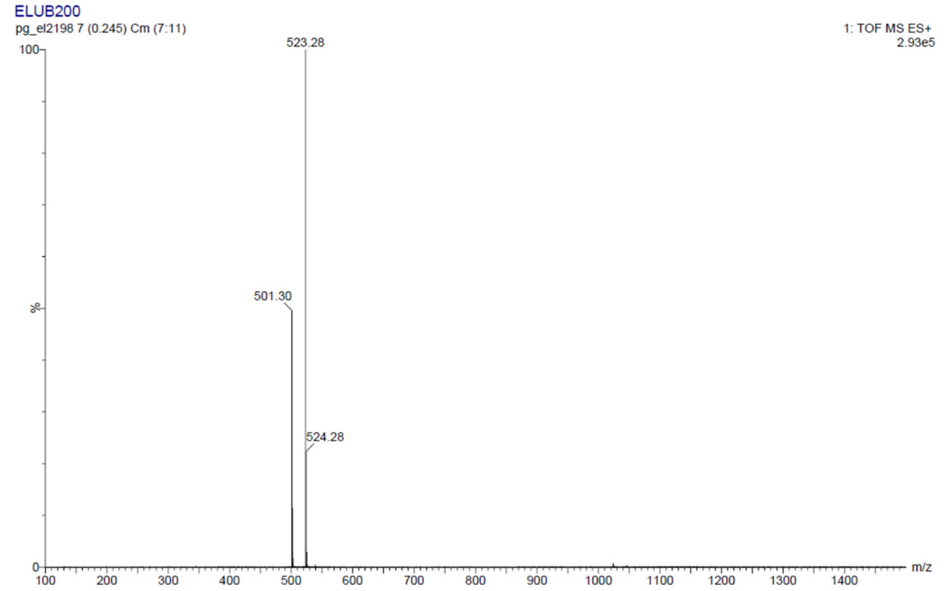

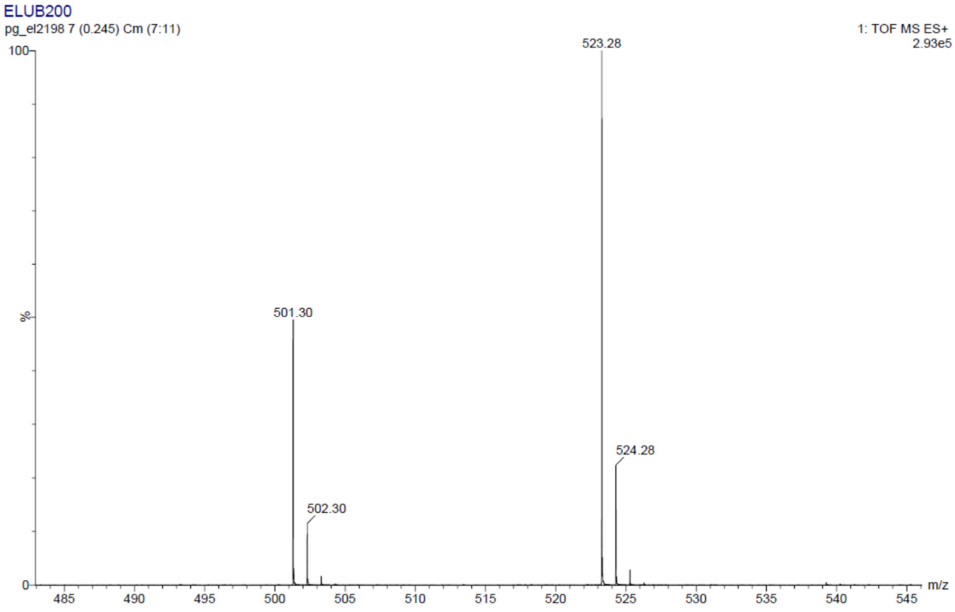

Figure S11. MS (ESI) spectrum of ***t*-Bu-19-*p*-OH**

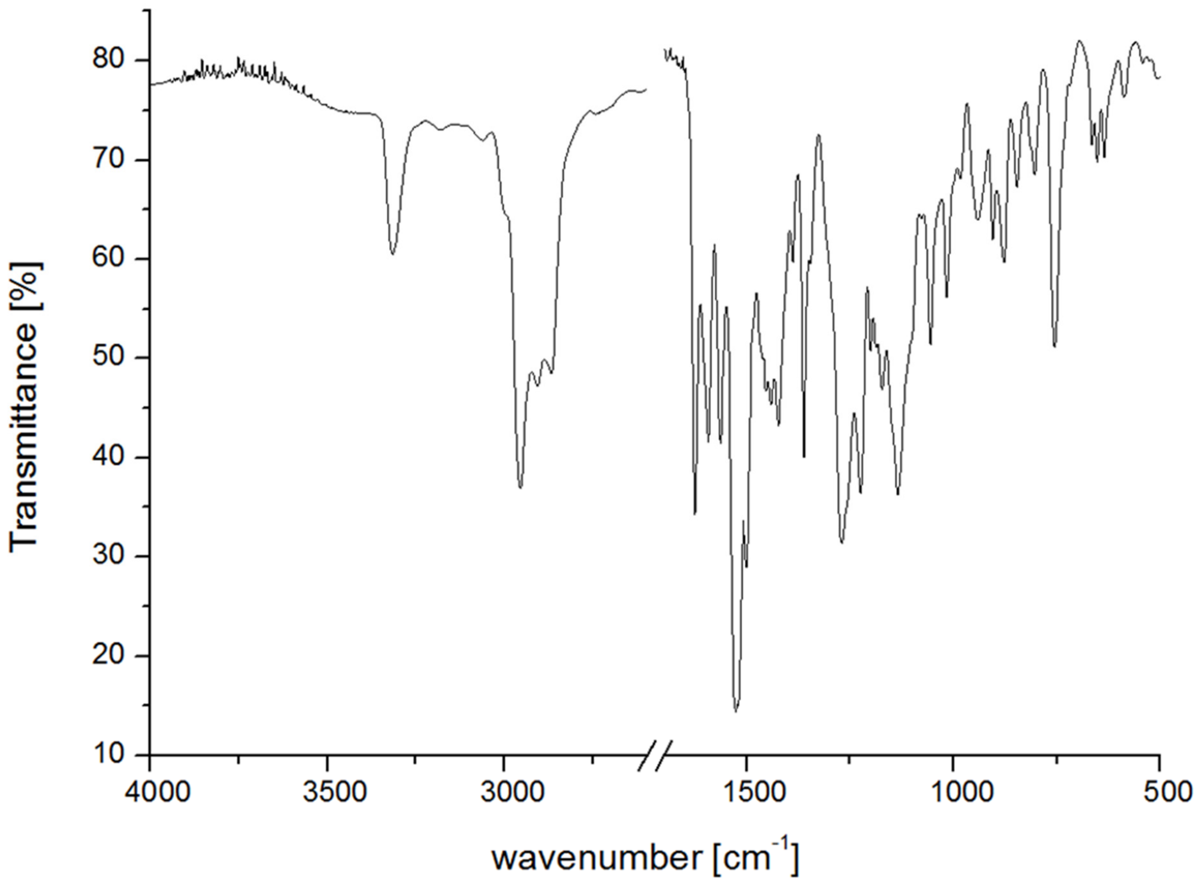

Figure S1m. FTIR spectrum (film) of ***t*-Bu-19-*p*-OH**

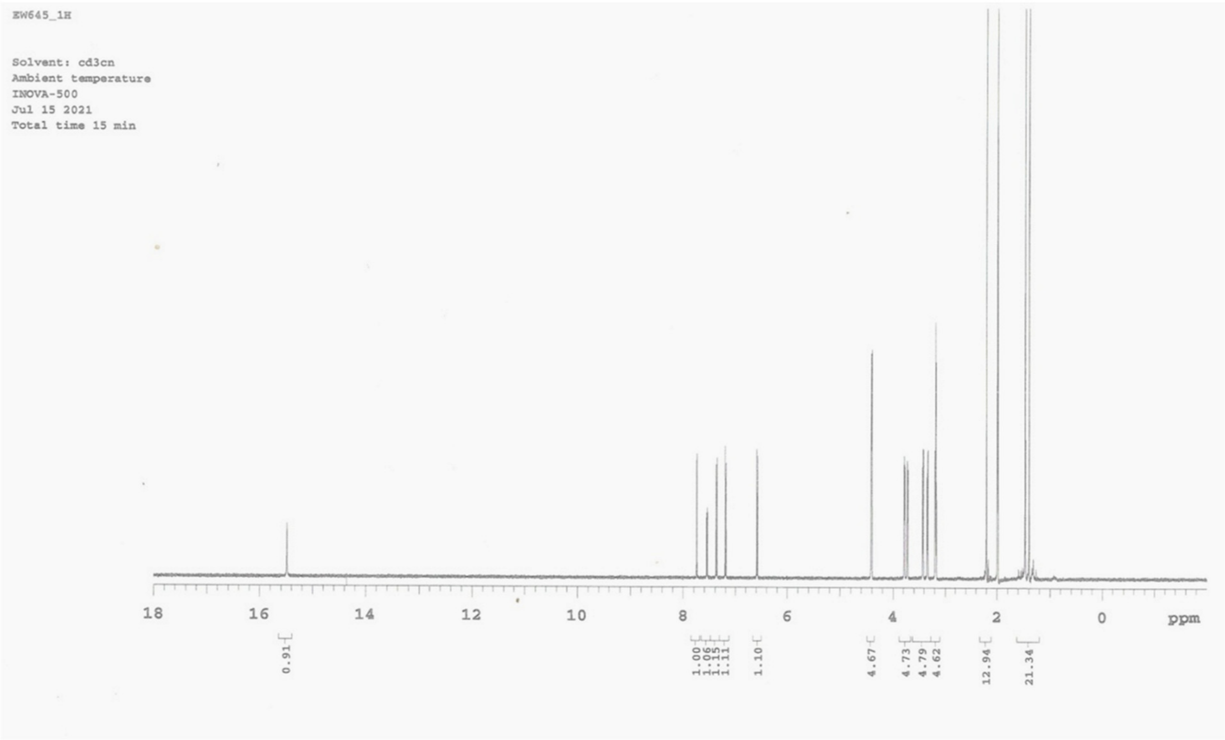

Figure S2a. ^{1}H NMR spectrum of ***t*-Bu-19-*o*-OH** (6.3 ×10^{-3} M) in acetonitrile-d_3

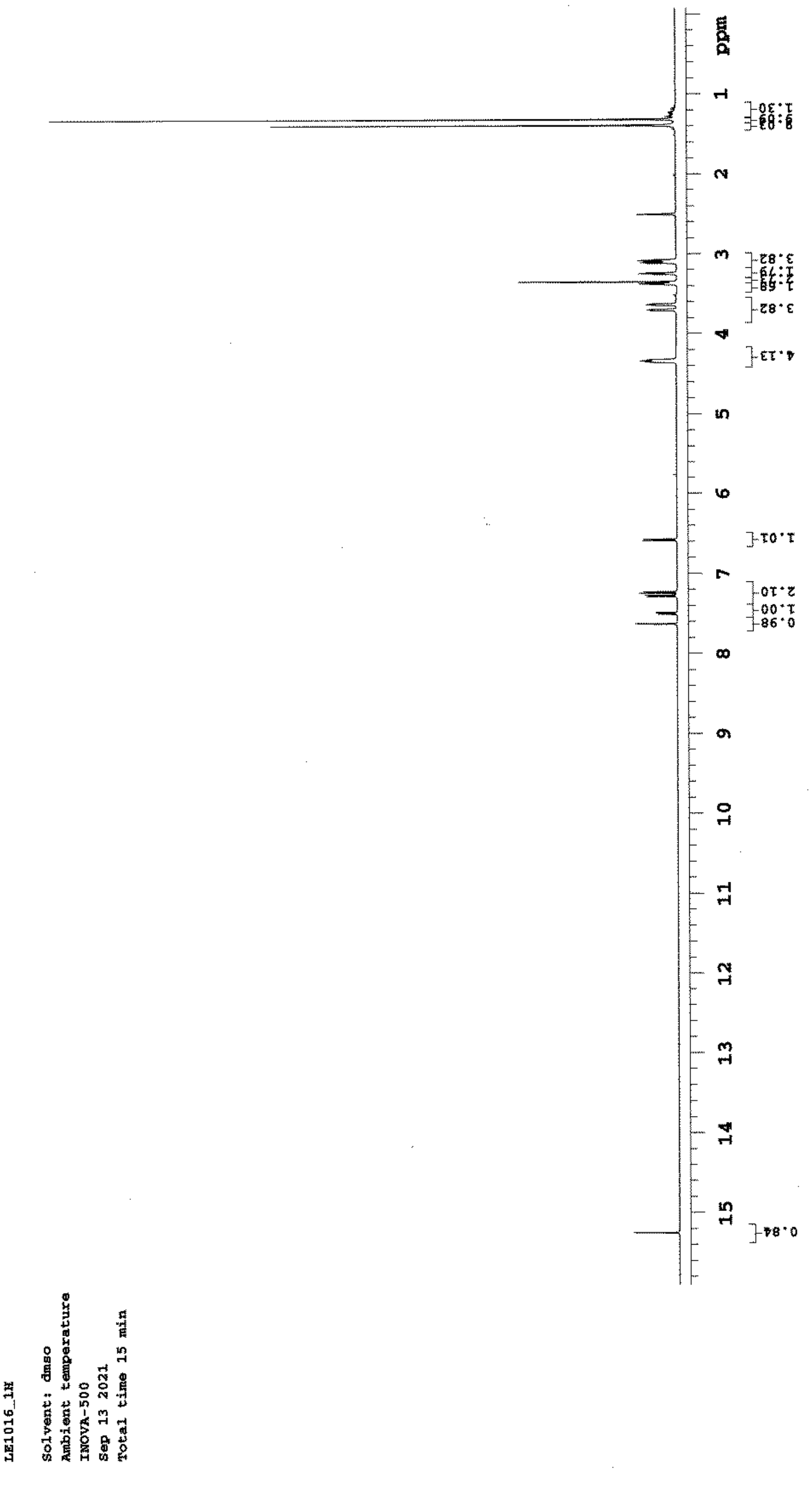

Figure S2b. ^{1}H NMR spectrum of ***t*-Bu-19-*o*-OH** in DMSO-d_6

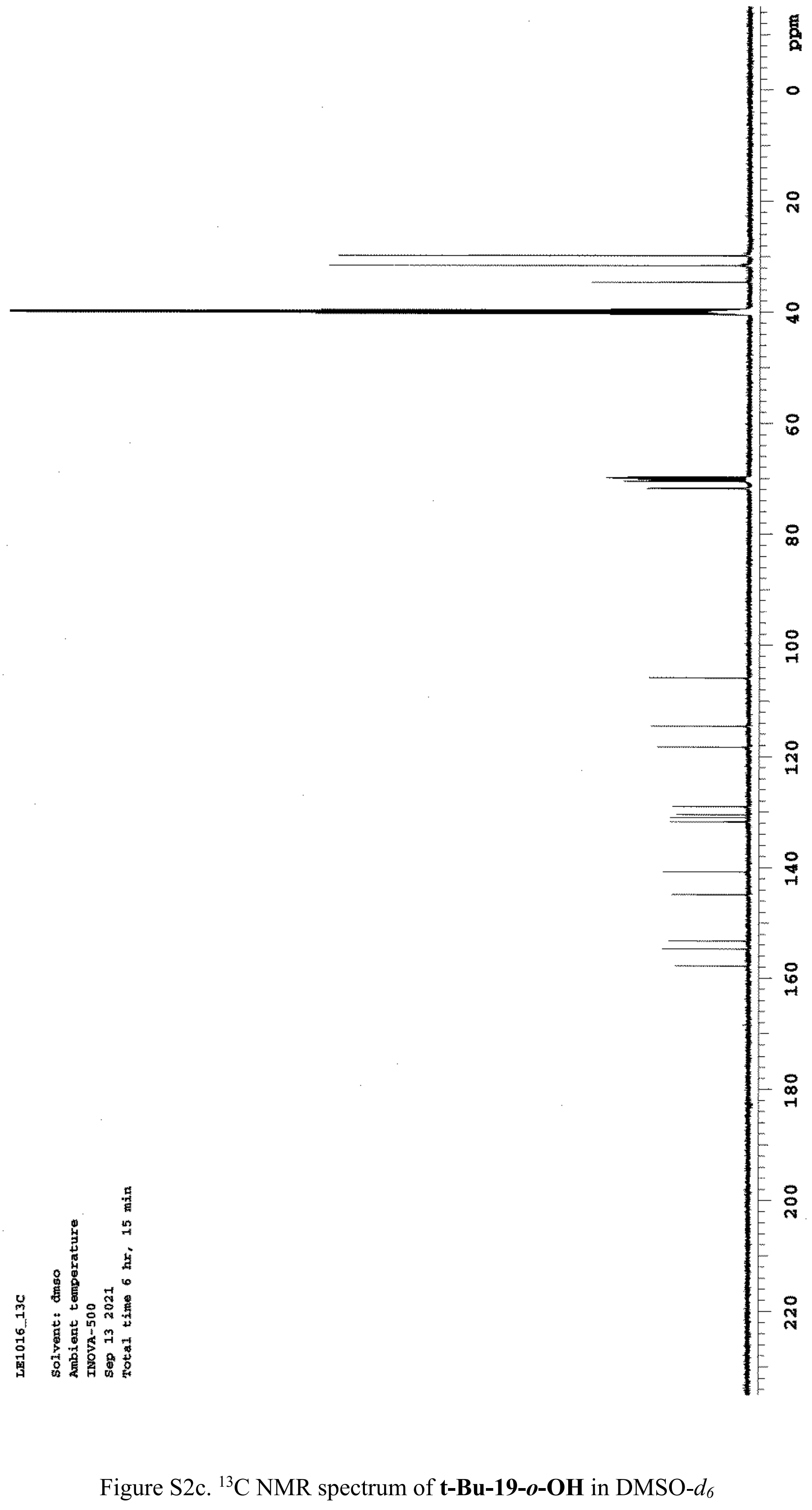

Figure S2c. ^{13}C NMR spectrum of **t-Bu-19-*o*-OH** in DMSO-d_6

Single Mass Analysis
Tolerance = 3.0 mDa / DBE: min = -1.5, max = 100.0
Element prediction: Off
Number of isotope peaks used for i-FIT = 3
Monoisotopic Mass, Even Electron Ions
130 formula(e) evaluated with 1 results within limits (all results (up to 1000) for each mass)
Elements Used:
C: 0-100 H: 0-200 N: 0-2 O: 0-6 Na: 1-1

Mass	Calc. Mass	mDa	PPM	DBE	Formula	i-FIT	i-FIT Norm	Fit Conf %	C	H	N	O	Na
523.2785	523.2784	0.1	0.2	9.5	C28 H40 N2 O6 Na	143.0	n/a	n/a	28	40	2	6	1

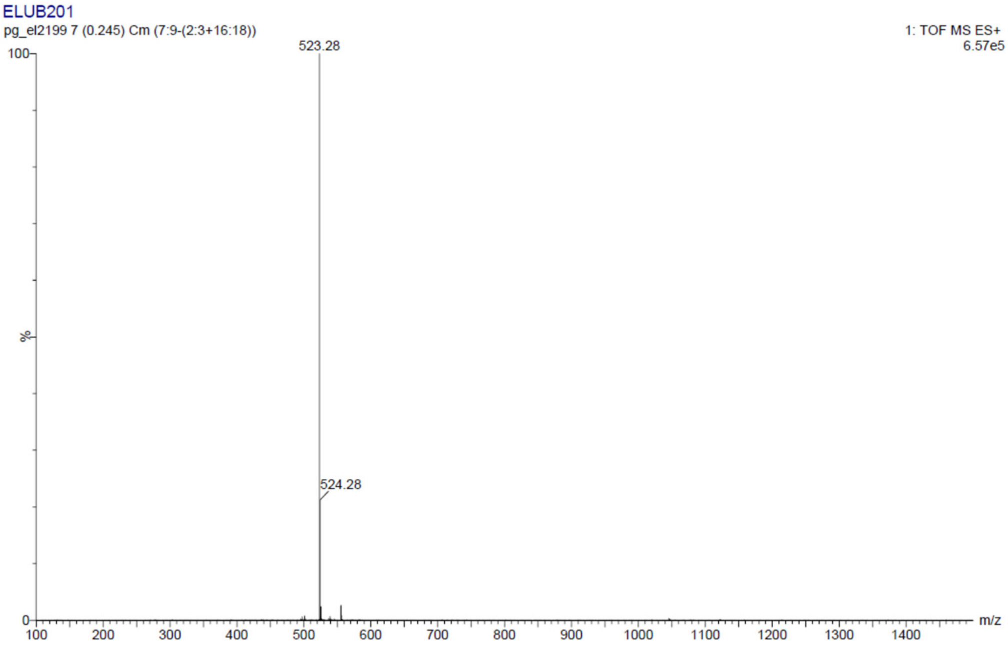

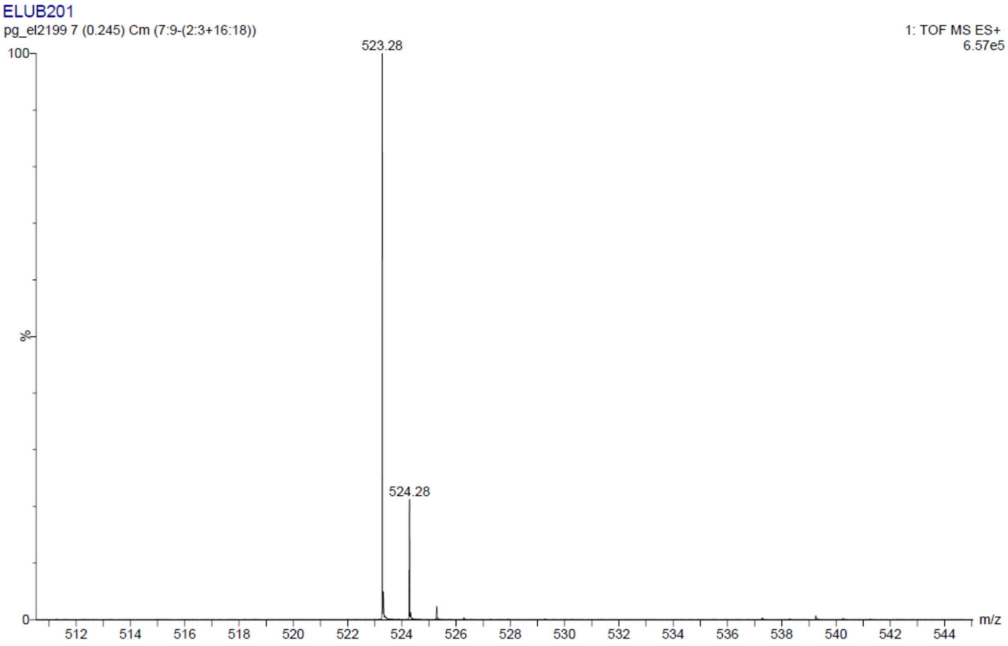

Figure S2d. MS (ESI) spectrum of ***t*-Bu-19-*o*-OH**

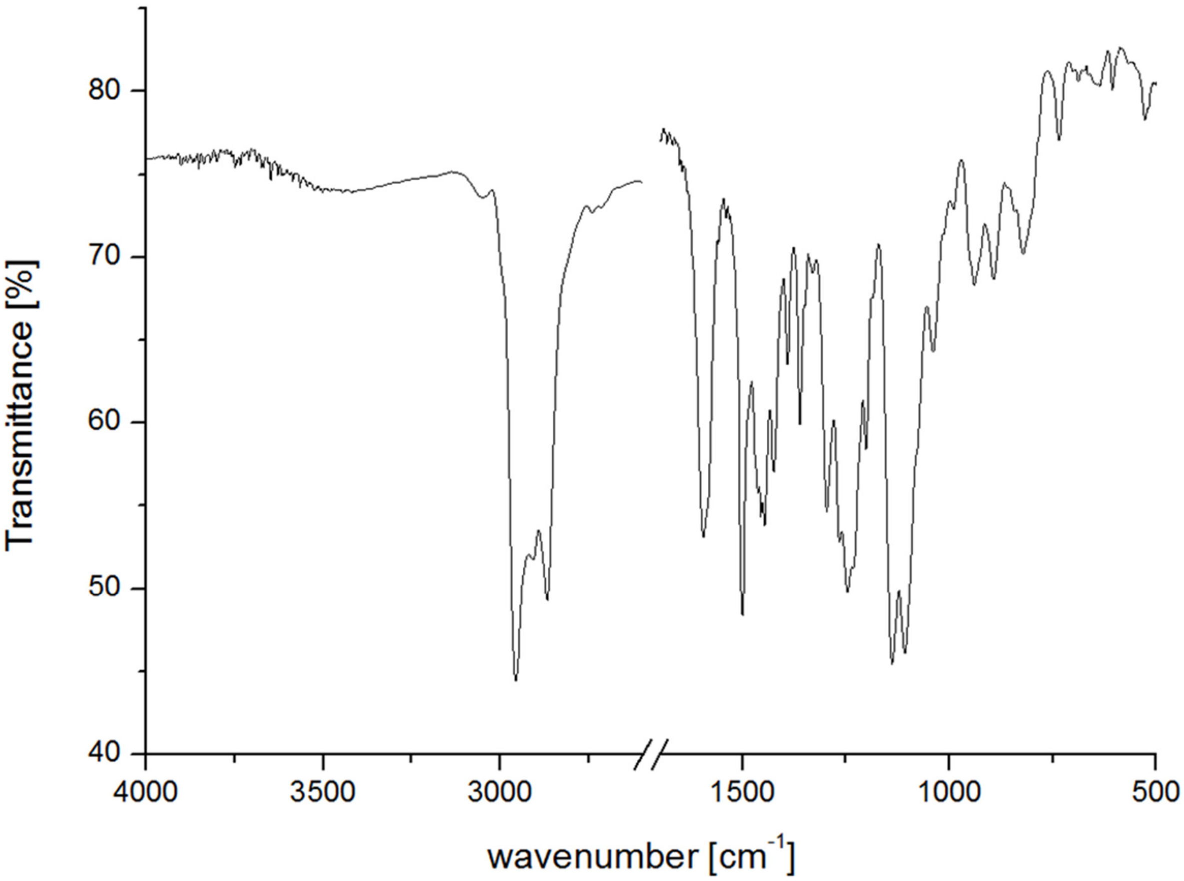

Figure S2e. FTIR spectrum (film) of ***t*-Bu-19-*o*-OH**

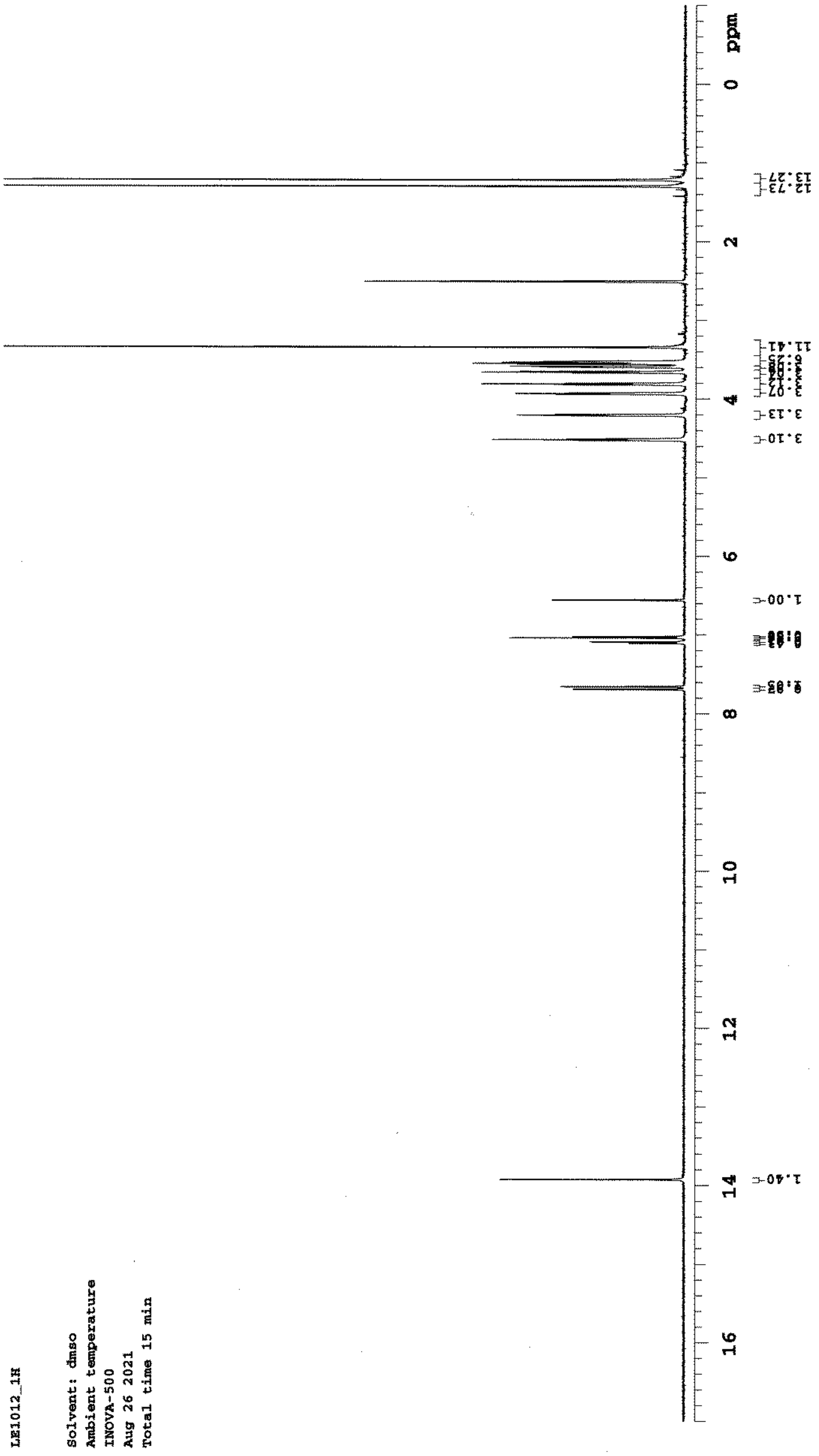

Figure S3a. ^{1}H NMR spectrum of ***t*-Bu-20-ester** in DMSO-d_6

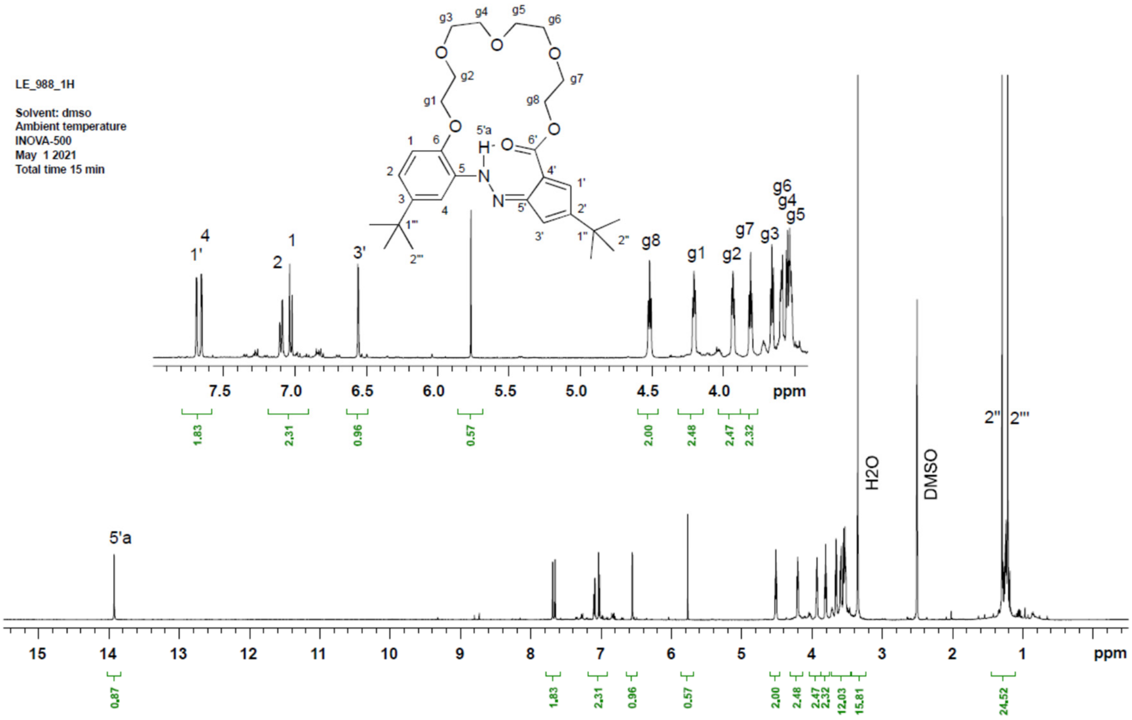

Figure S3b. ^{1}H NMR spectrum of ***t*-Bu-20-ester** in DMSO-d_6

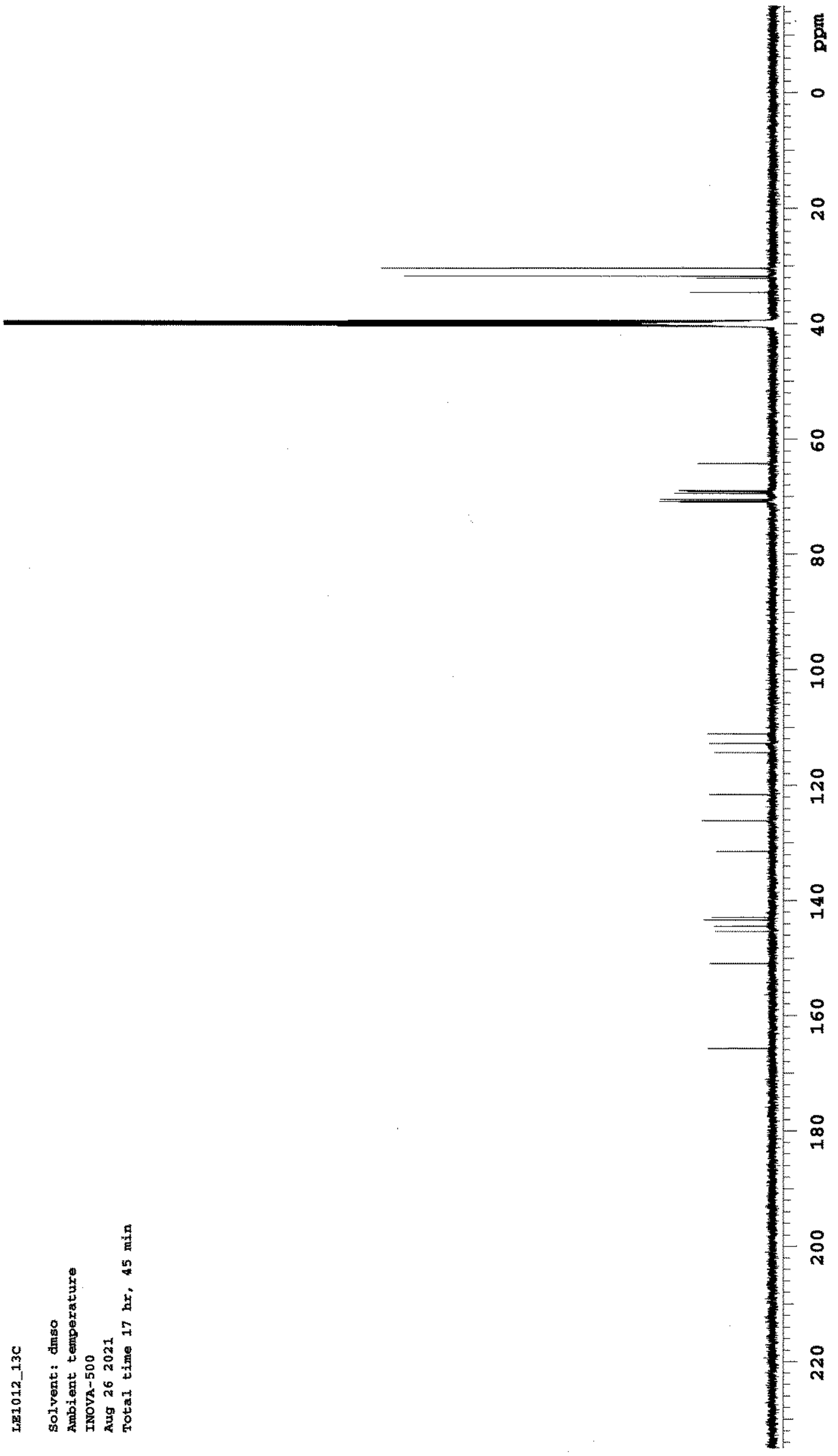

Figure S3c. ^{13}C NMR spectrum of ***t*-Bu-20-ester** in DMSO-d_6

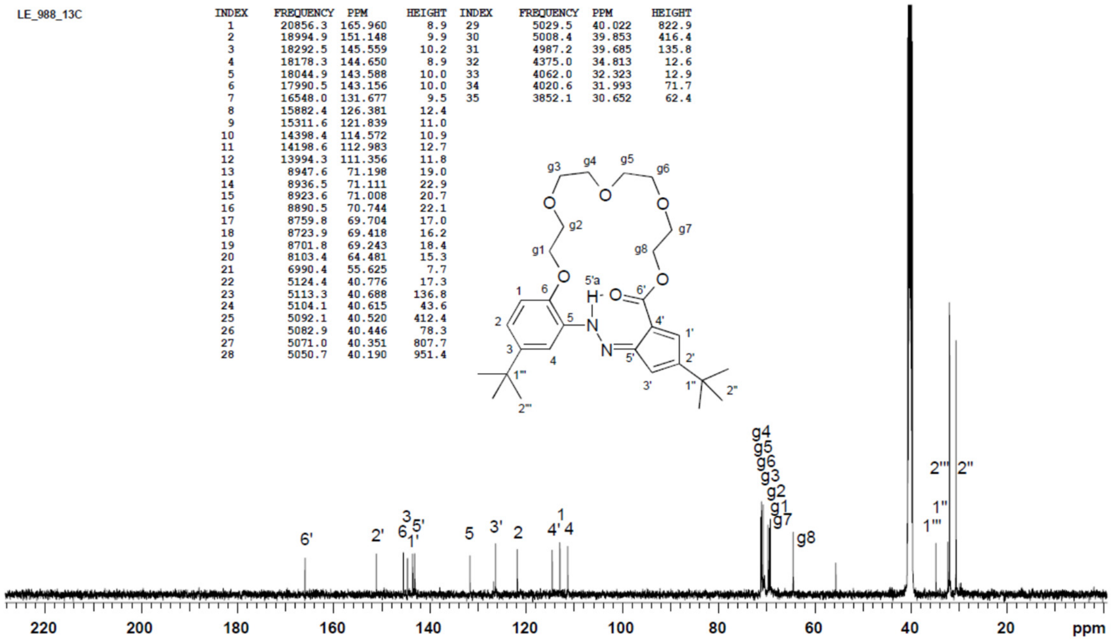

Figure S3d. ^{13}C NMR spectrum of ***t*-Bu-20-ester** in DMSO-d_6

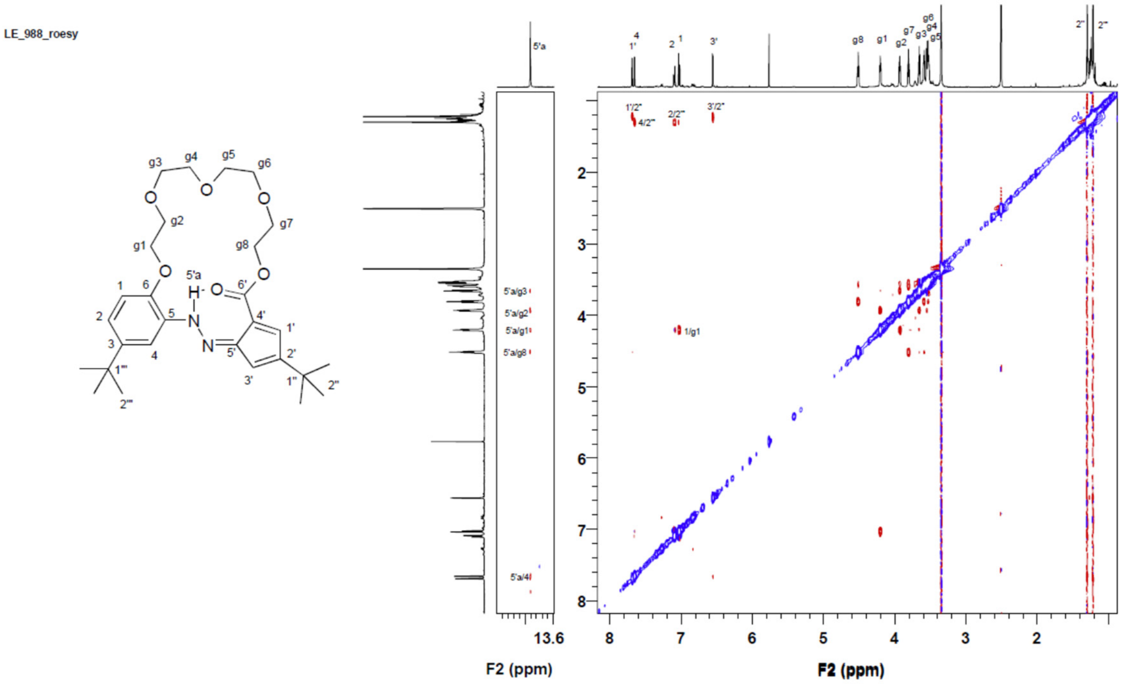

Figure S3e. ROESY spectrum of ***t*-Bu-20-ester** in DMSO-d_6

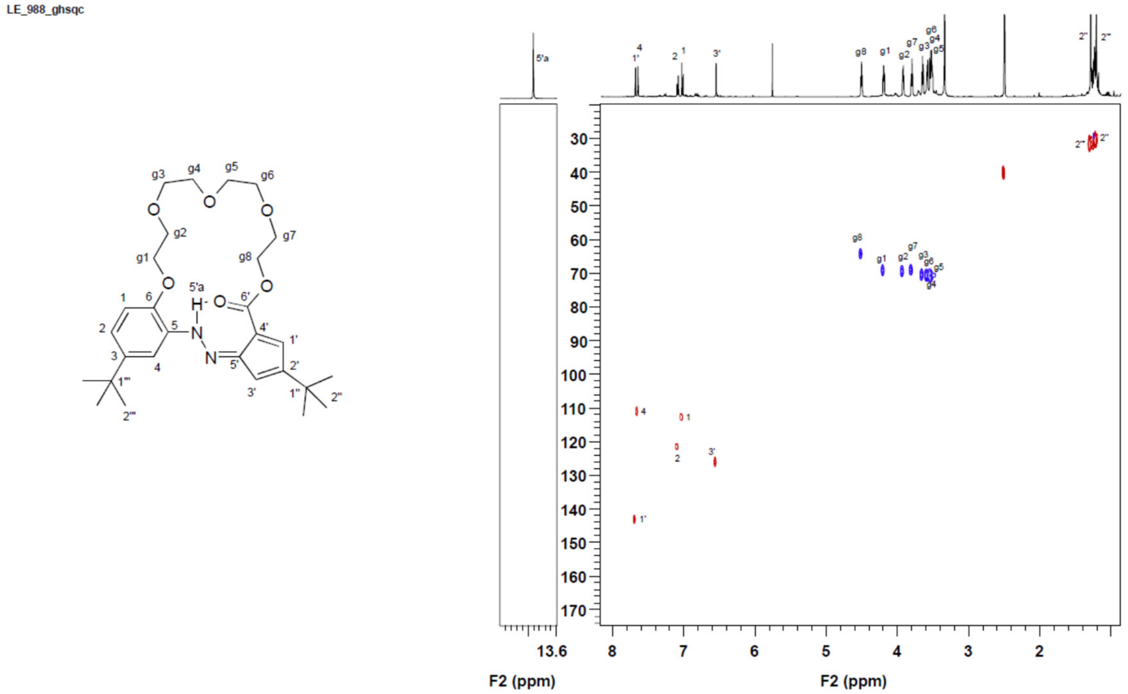

Figure S3f. GHSQC spectrum of ***t*-Bu-20-ester** in DMSO-d_6

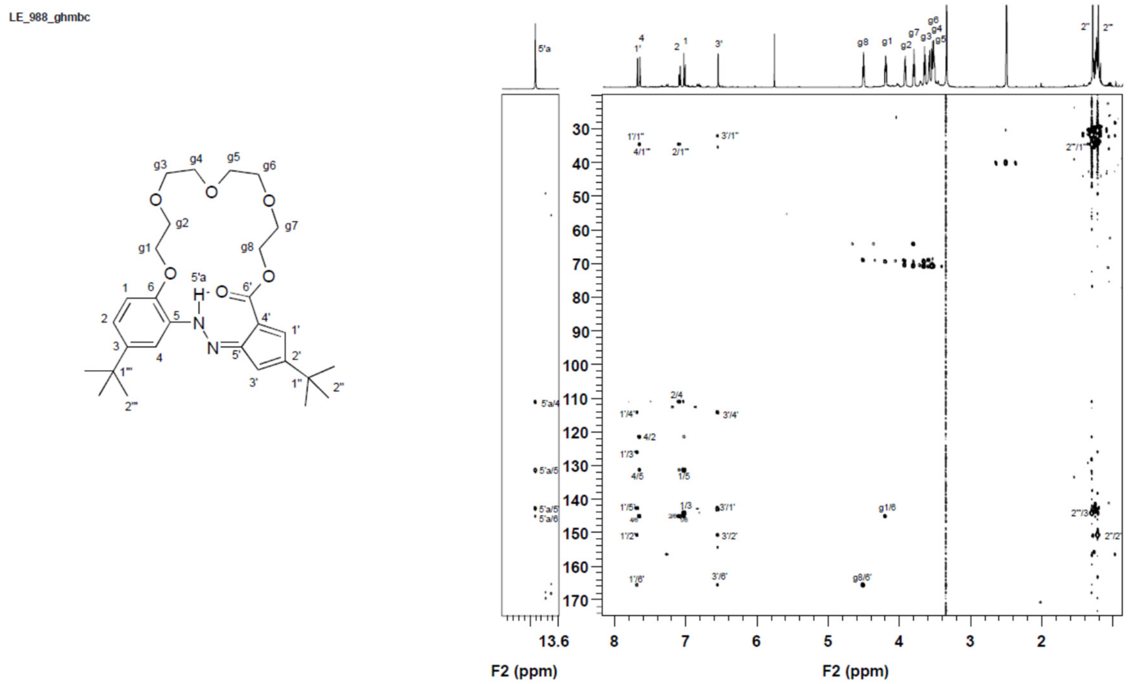

Figure S3g. GHMBC spectrum of ***t*-Bu-20-ester** in DMSO-d_6

Single Mass Analysis
Tolerance = 3.0 mDa / DBE: min = -1.5, max = 100.0
Element prediction: Off
Number of isotope peaks used for i-FIT = 3
Monoisotopic Mass, Even Electron Ions
130 formula(e) evaluated with 1 results within limits (all results (up to 1000) for each mass)
Elements Used:
C: 0-100 H: 0-200 N: 0-2 O: 0-6 Na: 1-1

Mass	Calc. Mass	mDa	PPM	DBE	Formula	i-FIT	i-FIT Norm	Fit Conf %	C	H	N	O	Na
523.2787	523.2784	0.3	0.6	9.5	C28 H40 N2 O6 Na	110.0	n/a	n/a	28	40	2	6	1

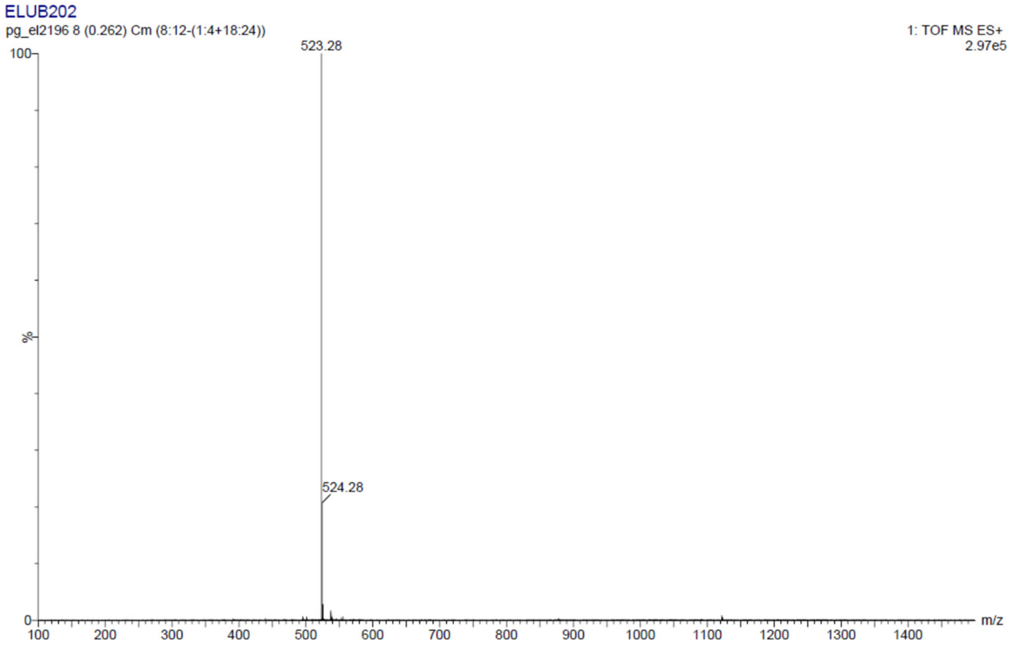

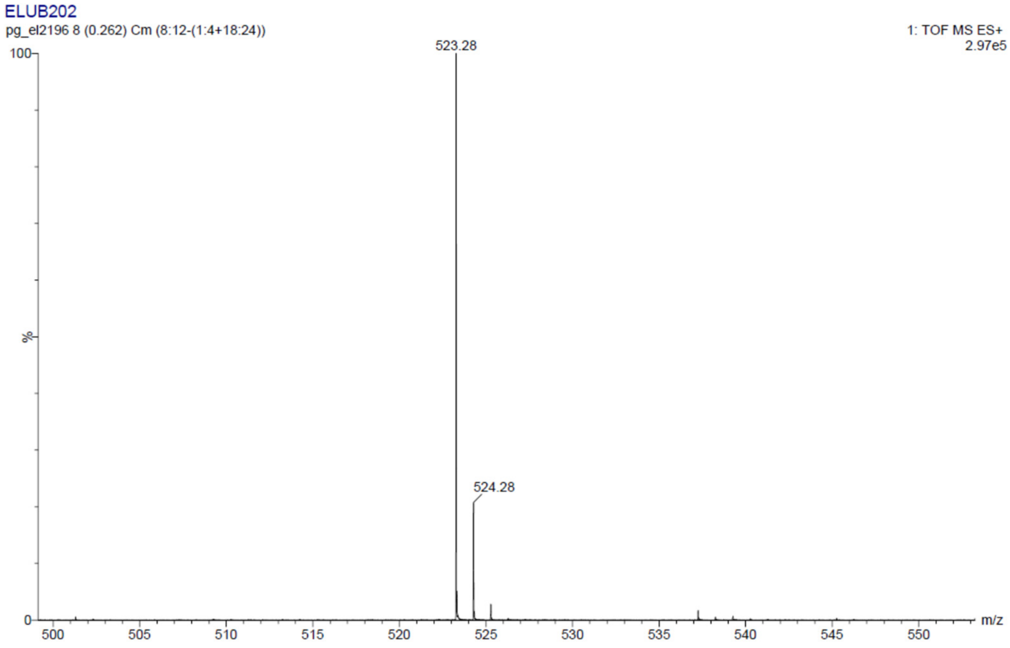

Figure S3h. MS (ESI) spectrum of ***t*-Bu-20-ester**

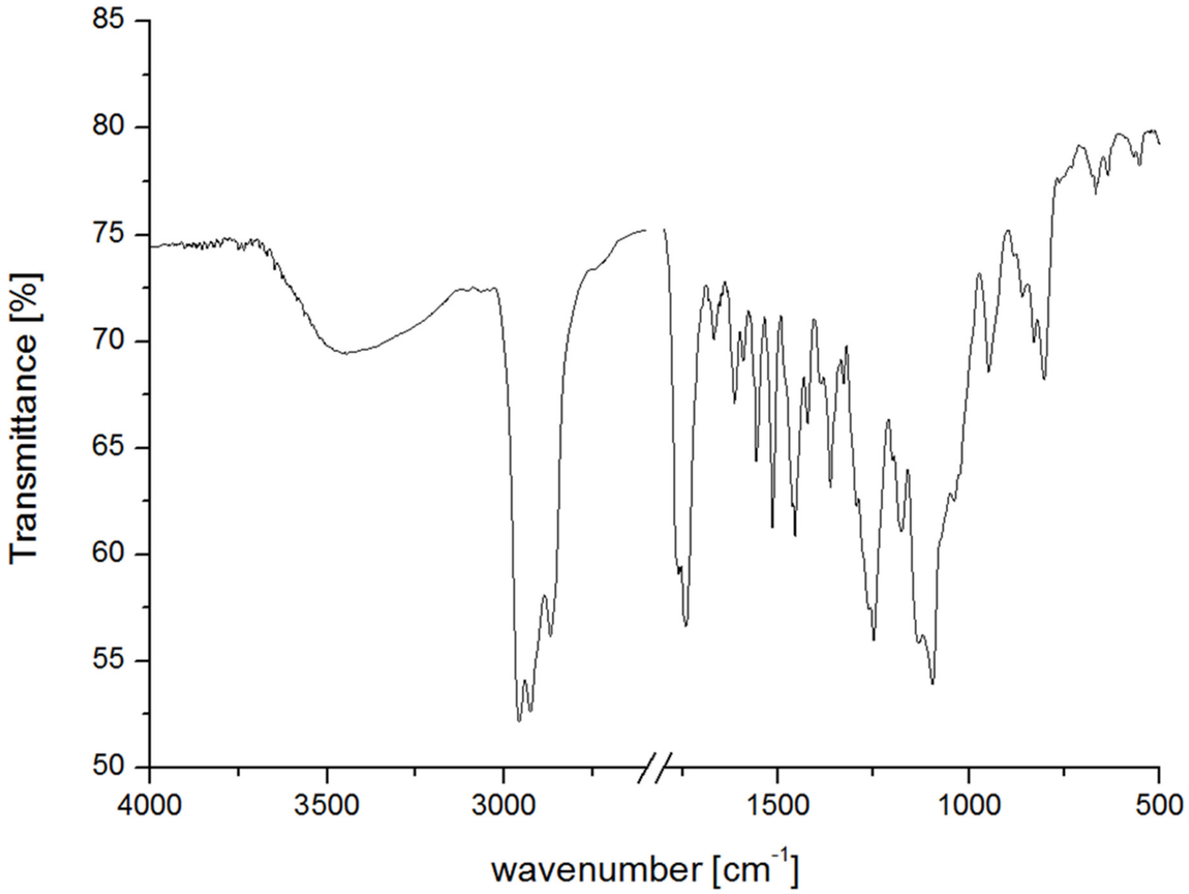

Figure S3i. FTIR spectrum (film) of ***t*-Bu-20-ester**

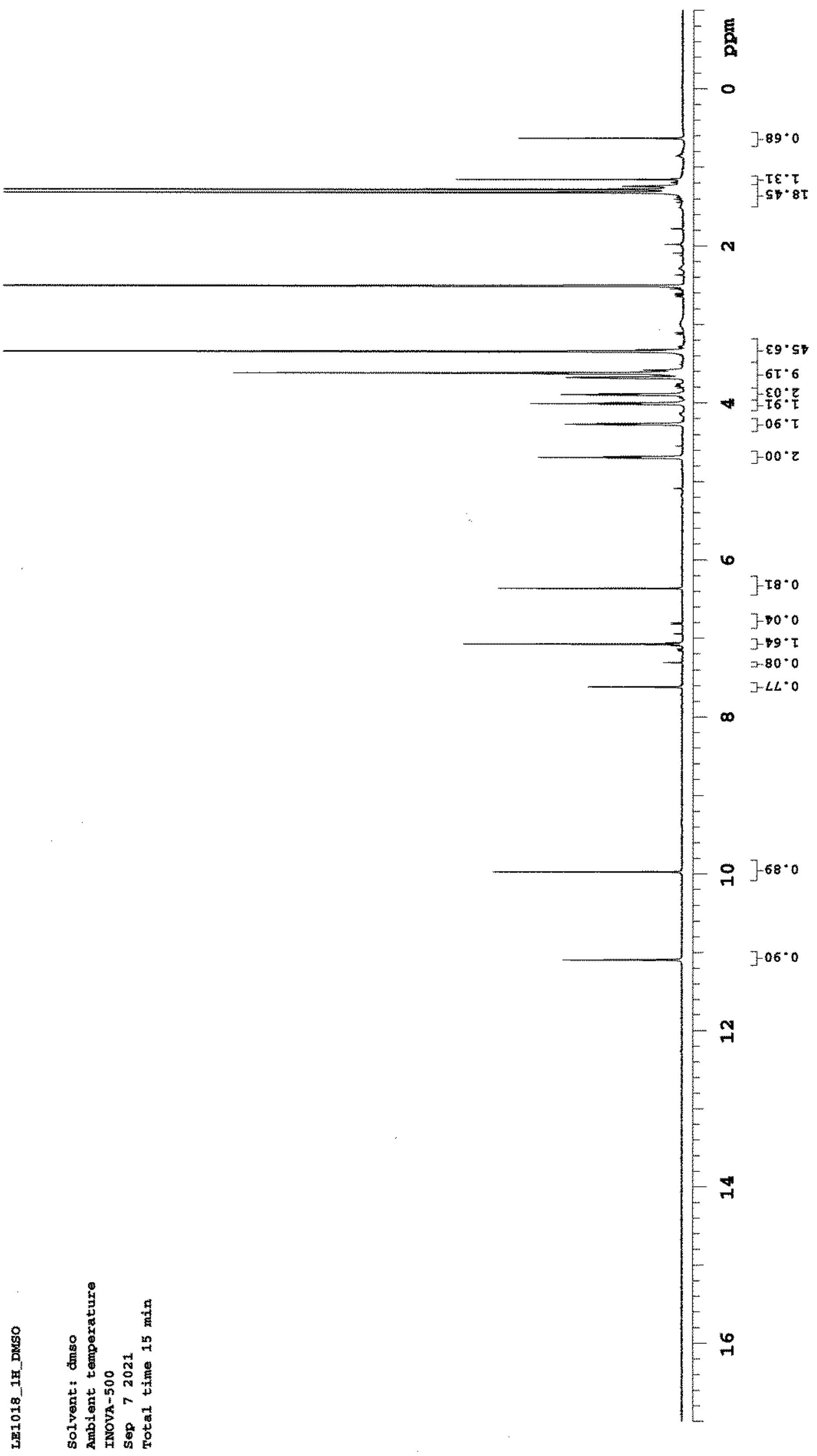

Figure S4a. 1H NMR spectrum of ***t*-Bu-19-al** in DMSO-d_6

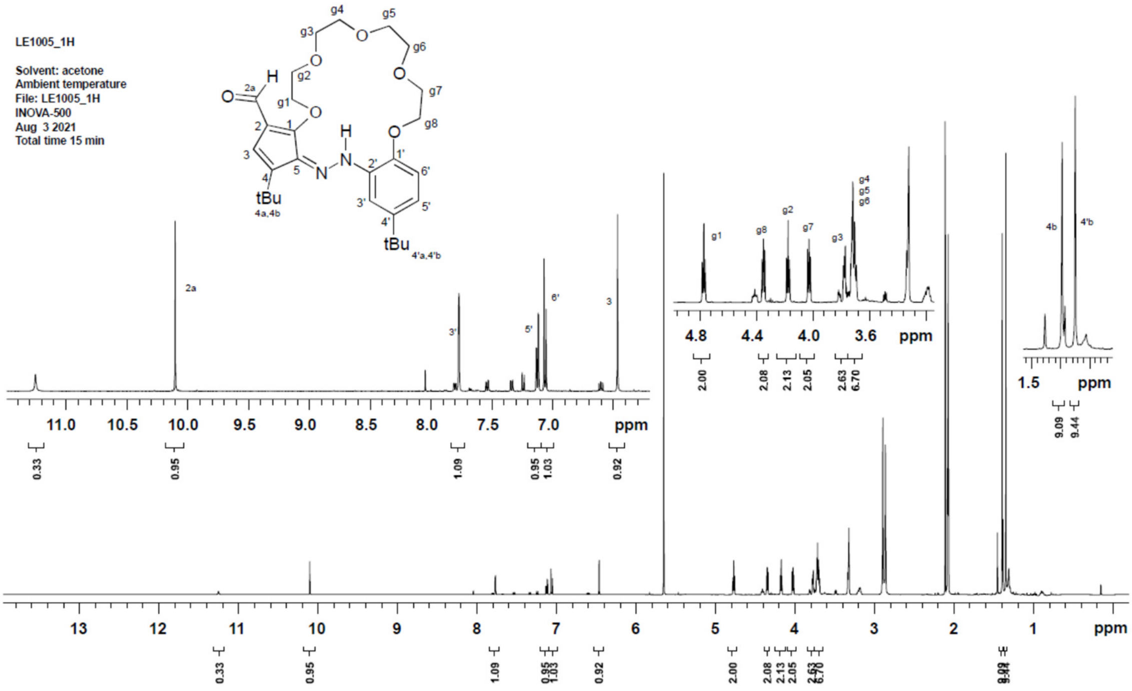

Figure S4b. ^{1}H NMR spectrum of ***t*-Bu-19-al** in acetone-d_6

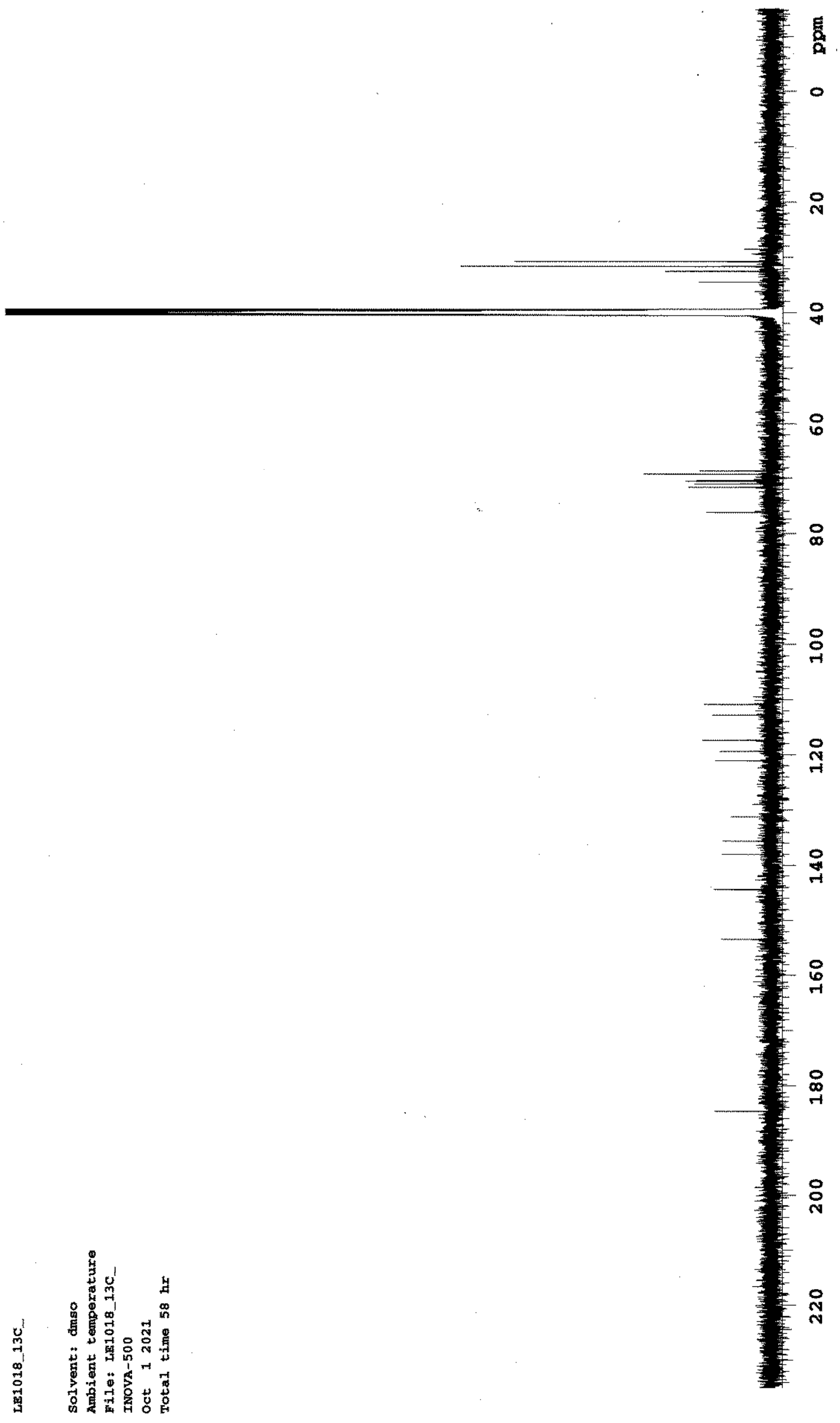

Figure S4c. ^{13}C NMR spectrum of ***t*-Bu-19-al** in DMSO-d_6

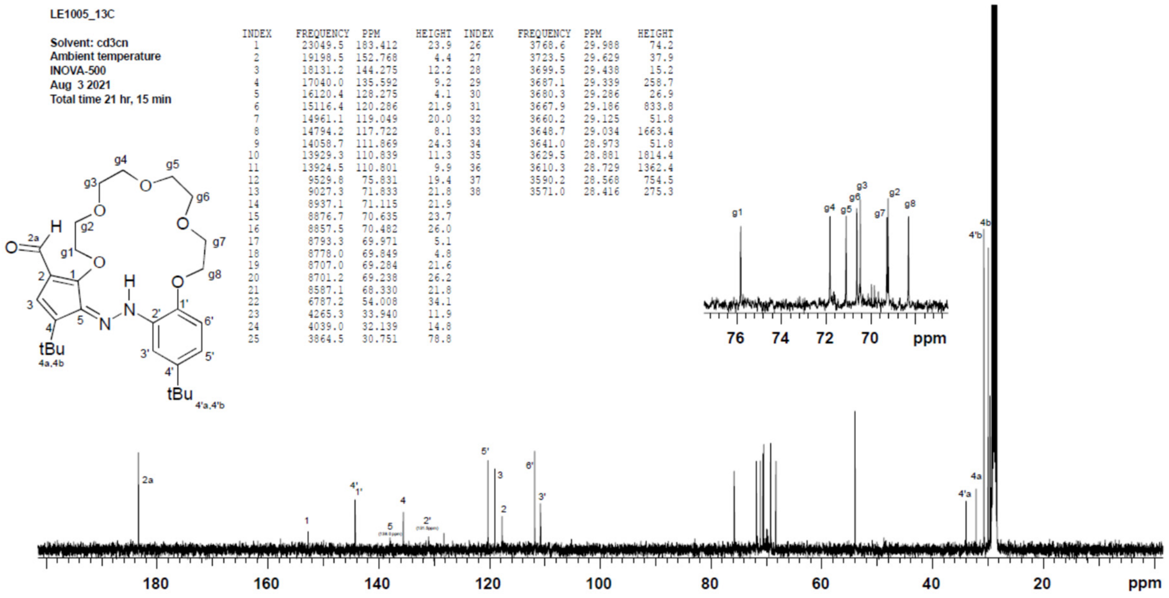

Figure S4d. ^{13}C NMR spectrum of ***t*-Bu-19-al** in acetone-d_6

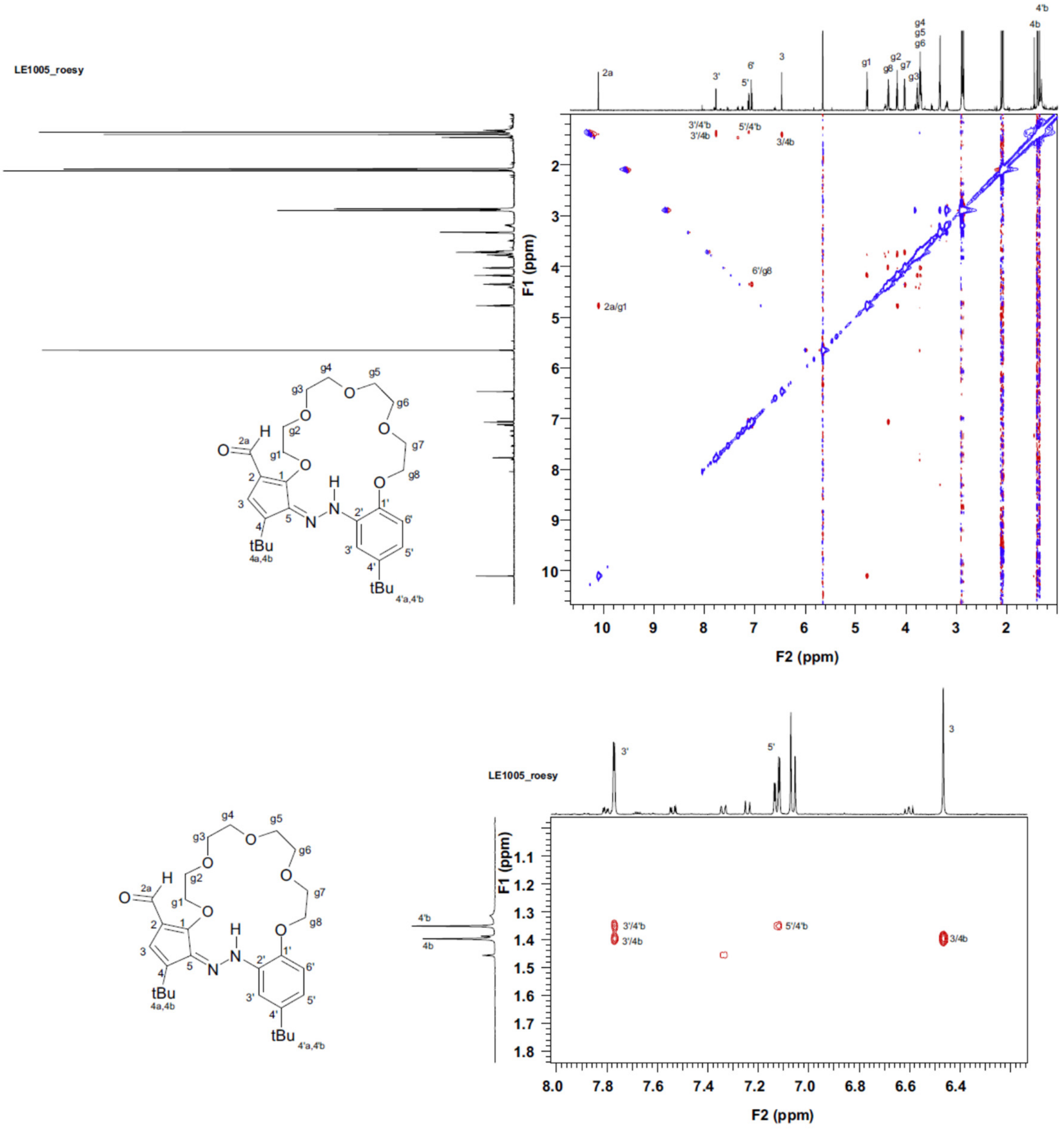

Figure S4e. ROESY spectrum of ***t*-Bu-19-al** in acetone-d_6

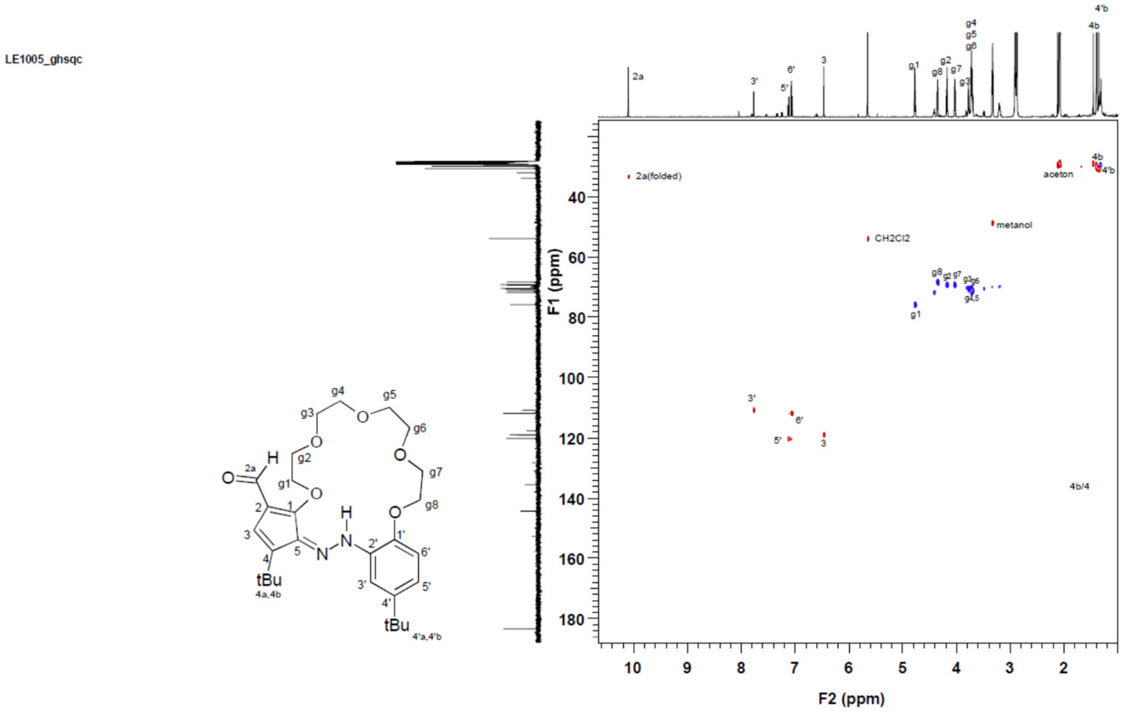

Figure S4f. gHSQC spectrum of ***t*-Bu-19-al** in acetone-d_6

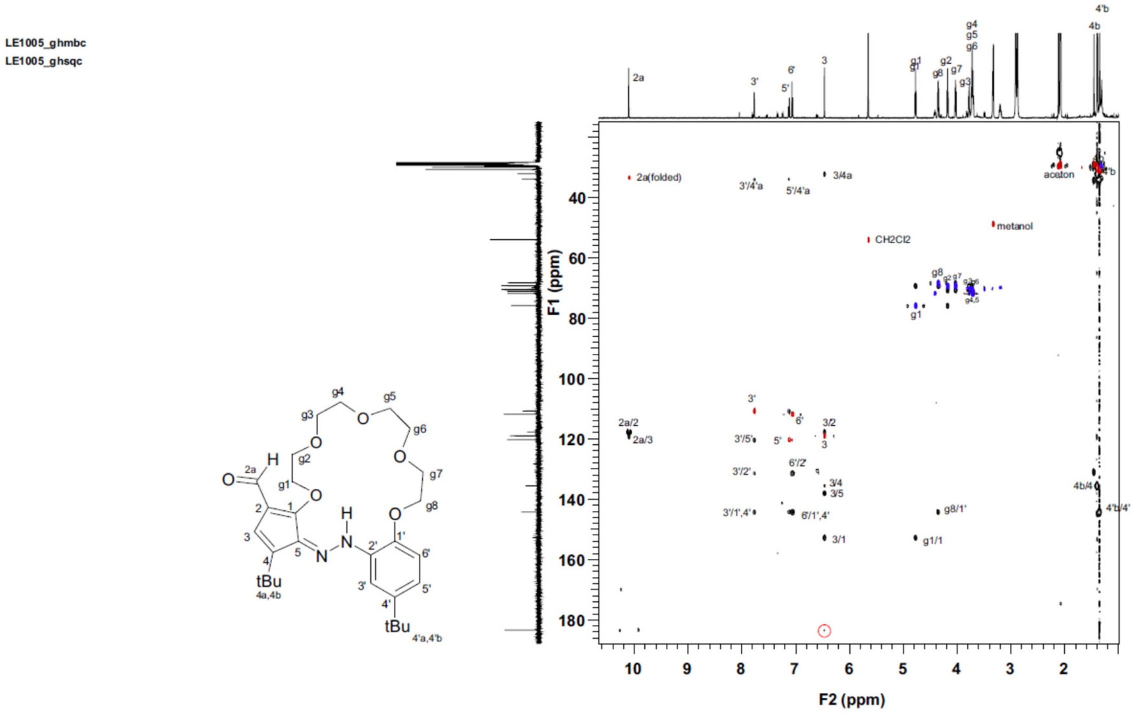

Figure S4g. gHMBC spectrum of ***t*-Bu-19-al** in acetone-d_6

Single Mass Analysis
Tolerance = 3.0 mDa / DBE: min = -1.5, max = 100.0
Element prediction: Off
Number of isotope peaks used for i-FIT = 3
Monoisotopic Mass, Even Electron Ions
133 formula(e) evaluated with 1 results within limits (all results (up to 1000) for each mass)
Elements Used:
C: 0-100 H: 0-200 N: 0-2 O: 0-6

Mass	Calc. Mass	mDa	PPM	DBE	Formula	i-FIT	i-FIT Norm	Fit Conf %	C	H	N	O
499.2800	499.2808	-0.8	-1.6	10.5	C28 H39 N2 O6	1106.2	n/a	n/a	28	39	2	6

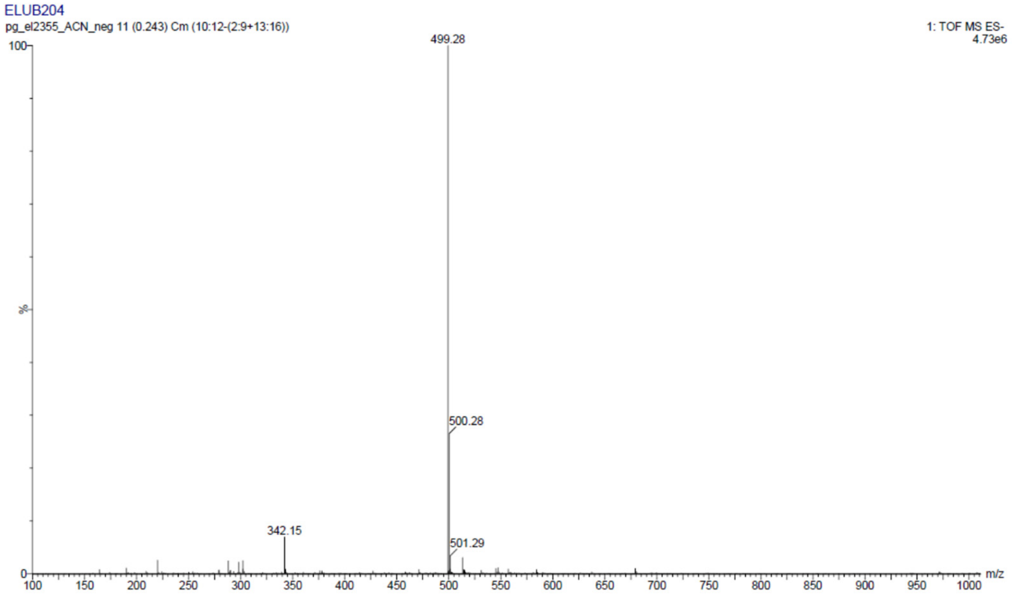

Figure S4h. MS (ESI) spectrum of ***t*-Bu-19-al**

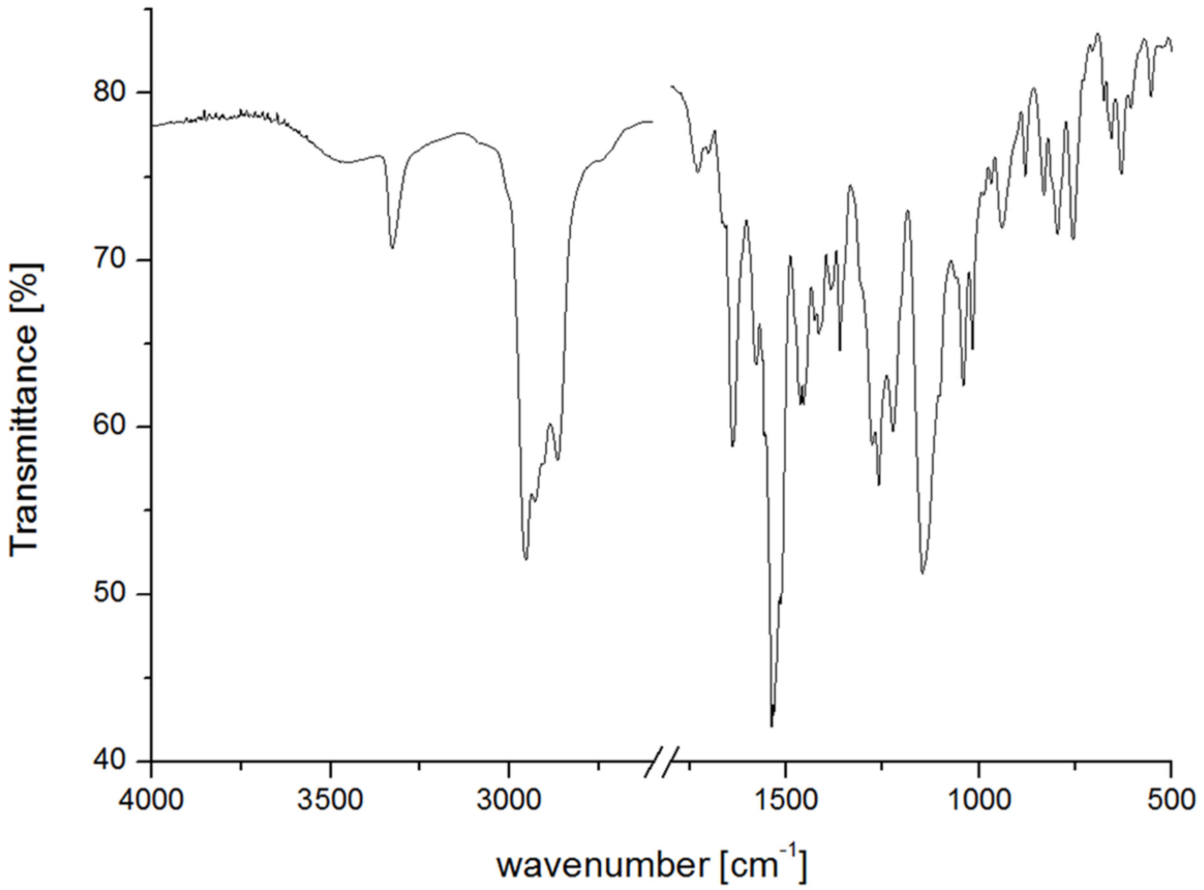

Figure S4i. FTIR spectrum (film) of ***t*-Bu-19-al**

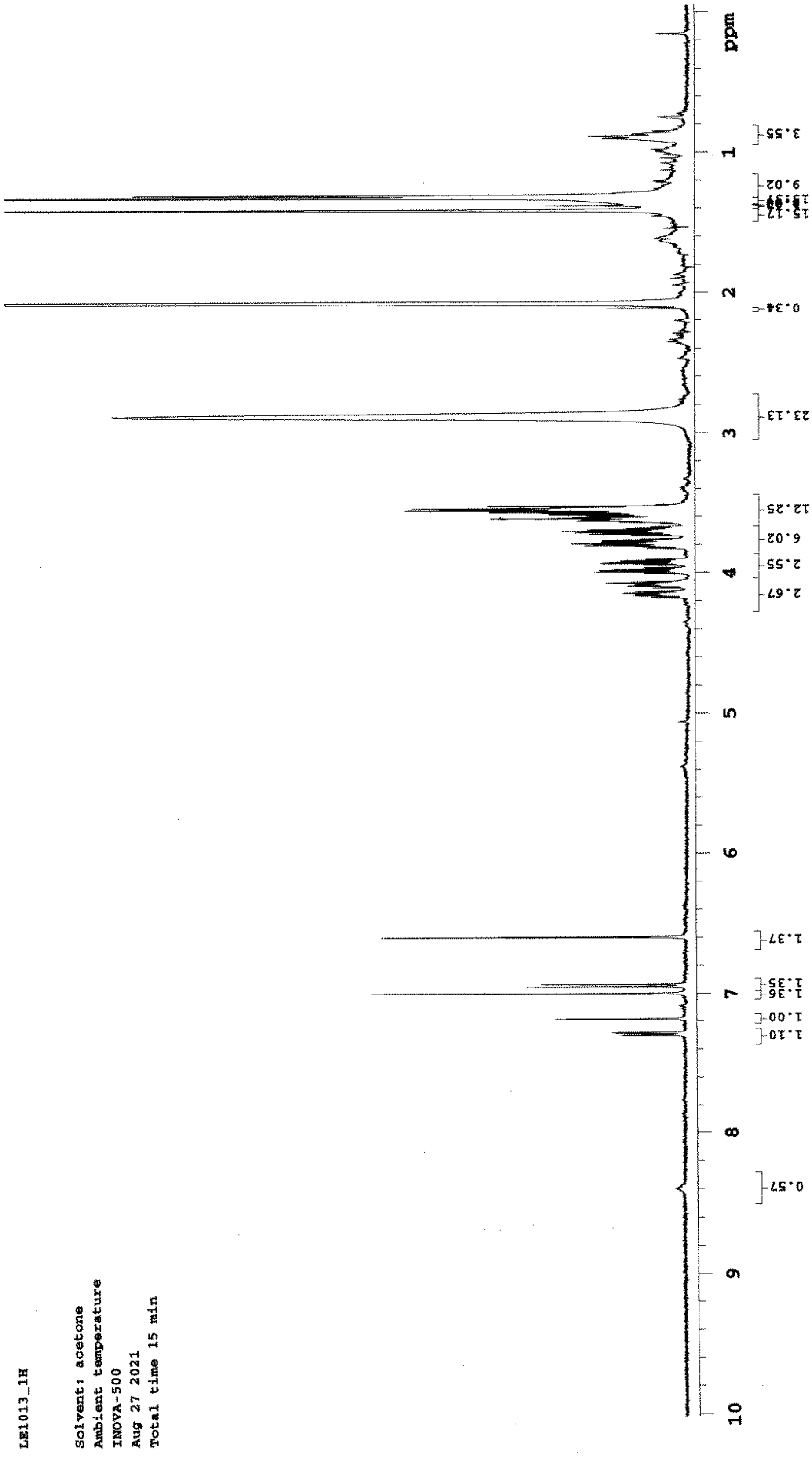

Figure S5a. ^{1}H NMR spectrum of ***t*-Bu-17-*p*-OH** in acetone-d_6

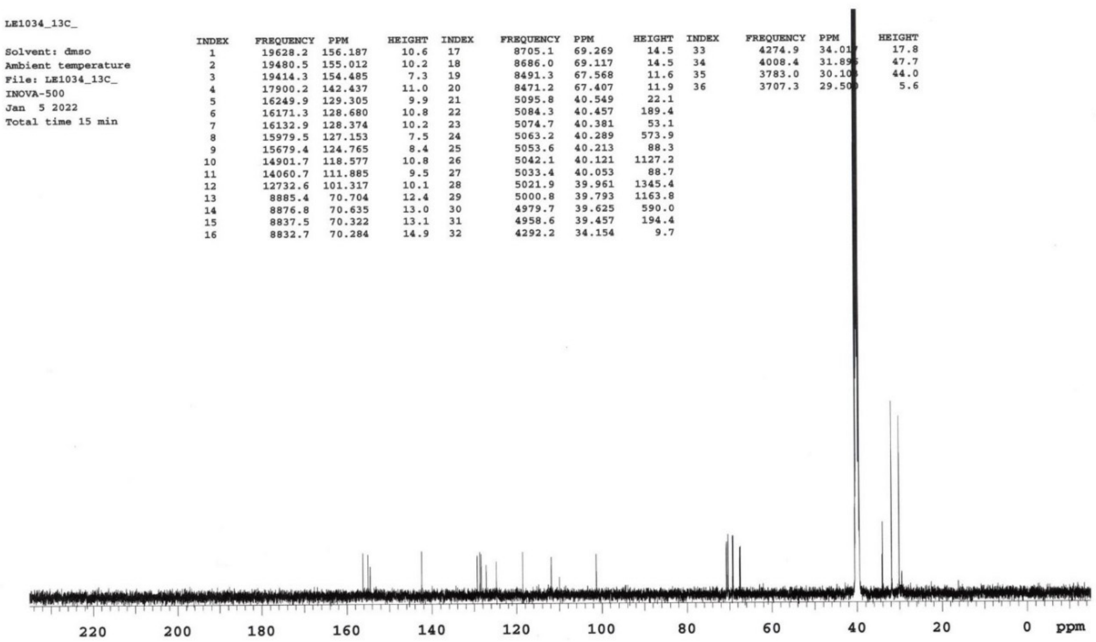

Figure S5b. ^{13}C NMR spectrum of ***t*-Bu-17-*p*-OH** in DMSO-d_6

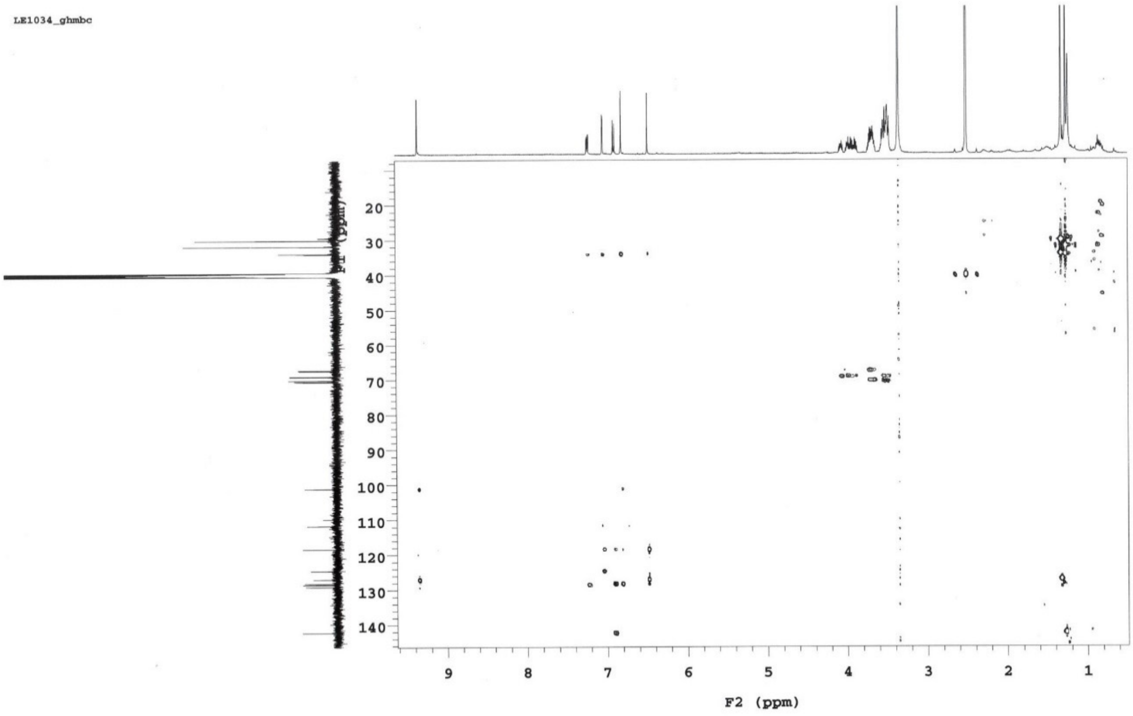

Figure S5c. gHMBC spectrum of ***t*-Bu-17-*p*-OH** in DMSO-d_6

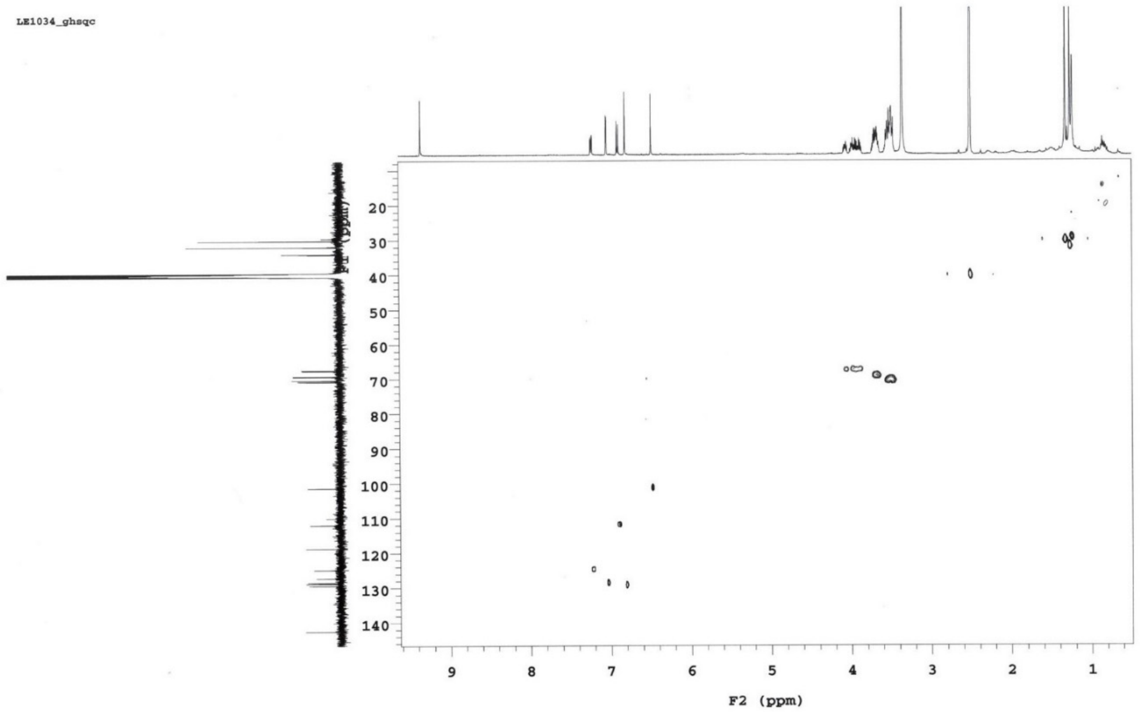

Figure S5d.gHSQC spectrum of ***t*-Bu-17-*p*-OH** in DMSO-d_6

Single Mass Analysis
Tolerance = 3.0 mDa / DBE: min = -1.5, max = 50.0
Element prediction: Off
Number of isotope peaks used for i-FIT = 3
Monoisotopic Mass, Even Electron Ions
87 formula(e) evaluated with 2 results within limits (all results (up to 1000) for each mass)
Elements Used:
C: 0-100 H: 0-200 O: 0-6 Na: 0-1

Mass	Calc. Mass	mDa	PPM	DBE	Formula	i-FIT	i-FIT Norm	Fit Conf %	C	H	O	Na
495.2722	495.2723	-0.1	-0.2	8.5	C28 H40 O6 Na	244.1	0.033	96.74	28	40	6	1
	495.2747	-2.5	-5.0	11.5	C30 H39 O6	247.4	3.423	3.26	30	39	6	

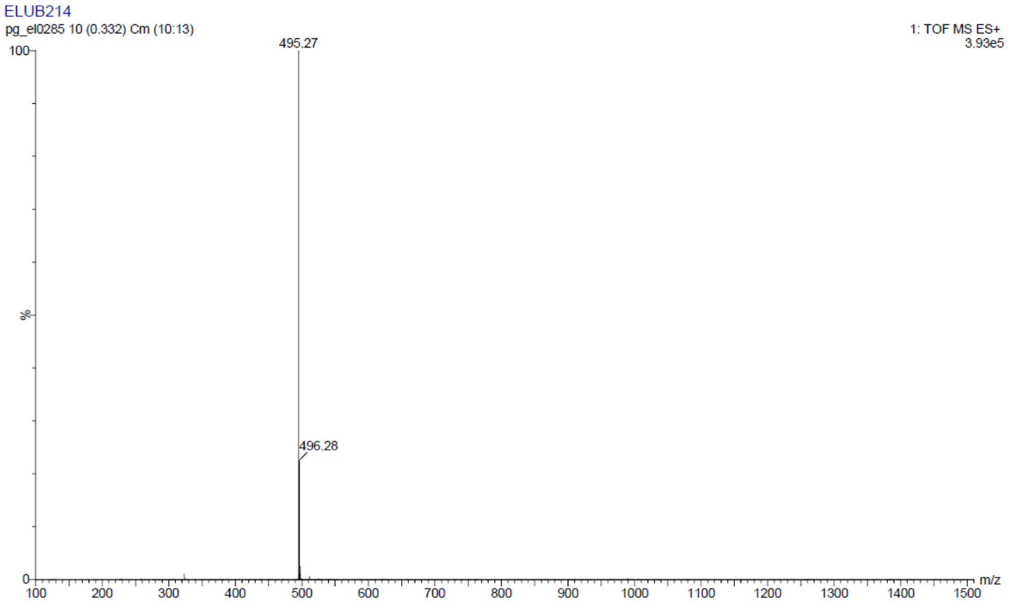

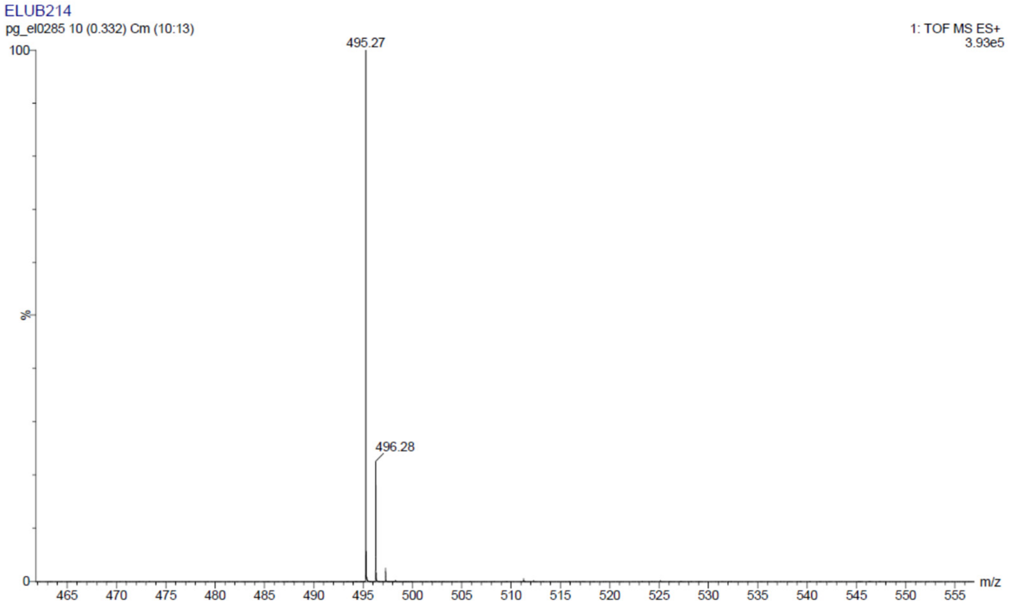

Figure S5e. MS (ESI) spectrum of ***t*-Bu-17-*p*-OH**

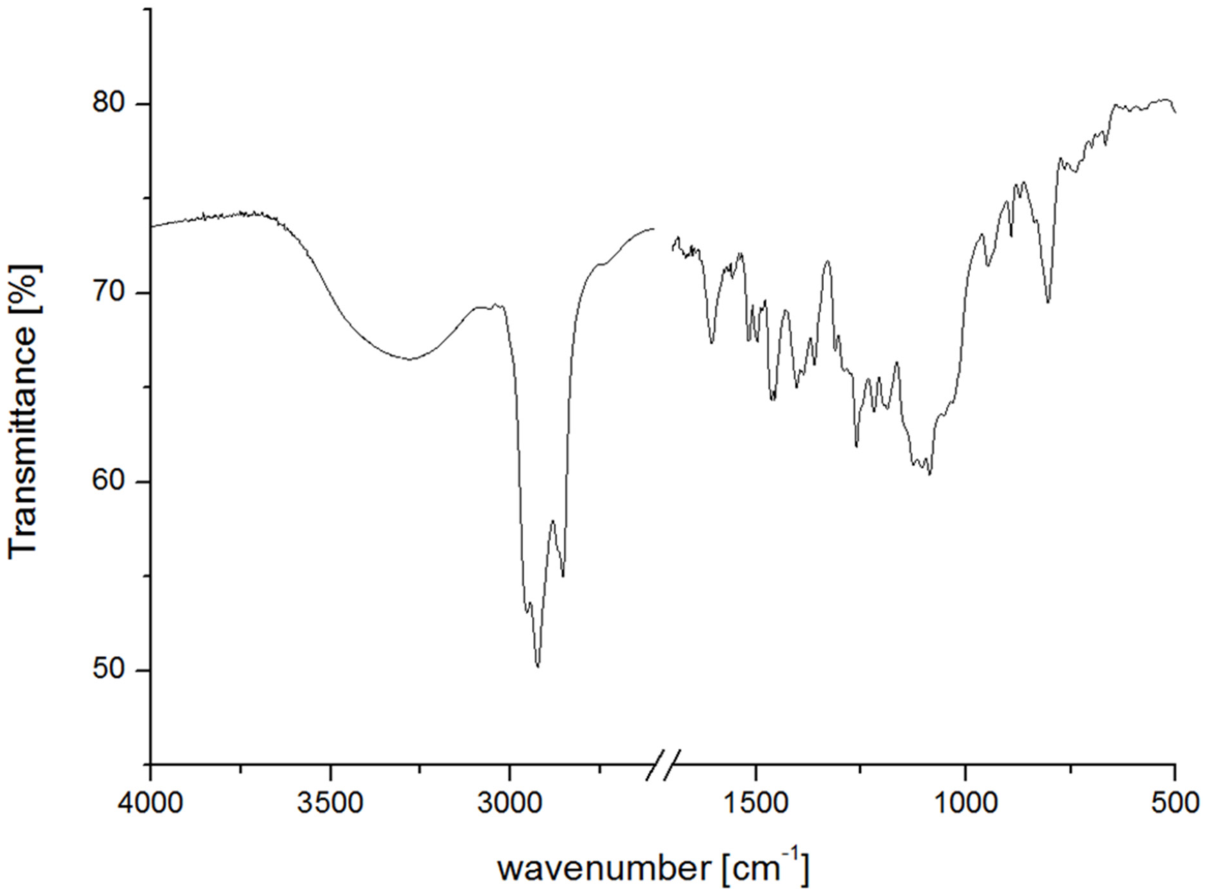

Figure S5f. FTIR spectrum (film) of ***t*-Bu-17-*p*-OH**

Table S1. Spectral - UV-Vis absorption - characteristics of *t*-Bu-19-*p*-OH, *t*-Bu-19-*o*-OH, *t*-Bu-20-al, *t*-Bu-20-ester, *t*-Bu-17-*p*-OH, *t*-Bu-19-Azo, *t*-Bu-19-Azo-O (acetonitrile)

Compound	λ [nm]	ε [$dm^3 \cdot mol^{-1} \cdot cm^{-1}$]
t-Bu-19-*p*-OH	442	3.19×10^4
t-Bu-19-*o*-OH	374	1.57×10^4
t-Bu-19-al	428	1.10×10^4
t-Bu-20-ester	473	9.55×10^3
t-Bu-17-*p*-OH	206<br>289	6.19×10^4<br>1.17×10^4
t-Bu-19-Azo	290<br>337<br>442	5.47×10^3<br>4.18×10^3<br>1.79×10^3
t-Bu-19-Azo-O	305<br>341	4.94×10^3<br>4.75×10^3

Table S2. The comparison of positions of bands λ_{max} [nm] in UV-Vis absorption and emission spectra of ***t*-Bu-19-*p*-OH** in different solvents (in parentheses: values of molar absorption coefficients, ε_{max} [$dm^3 \cdot mol^{-1} \cdot cm^{-1}$]). Stokes shift [nm] - the difference in the position of emission and absorption bands

Solvent	ACN	ACN:water#	DMSO	DMSO:water#	CH_2Cl_2	MeOH
			Absorption spectrum			
Azophenol form	-	-	359 ($9.27 \cdot 10^3$)	356 ($8.58 \cdot 10^3$)	-	-
Quinone-hydrazone form	442 (3.19×10^4)	446 ($3.31 \cdot 10^4$)	448 ($9.62 \cdot 10^3$)	450 ($8.81 \cdot 10^3$)	440 ($3.17 \cdot 10^4$)	454 ($1.89 \cdot 10^4$)
			Emission spectrum*			
	546	556	556	558	530	552
			Stokes' shift [nm]			
	104	110	108	108	90	98

ACN - acetonitrile; # organic solvent mixture with water (9:1, v/v); *excitation at wavelength corresponding to the long wave absorption maximum

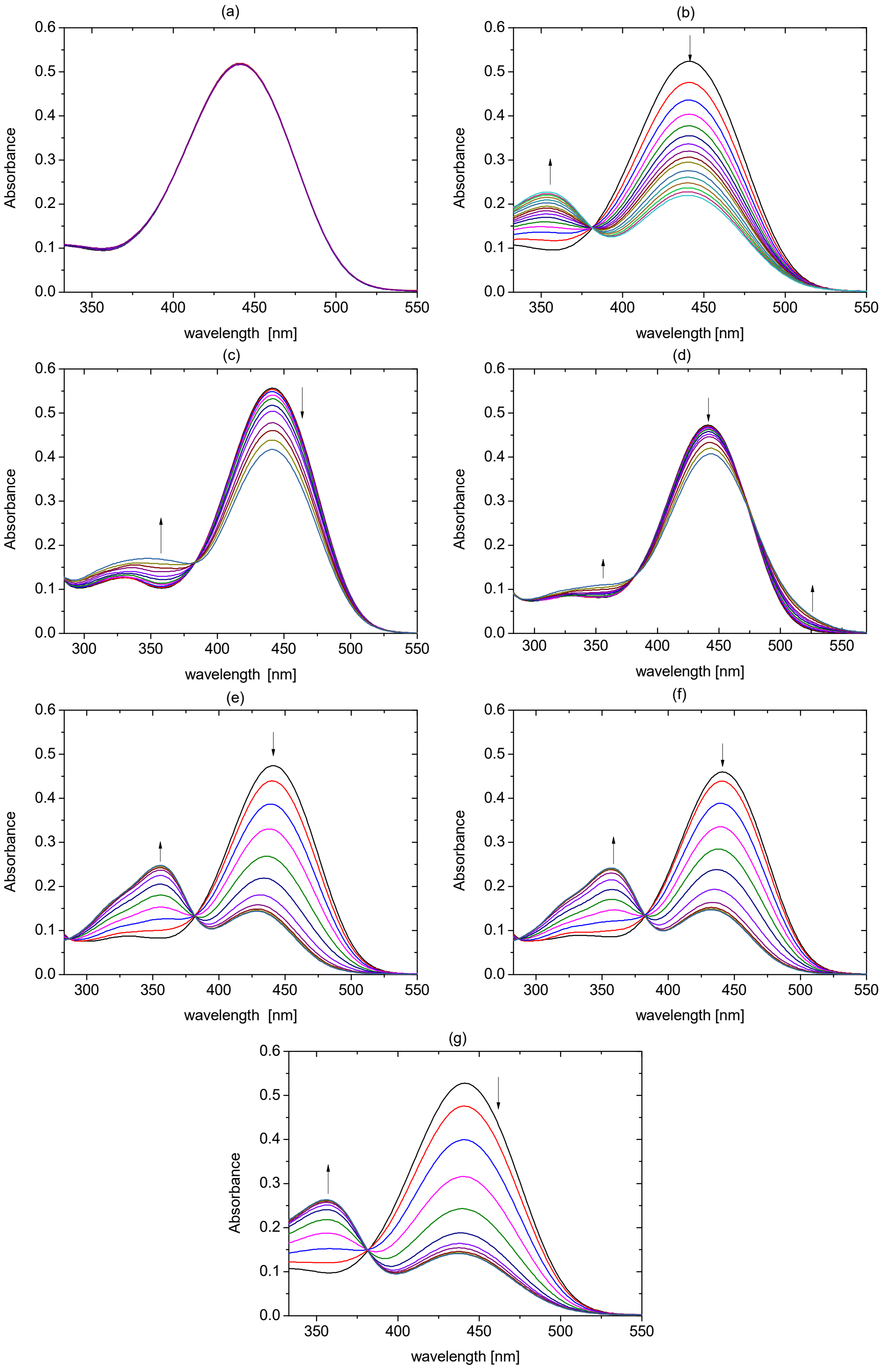

Figure S6. Changes in UV-Vis spectra upon titration of ***t*-Bu-19-*p*-OH** with metal perchlorates in acetonitrile: (a) ***t*-Bu-19-*p*-OH** (1.56×10^{-5} M), lithium ($0 - 1.99\times10^{-3}$ M); (b) ***t*-Bu-19-*p*-OH** (1.56×10^{-5} M), sodium ($0 - 1.6\times10^{-3}$ M); (c) ***t*-Bu-19-*p*-OH** (1.56×10^{-5} M), potassium ($0 - 4.3\times10^{-4}$ M); (d) ***t*-Bu-19-*p*-OH** (1.49×10^{-5} M), magnesium ($0 - 1.97\times10^{-3}$M); (e) ***t*-Bu-19-*p*-OH** (1.49×10^{-5} M), calcium ($0 - 2.62\times10^{-5}$ M); (f) ***t*-Bu-19-*p*-OH** (1.44×10^{-5} M), strontium ($0 - 2.62\times10^{-5}$ M); (g) ***t*-Bu-19-*p*-OH** (1.66×10^{-5} M), barium ($0 - 5.22\times10^{-5}$ M)

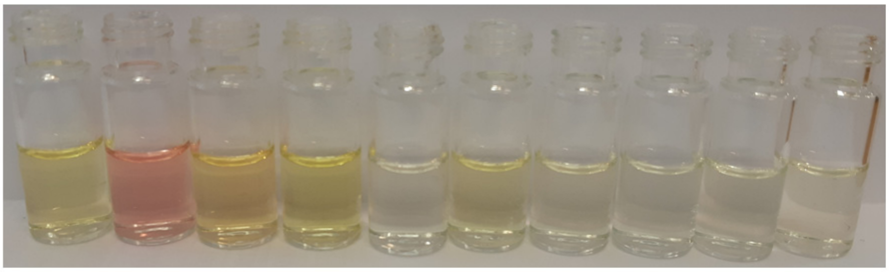

***t*-Bu-19-*p*-OH** acid base Li^{+} Na^{+} K^{+} Mg^{2+} Ca^{2+} Sr^{2+} Ba^{2+}

Figure S7**.** The influence of the presence of acid (*p*-toluenesulfonic acid), base (tetra-n-butylammonium hydroxide) and metal perchlorates on the color of solution of ***t*-Bu-19-*p*-OH** in acetonitrile **(**quantitative probe acid, base and metal perchlorates were added in excess as solids to solution of crown 1.56×10^{-5} M)

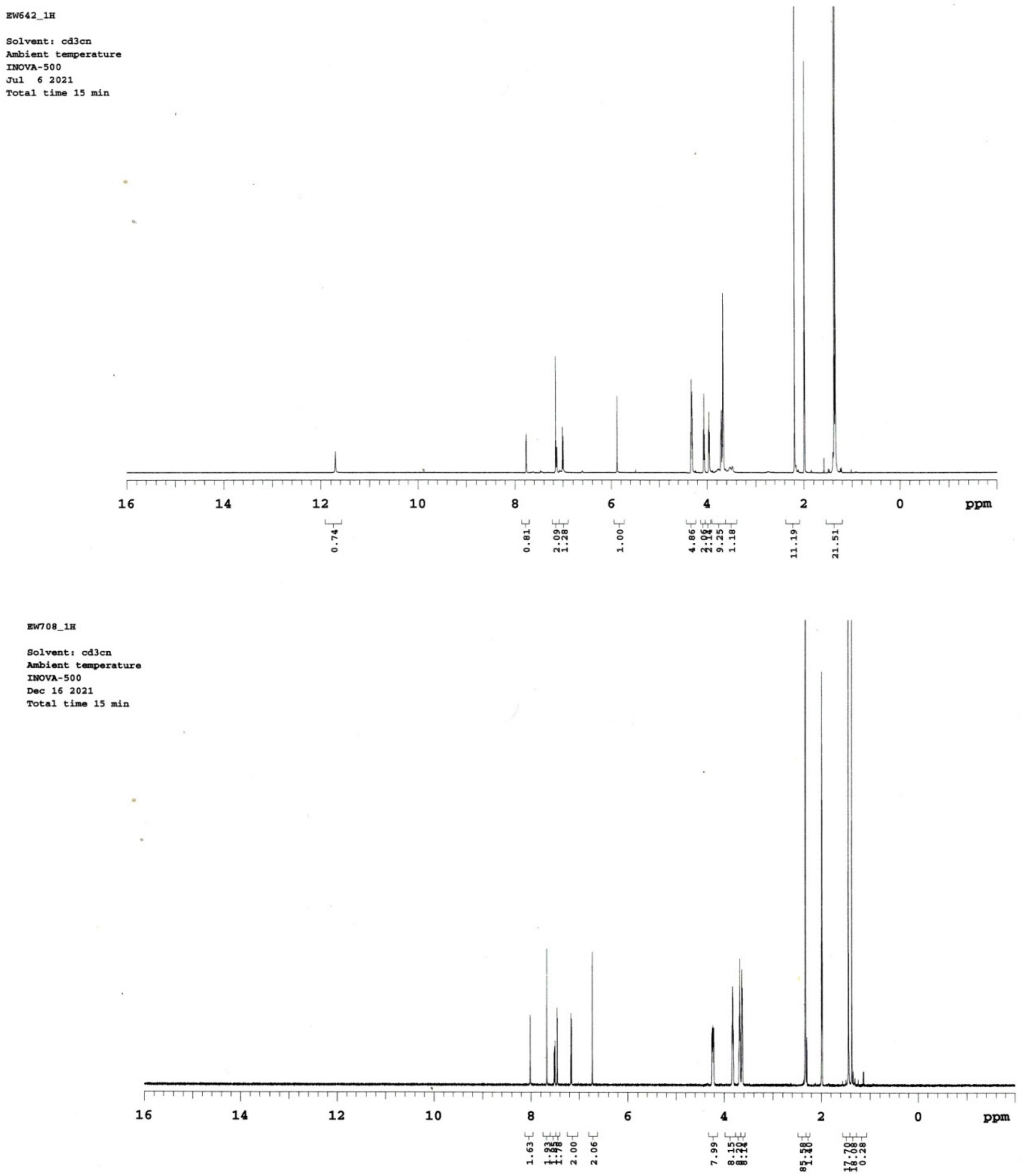

Figure S8. ^{1}H NMR spectrum of ***t*-Bu-19-*p*-OH** (8.60 $\times 10^{-3}$ M) - top and spectrum registered in the presence of 10-fold excess of sodium perchlorate - bottom (acetonitrile-d_3).

Table S3. Comparison of stability constants (log K) of complexes (1:1) of 19-membered crowns in acetonitrile

entry	metal perchlorate	***t*-Bu-19-AZB***	***t*-Bu-19-AZB-O**	***t*-Bu-19-*p*-OH**	***t*-Bu-19-*o*-OH**
1	Li	~2.3	-	-	-
2	Na	~4.3	3.42±0.03	3.51±0.39	3.45±0.02
3	K	~3.9	-	3.36±0.62	3.12±0.15
4	Mg	~2.4	-	2.63±0.36	-
5	Ca	~4.8	4.59±0.43	6.38±0.29	7.26±0.13
6	Sr	~4.9	4.92±0.02	6.42±0.16	5.15±0.57
7	Ba	~5.2	4.63±0.45	6.03±0.09	4.82±0.59

- neglible spectral changes; complexation proces was carried out for crown being a mixture of *Z* and *E* isomers

* estimated values for mixture of isomers

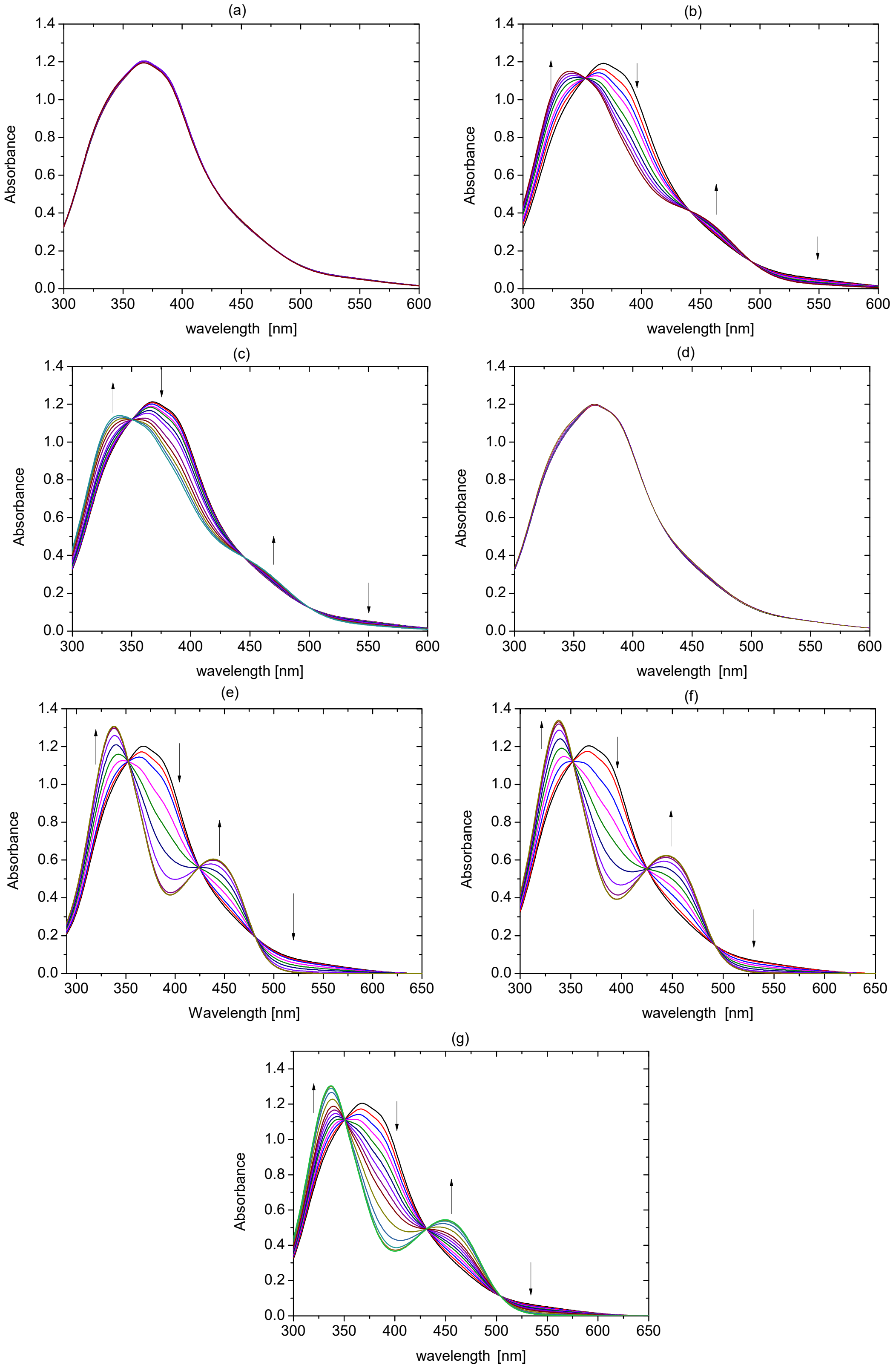

Figure S9. Changes in UV-Vis spectra upon titration of ***t*-Bu-19-*o*-OH** (7.33×10^{-5} M) with metal perchlorates in acetonitrile: (a) lithium (0 – 1.55×10^{-3} M); (b) sodium (0 – 5.88×10^{-4} M); (c) potassium (0 – 6.14×10^{-4} M); (d) magnesium (0 – 1.77×10^{-3} M); (e) calcium (0 – 1.19×10^{-4} M); (f) strontium (0 – 1.05×10^{-4} M); (g) barium (0 – 1.08×10^{-4} M)

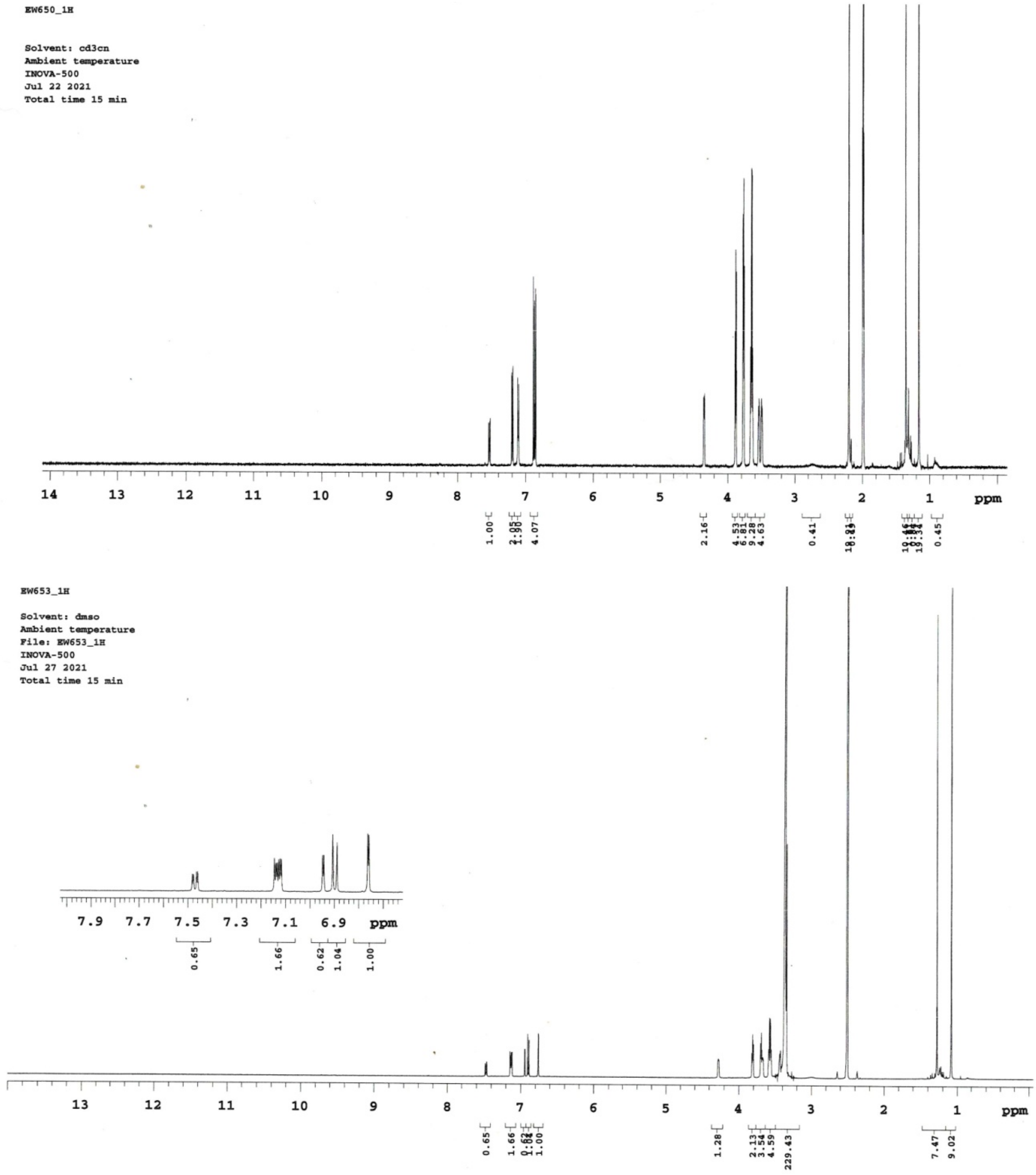

Figure S10. 1H NMR spectra of t-Bu-19-Azo. Top: crown concentration 5.9×10-3 M, ratio of isomers E 31% and Z 69% in acetonitrile-d3; bottom: crown concentration 5.6×10-3 M, ratio of isomers E 45% and Z 55% in DMSO-d_6.

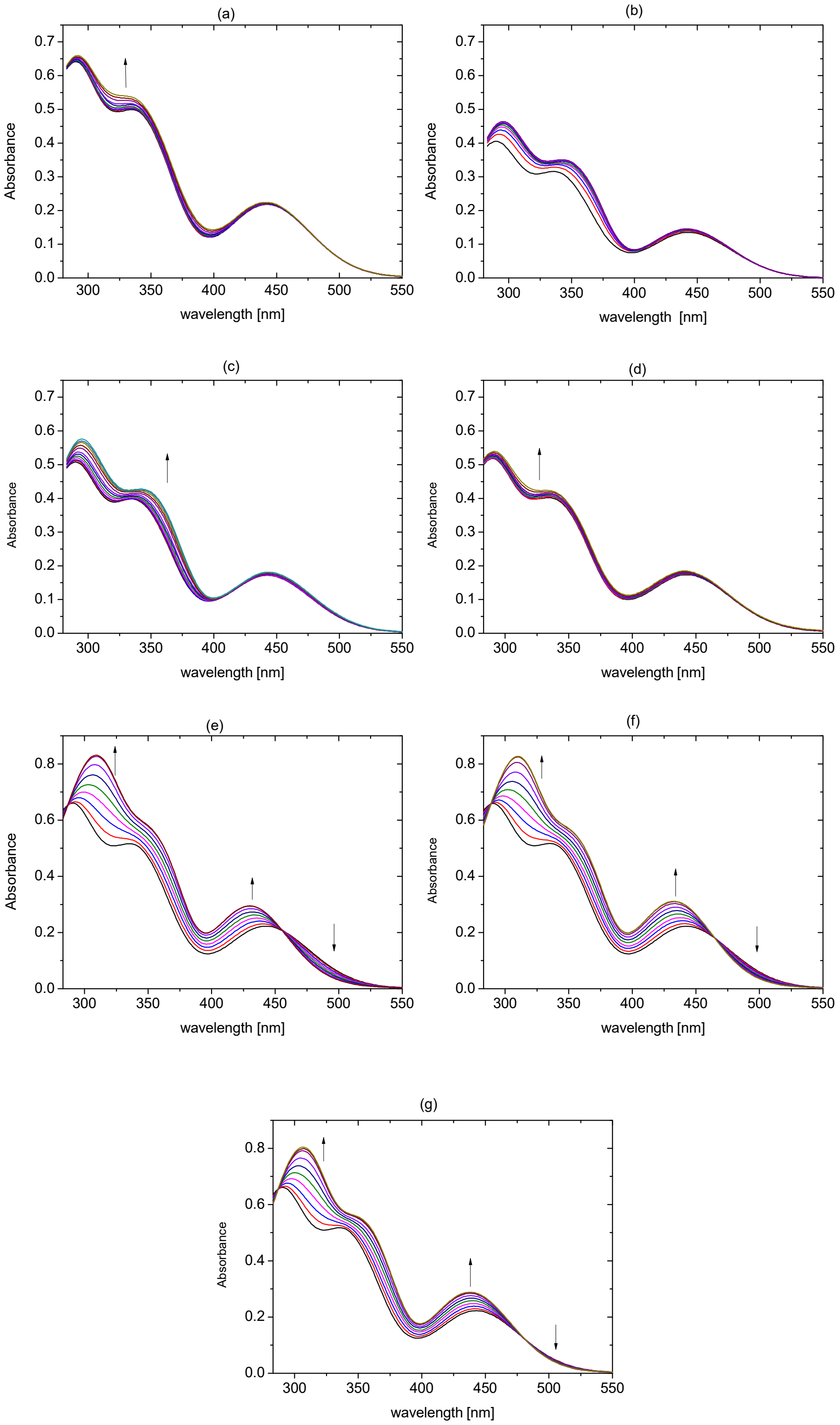

Figure S11. Changes in UV-Vis spectra upon titration of ***t*-Bu-19-Azo** with metal perchlorates in acetonitrile: (a) ***t*-Bu-19-Azo** (1.17×10^{-4} M), lithium ($0 - 2.47\times10^{-3}$ M); (b) ***t*-Bu-19-Azo** (8.17×10^{-5} M), sodium ($0 - 3.87\times10^{-4}$ M); (c) ***t*-Bu-19-Azo** (9.05×10^{-5} M), potassium ($0 - 3.46\times10^{-4}$ M); (d) ***t*-Bu-19-Azo** (9.05×10^{-5} M), magnesium ($0 - 1.73\times10^{-3}$ M); (e) ***t*-Bu-19-Azo** (1.17×10^{-4} M), calcium ($0 - 5.38\times10^{-5}$ M); (f) ***t*-Bu-19-Azo** (1.17×10^{-4} M), strontium ($0 - 6.08\times10^{-5}$ M); (g) ***t*-Bu-19-Azo** (1.17×10^{-4} M), barium ($0 - 5.93\times10^{-5}$ M)

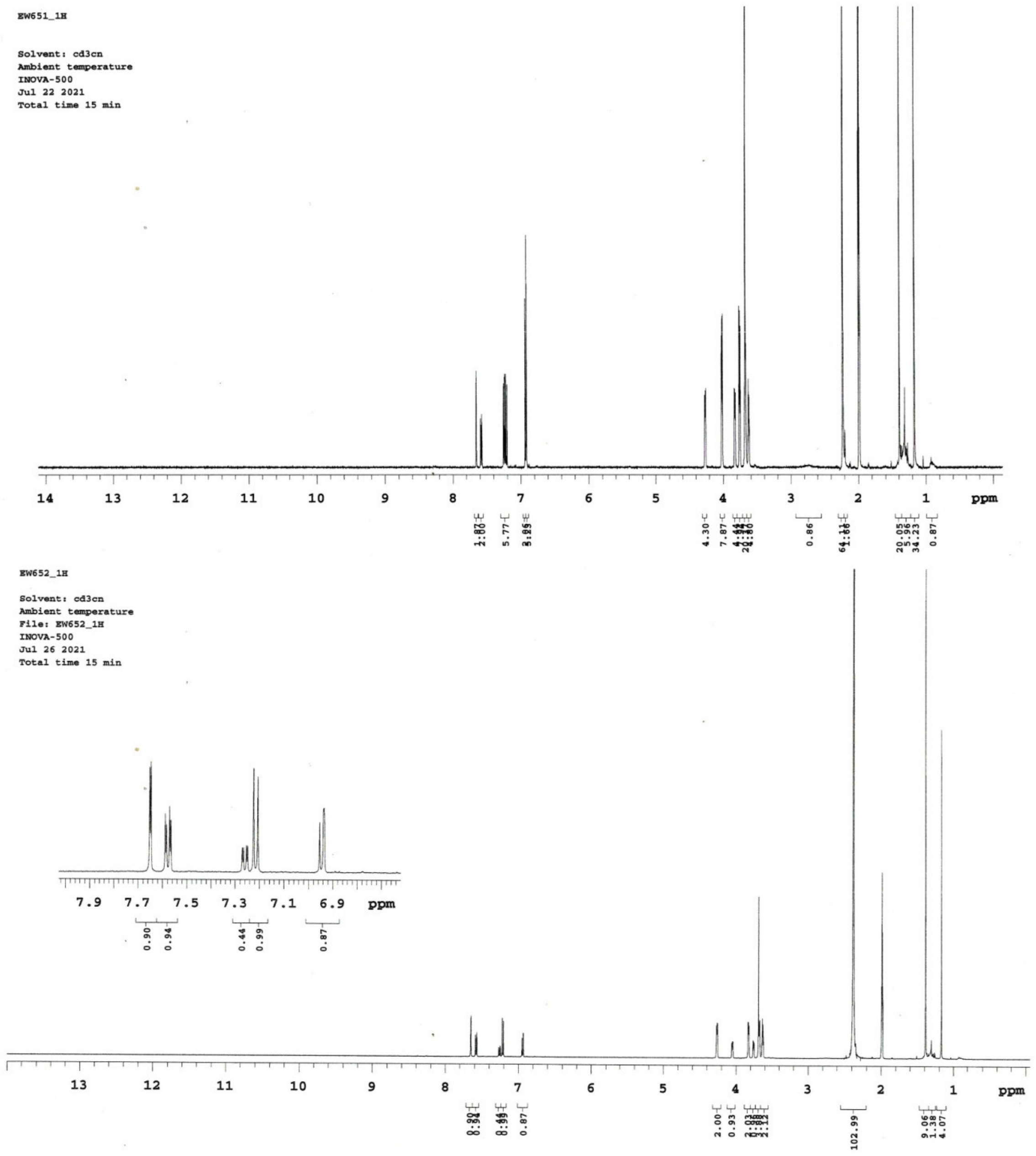

Figure S12. ^{1}H NMR spectra of ***t*-Bu-19-Azo** (5.9×10^{-3} M) registered in the presence of equimolar amount of sodium perchlorate (top) and 10-fold excess of this salt (bottom) in acetonitrile-d_3.

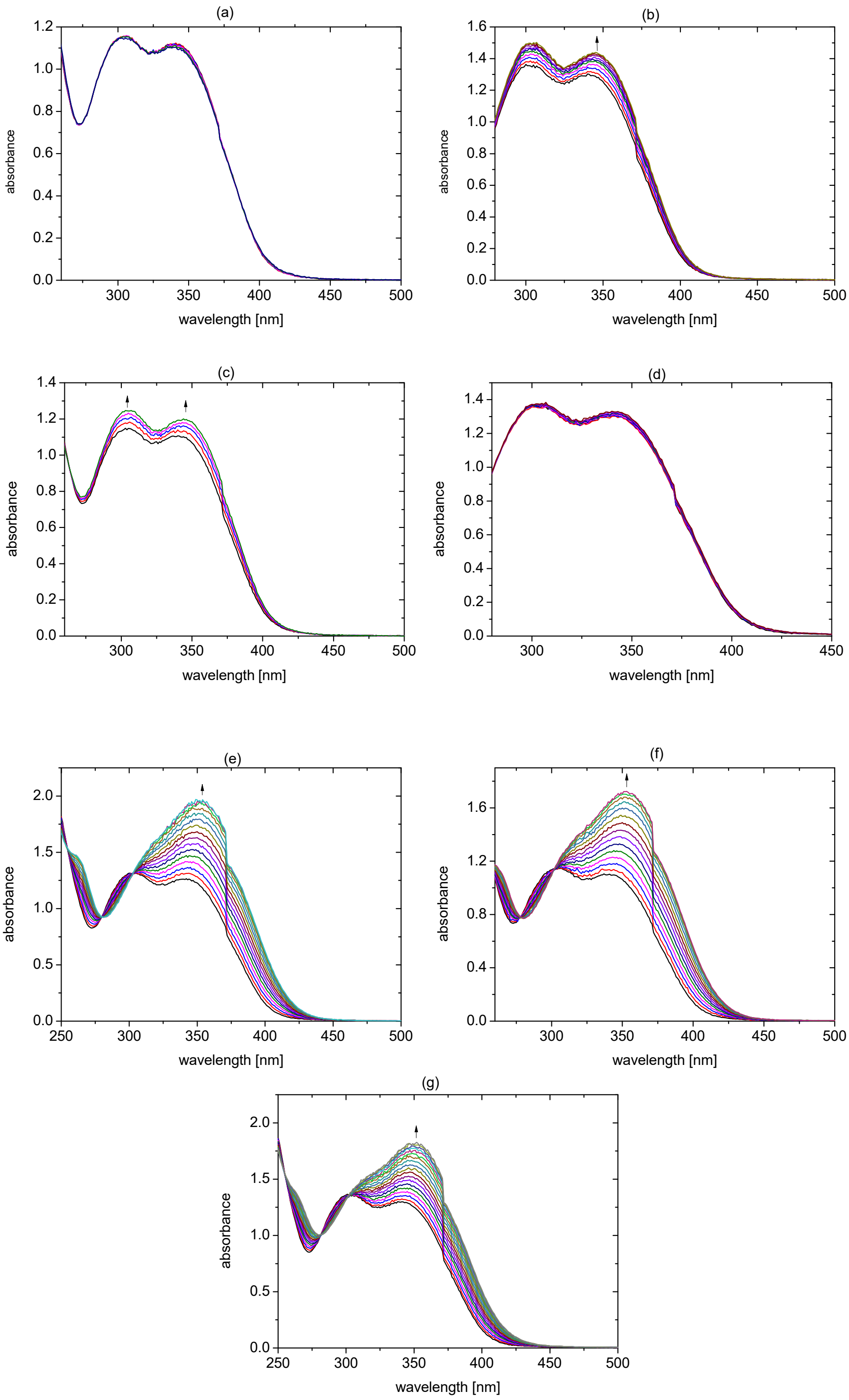

Figure S13. Changes in UV-Vis spectra upon titration of ***t*-Bu-19-Azo-O** with metal perchlorates in acetonitrile: (a) ***t*-Bu-19-Azo-O** (2.26×10^{-4} M), lithium ($0 - 1.98\times10^{-4}$ M); (b) ***t*-Bu-19-Azo-O** (2.83×10^{-4} M), sodium ($0 - 4.47\times10^{-4}$ M); (c) ***t*-Bu-19-Azo-O** (2.26×10^{-4} M), potassium ($0 - 3.21\times10^{-4}$ M); (d) ***t*-Bu-19-Azo-O** (2.83×10^{-4} M), magnesium ($0 - 2.87\times10^{-3}$ M); (e) ***t*-Bu-19-Azo-O** (2.83×10^{-4} M), calcium ($0 - 1.93\times10^{-4}$ M); (f) ***t*-Bu-19-Azo-O** (2.26×10^{-4} M), strontium ($0 - 2.03\times10^{-4}$ M);(g) ***t*-Bu-19-Azo-O** (2.83×10^{-4} M), barium ($0 - 2.24\times10^{-4}$ M)

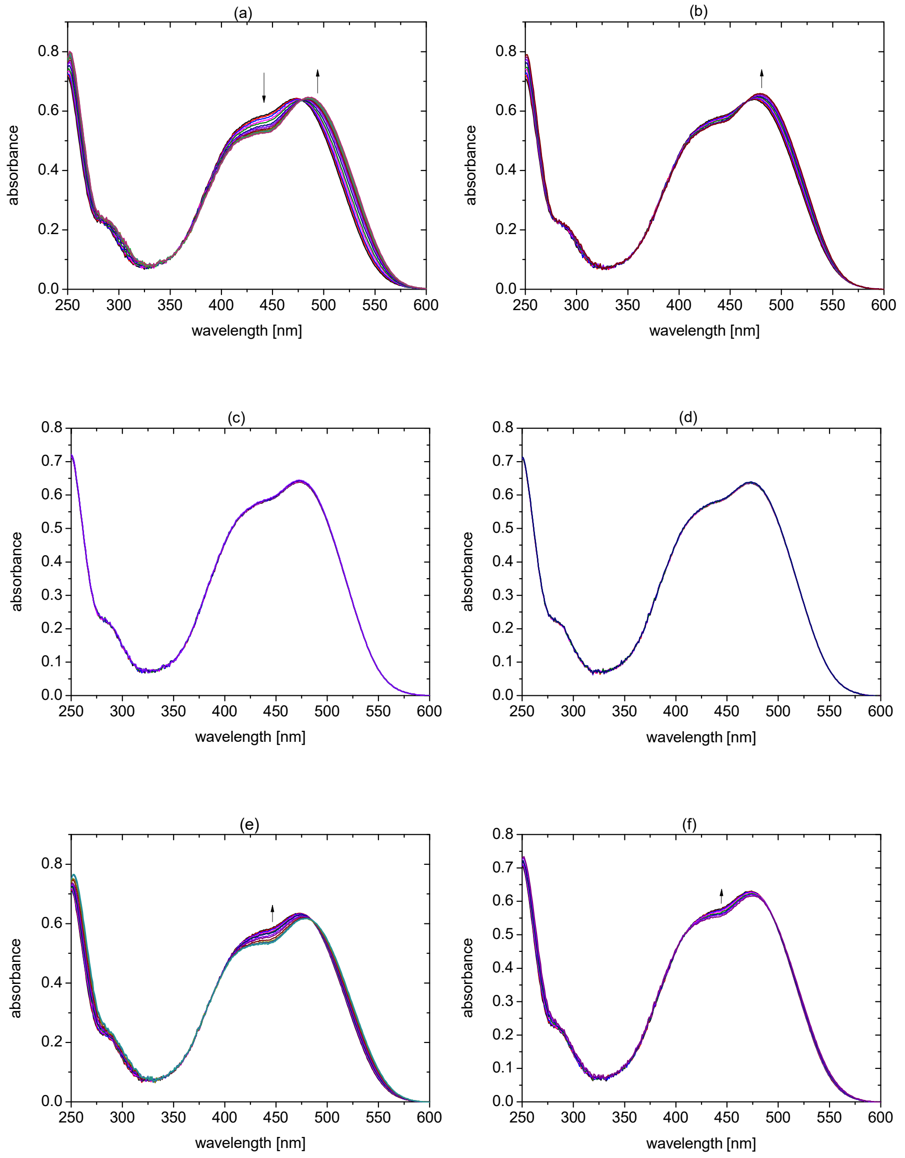

Figure S14. Exemplary spectra showing changes in UV-Vis upon titration of ***t*-Bu-20-ester** (1.02×10^{-4} M) with metal perchlorates in acetonitrile: (a) lithium (0–3.48×10^{-3} M); (b) sodium (0–2.14×10^{-3} M); (c) potassium (0–1.12×10^{-4} M); (d) magnesium (0 –7.27×10^{-4} M); (e) calcium (0–1.03×10^{-3} M); (f) strontium (0–5.77×10^{-4} M)
